# Highly regioselective 6-*exo-dig* iodo/bromo cyclizations of functionalized 5-amino propargyl pyrimidinones: an efficient synthesis of functionalized pteridines†

**DOI:** 10.1039/d3ra05651a

**Published:** 2023-10-31

**Authors:** Rayees Ahmad Naikoo, Rupesh Kumar, Rashmi Sharma, Dinesh Mahajan, Gaurav Bhargava

**Affiliations:** a Department of Chemical Sciences, I. K. Gujral Punjab Technical University Kapurthala Punjab 144603 India gaurav@ptu.ac.in rsharma082@gmail.com; b Translational Health Science and Technology Institute Faridabad Haryana India

## Abstract

The manuscript describes the highly regioselective 6-*exo-dig* iodo/bromo cyclization of functionalized *N*-propagyl-amino-pyrimidinones under ambient conditions. The cyclization afforded functionalized pteridines in excellent yields. The optimized procedures are mild, operationally simple and working successfully with different substrates. The synthesis of functionalized pteridines is of great significance because of their potential pharmacological profile.

## Abstract 2

This work describes the highly regio-selective 6-*exo-dig* iodo/bromo cyclization of functionalized *N*-propagyl-aminopyrimidinones with good to excellent yields under ambient conditions.

## Graphical abstract

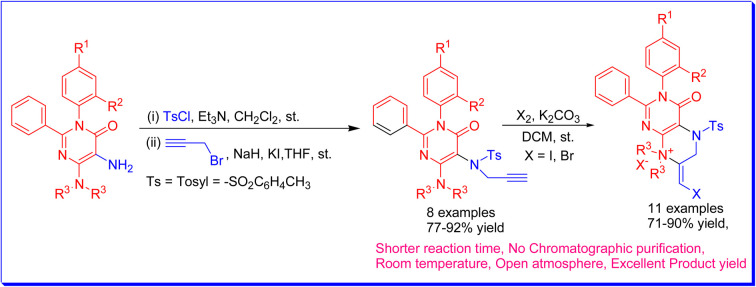

## Introduction

Bicyclic pyrimidinones, condensed with other heterocyclic systems at different positions, have been extensively explored and evaluated for a wide range of biological properties.^1^ Pteridines are shown to be highly biologically active in every element of the growth and development of living things, including the treatment of cancer, heart disease, neurotransmitter generation, and amino acid metabolism.^2–4^ Moreover, a number of prevalent diseases including inflammatory disorders, autoimmune processes, neurological diseases, and birth defects have been attributed to the problems in the synthesis, nutritional availability, and/or metabolism of these compounds.^3–14^ Functionalized pteridines have also been explored for the treatment of fibroproliferative disorders, hepatitis C,^15,16^ and vascular disorders, *etc.*^12,17–21^

**Fig. 1:**
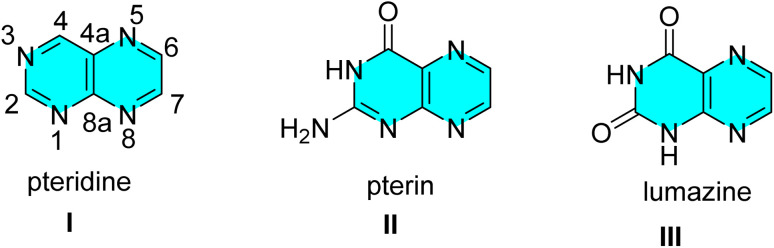
Structures of pteridines.

A group of heterocyclic compounds known as pteridine, pyrazino[2,3-*d*] pyrimidines are composed up of condensed pyrimidine/pyrimidinone and pyrazine rings.^22^ Most naturally produced pteridines referred to as pterins (II) or generally named as 2-amino-4(3*H*)pteridone belong to a family of nitrogen heterocyclic compounds. The term “pteridine” refers to pyrazino[2,3-*d*] pyrimidine nucleus structurally, with the numbering of the ring system shown below in (I).^23,24^ The process of condensation of 4,5-diamino pyrimidine-2,6-dione with various dicarbonyl compounds has been exploited to synthesize pteridines of class III known as lumazines (Fig. 1).^23,25–29^

**Fig. 2:**
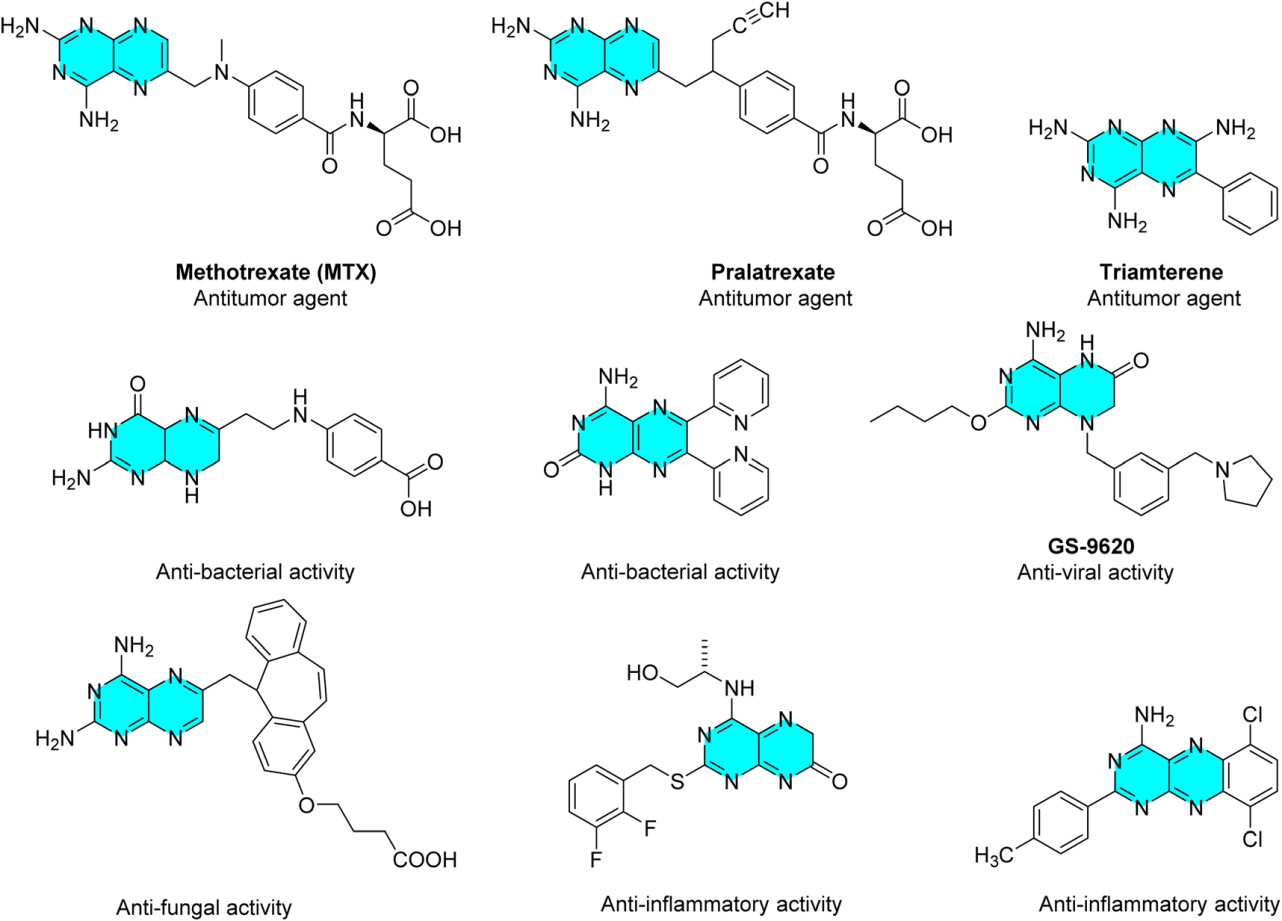
Biological applications of some pteridines.

The synthesis of such functionalized pteridines with a variety of substitutions at different locations becomes crucial due to their potential pharmacological profile.^30^ As part of our ongoing interest in heterocyclic chemistry, we have previously looked into the synthesis of tricyclic pyrimidinones condensed benzodiazepines,^31,32^ pyrimidino[thiazenes],^33^ condensed lactams and thiazole condensed benzodiazepines^34–36^ among other compounds. The present manuscript describes the synthesis of functionalized 1,2,4,5-tetrasubstituted pyrimidinones and their 6-*exo dig* halocyclization to yield a variety of functionalized pteridines. The current approach has a number of benefits, including high yield, simplicity, and the provision of functionalized pteridines that can be converted into various heterocyclic systems (Fig. 2).

## Results & discussion

**Scheme 1:**
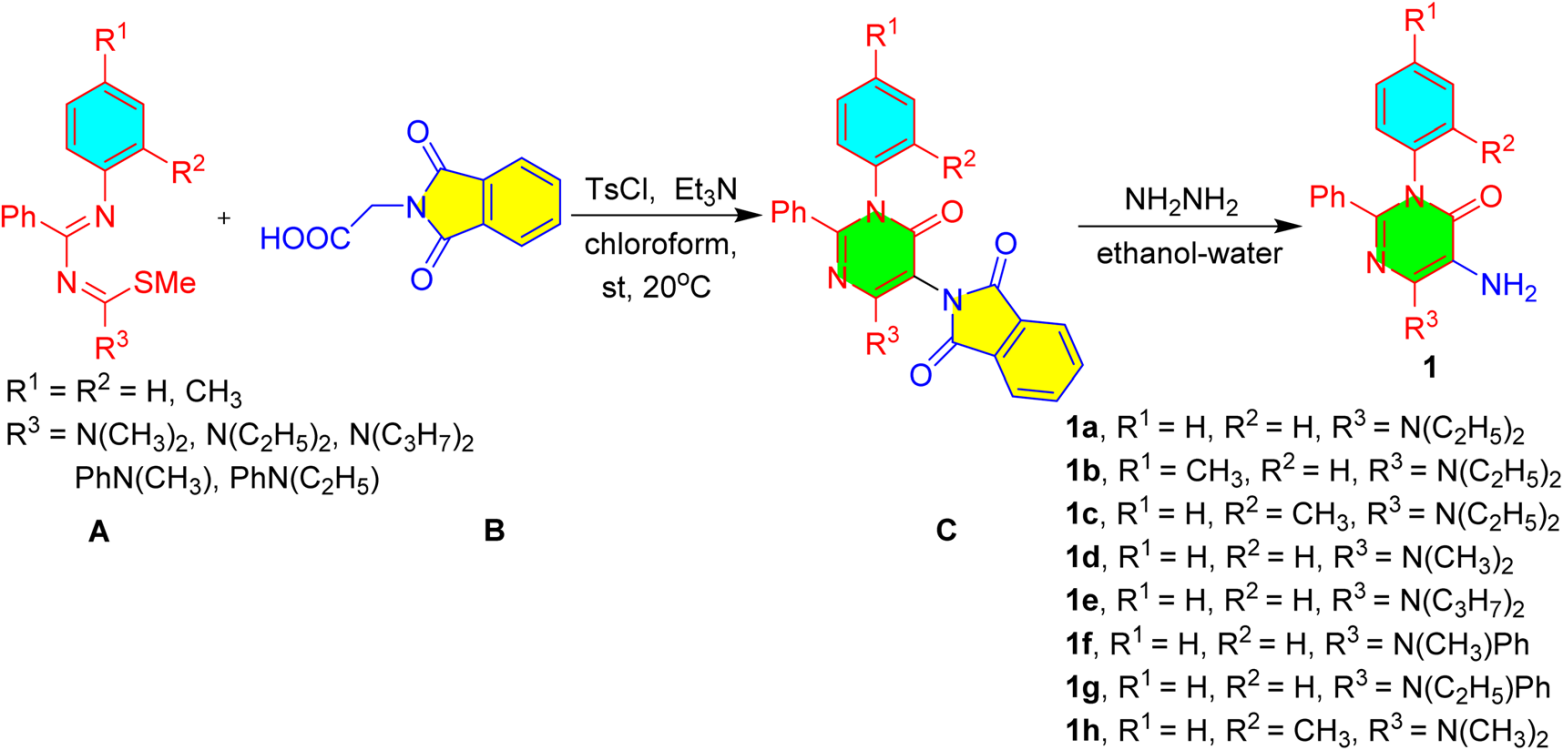
Synthesis of starting materials, 5-amino-pyrimidinones.

The functionalized 5-amino pyrimidinones, 1a–h were prepared by the reaction of phthloylglycine, B with functionalized 1,3-diazabuta-1,3-dienes, A and their subsequent amino deprotection reactions of C using hydrazine hydrate and ethanol (Scheme 1).^37^

**Scheme 2:**
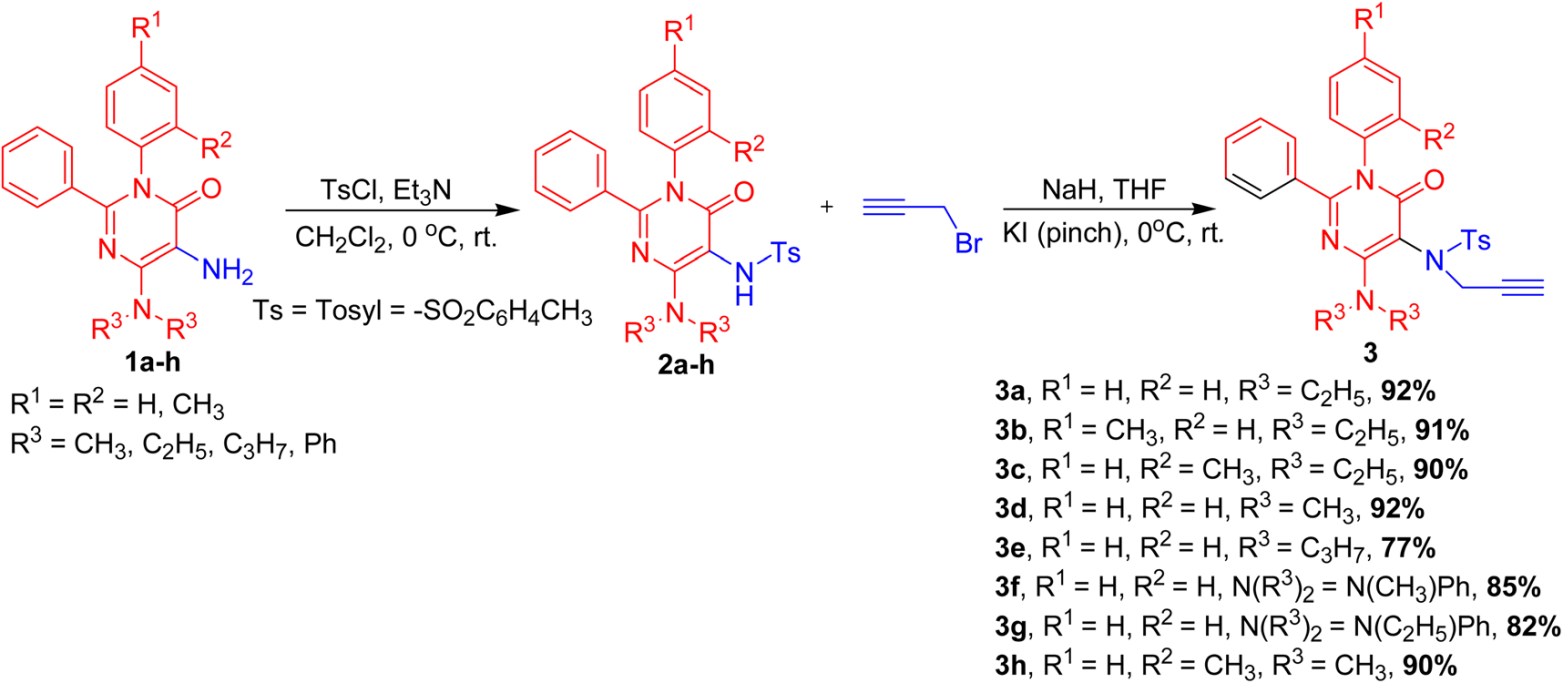
Synthesis of *N*-propagyl-*N*-(4-dialkyl/aryl-amino-6-oxo-1,2-diaryl-1,6-dihydro-pyrimidin-5-yl)-arylsulfonamides, 3a–h.

These functionalized 5-amino pyrimidinones, 1a–h were explored in 6-*exo dig* halocyclization reactions to yield 4-oxo-2,3-diaryl-pteridin-8-ium halide, 4a–k in excellent yields. The synthetic methodology involved the initial mono-tosylation of functionalized 5-amino pyrimidinones, 1a–h using tosyl chloride and mild base as triethylamine to yield *N*-(4-diaryl/alkylamino-6-oxo-1,2-diaryl-1,6-dihydro-pyrimidin-5-yl)-4-methyl-benzenesulfonamides, 2a–h. These mono-aryl-sulphonated 5-amino pyrimidinones, 2a–h were explored in mono-propargylation to provide a series of *N*-propargyl-*N*-(4-dialkyl/aryl-amino-6-oxo-1,2-diaryl-1,6-dihydro-pyrimidin-5-yl)-aryl sulfonamides, 3a–h in excellent yields (77–92% yield, Scheme 2).

These functionalized pyrimidinones, 3a–h were explored in 6-*exo-dig* halocyclization reactions. The reaction resulted in the formation of 4-oxo-2,3-diaryl-pteridin-8-ium halide, 4a–k in good to excellent yields. Different solvents such as DCM, toluene, acetonitrile, *etc.*, and different halogenated agents such as NCS, NBS, Br_2_, I_2,_*etc* were attempted for better yield and selectivity in the synthesis of functionalized 4-oxo-2,3-diaryl-pteridin-8-ium halide, 4a–k. The results are summarized in Table 1. It has been found that the iodocyclization occurs efficiently using I_2_ (3 eq.) in DCM (20 mL) and the reaction gave poor yield in other tested solvents such as acetonitrile and toluene. The 6-*exo-dig* halocyclizations of functionalized pyrimidinones using alternate iodocyclization agents such as NIS afforded undesired products (Table 1, entry 1). The iodocyclization reactions also occurred efficiently in the absence of base (Table 1; entries 8–14). Next, we optimized the reaction conditions for 6-*exo-dig* bromo cyclizations using different brominating agents such as NBS, Br_2_, *etc.* The 6-*exo-dig* bromocyclization afforded 4-oxo-2, 3-diaryl-pteridin-8-ium bromide in good yields using Br_2_ (3 eq.) in DCM (20 mL) (Table 1, entries 6, and 12–15). The 6-*exo-dig* bromocyclization led to poor yields of product when a higher amount of bromine (4 to 6 eq.) was used during haloaminations. The 6-*exo-dig* bromocyclizations were inefficient and undesired products were found when NBS was used as a halogen source under different reaction conditions (Table 1, entries 4 and 5). Moreover, the chloro–amination reactions were unsuccessful using *N*-bromosuccinamide (NCS) was used as a halogen source in attempted 6-*exo-dig* chloroamination reactions (Table 1, entry 3).

**Table 1:** Optimization of the reaction conditions for 6-*exo-dig* halocyclizations

S. no	Pyrimidinone	Reaction conditions				Reaction time^b^	Yields^a^ (%)
		Reagent	Eq.	Base (5 eq.)	Solvent (20 mL)		
1	3a	NIS	4	K_2_CO_3_	DCM	—	—
2	3a^c^	I_2_	3	K_2_CO_3_	DCM	20 min	86
3	3a	NCS	4	K_2_CO_3_	DCM	—	—
4	3a	NBS	4	K_2_CO_3_	DCM	—	—
5	3a	NBS	4	NaH	DCM	—	—
6	3a	Br_2_	2.5	K_2_CO_3_	DCM	20 min	79
7	3a	I_2_	4.5	*t*-BuOK	THF	—	—
**8**	3a^c^	**I**_**2**_	**3**	**—**	**DCM**	**20 min**	**89**
9	3a	I_2_	3.5	—	Toluene	3 h	55
10	3a	I_2_	3.5	—	THF	1 h	50
11	3a	I_2_	3.5	—	Acetonitrile	1.5 h	40
**12**	3a	**Br**_**2**_	**3**	**—**	**DCM**	**20 min**	**84**
13	3a	Br_2_	3	—	Toluene	3 h	53
14	3a	Br_2_	3	—	THF	3 h	49
15	3a	Br_2_	3	—	Acetonitrile	3 h	35

a Isolated yields after purification.

b Reaction time.

c Dry DCM used as a solvent.

We next investigated these 6-*exo-dig* halocyclization reactions using a variety of functionalized pyrimidinones. Different pyrimidinones, 3a–h with a variety of substituents such as dimethyl, diethyl, dipropyl, *etc.* at the C-4 position were studied in these halocyclization reactions. The reactions resulted in the formation of 4-oxo-2,3-diaryl-pteridin-8-ium halide 4a–k in good to excellent yields (Table 2, entries 1–11). The various substituents at the C-1 or C-2 position did not change the yield of the product of these halocyclization reactions (Table 2; entries 1–11). The 6-*exo-dig* halocyclization reactions tolerate a variety of steric bulk at the C-4 position (Table 2; entries 3–7). Functionalized pyrimidinones with a dimethyl or diethyl amino group at the C-4 position resulted in efficient 6-*exo-dig* cyclizations (Table 2; entries 1–4 and 8–11). With dipropyl amine at its C-4 position, the halo amination of 3e took a relatively longer reaction time and yielded 4e with a slightly lower yield (Table 2; entry 5). With a hindered secondary amine (*N*-aryl methyl/ethyl amine) at the C-4 position, the 2,3-dialkyl-5-propynylsulfanyl-3*H*-pyrimidin-4-ones, 3f & 3g effectively accomplished 6-*endo*-dig cyclization reactions to provide 4f, g in good yields (Table 2; entries 6 and 7). These experimental findings demonstrate that the various sterically hindered amines at the C-4 position are successfully tolerated by the 6-*exo-dig* haloamination reactions of pyrimidinones, 3a–h. (Table 2; entries 5–7). The yield decreases with an increase in steric bulk at the C-4 position. The bromocyclization afforded comparatively lower yields of 4-oxo-2,3-diaryl-pteridin-8-ium halide owing to the more reactive nature of the bromine (Table 2, entries 8–11). All these reactions resulted in the formation of 4-oxo-2,3-diaryl-pteridin-8-ium halide, 4a–k, and competitive 7-*endo dig* cyclized products were not formed. The Impure compounds, 4a–k were purified by using a solvent mixture of dichloromethane and diethyl ether (1 : 9) without performing any column chromatography.

**Table 2:** 6-*exo-dig* haloamination reactions of functionalized pyrimidinones, 3a–h

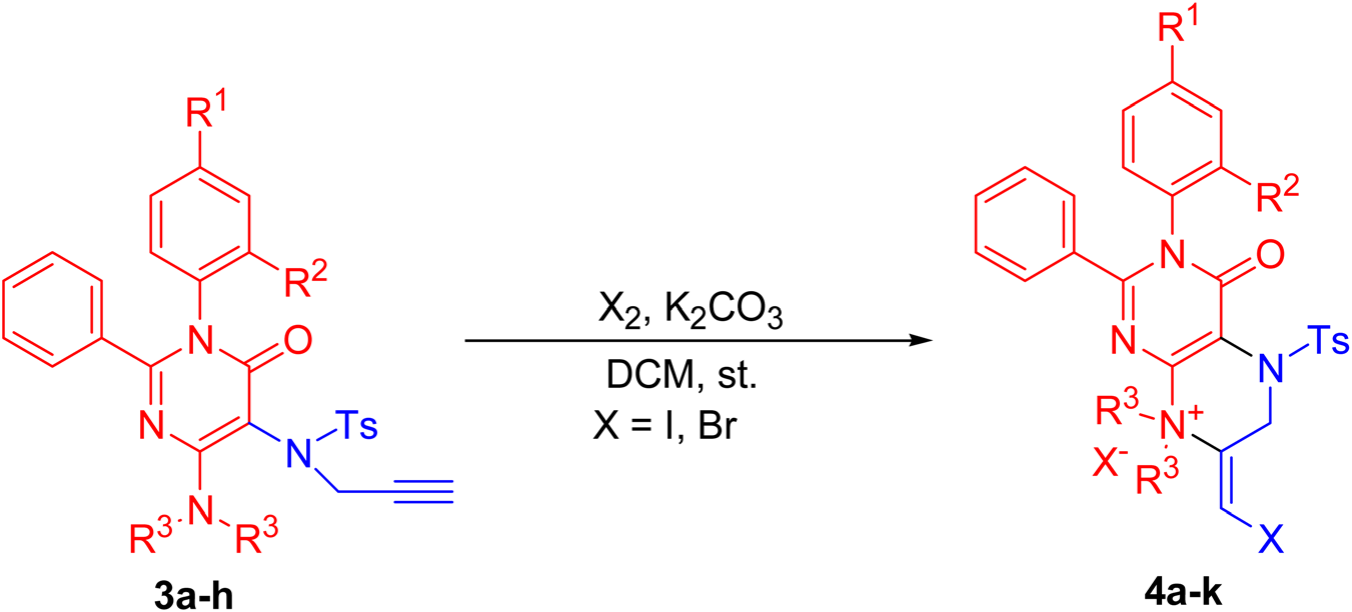							
S. no	R^1^	R^2^	R^3^	X	Substrate^b^	Product^c^	Yield^a^ (%)
**1**	**H**	**H**	**C**_**2**_ **H**_**5**_	**I**	3a	4a	**89**
2	CH_3_	H	C_2_H_5_	I	3b	4b	87
3	H	CH_3_	C_2_H_5_	I	3c	4c	88
4	H	H	CH_3_	I	3d	4d	90
5	H	H	C_3_ H_7_	I	3e	4e	71
6	H	H	CH_3_ & Ph	I	3f	4f	84
7	H	H	C_2_H_5_ & Ph	I	3g	4g	82
8	H	CH_3_	CH_3_	Br	3h	4h	86
9	H	H	CH_3_	Br	3d	4i	85
10	H	H	C_2_H_5_	Br	3a	4j	84
11	CH_3_	H	C_2_H_5_	Br	3b	4k	82

a Isolated yields after purification.

b Reaction time 20 min.

c Starting substrates (3a–g) taken = 500 mg, 0.870–1.000 mmoles.

**Scheme 3:**
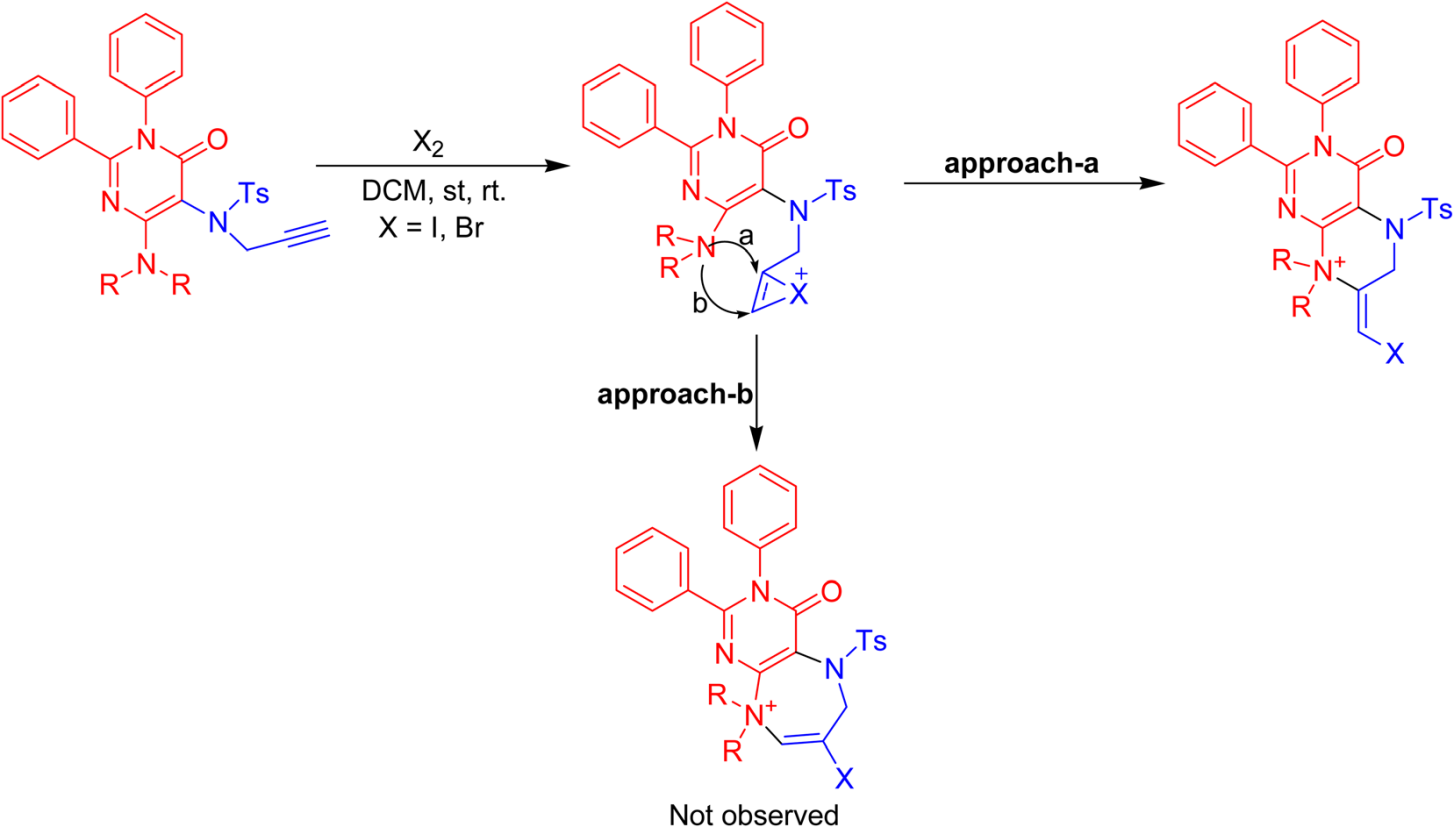
Mechanism for the formation of hexahydropteridin-8-ium derivatives, 4a–k.

The plausible mechanism involved the iodonium ion's coordination with the triple bond of the *N*-propargyl of the pyrimidinone ring during its initial formation. The subsequent *exo-dig* nucleophilic attack of the C-4 substituted secondary amino group results in the production of the 4-oxo-2,3-diaryl-3,4,5,6,7,8-hexahydro-pteridin-8-ium halide in good yields. Approach-a for haloamination is preferred while competitive approach-b is disfavored due to the development of a more stabilised six-membered fused pyrazine ring than the competitive seven-membered fused diazepine ring (Scheme 3).

## Conclusion

In summary, an efficient regioselective protocol for the formation of functionalized pteridines has been reported. The operational simplicity, shorter reaction time, good substrate scope, column chromatography-free approach, and regioselectivity are the attractive features of the present method. Further exploration of the full scope of these reactions and their extension to other arenes and heteroarenes will be reported in due course.

## Experimental section

### General procedure for the formation of *N*-(4-dialkylamino-6-oxo-1,2-diaryl-1,6-dihydro-pyrimidinin-5-yl)-4-methyl-benzenesulfonamide (2a–h)

To a solution of 5-amino pyrimidinones 1a–h (2 g, 1.950–2.550 mmoles) and triethylamine (3 eq.) in dry CHCl_3_ (50 mL) at 0 °C, was added dropwise a solution of *p*-TsCl (2.0 eq.) mixed in dry chloroform. The advancement of the reaction was checked by tlc. At the end of the reaction (overnight stirring), a usual workup was carried out using water and chloroform. The organic layers were combined, dried over sodium sulfate, and concentrated to get the crude product. The impure crude product was loaded into the column and purified by using ethyl acetate and hexane (2 : 8) as an eluent. The crude compounds were further purified using a mixture of 10% dichloromethane in diethyl ether to obtain *N*-(4-dialkylamino-6-oxo-1,2-diaryl-1,6-dihydro-pyrimidinin-5-yl)-4-methyl-benzenesulfonamide (2a–h) as pure compounds in good yields.

### General procedure for the formation of *N*-(4-dialkylamino-6-oxo-1,2-diaryl-1,6-dihydro-pyrimidinin-5-yl)-*N*-prop-2-ynyl-benzenesulfonamide (3a–h)

To a well-stirred solution of *N*-(4-dialkylamino-6-oxo-1,2-diaryl-1,6-dihydro-pyrimidinin-5-yl)-4-methyl-benzenesulfonamide (2a–h) (1 g, 1.870–2.170 mmoles) in dry CHCl_3_ (30 mL) at 0 °C, was added, a solid sodium hydride (1.2 eq.) in small increments. The reaction was initially stirred for fifteen minutes and then the propargyl bromide (1.2 eq.) was added dropwise. The advancement of the reaction was checked by tlc. At the end of the reaction (5 hours stirring), a usual workup was carried out using ethyl acetate and water. The organic layers were combined, dried over sodium sulfate, and concentrated to obtain the crude product. The impure crude product was loaded into the column and purified by using a solution of ethyl acetate and hexane (1 : 9) as an eluent. The crude product was further purified using 10% dichloromethane in diethyl ether to obtain pure *N*-(4-dialkylamino-6-oxo-1,2-diaryl-1,6-dihydro-pyrimidinin-5-yl)-*N*-prop-2-ynyl-benzenesulfonamide (3a–h) in good yields.

#### *N*-(4-(diethylamino)-6-oxo-1,2-diphenyl-1,6-dihydropyrimidin-5-yl)-4-methyl-*N*-(prop-2-yn-1-yl)benzenesulfonamide (3a)

(1 g, 2.05 mmoles of 2a); yield-92%; white solid; ^1^H NMR (400 MHz, CDCl_3_): *δ* 7.71 (d, *J* = 8.3 Hz, 2H), 7.18–7.26 (m, 10H), 6.84 (dd, *J* = 7.3, 2.2 Hz, 2H), 4.69 (dd, *J* = 17.2, 2.6 Hz, 1H), 4.58 (dd, *J* = 17.2, 2.6 Hz, 1H), 4.13 (m, *J* = 14.1, 7.1 Hz, 2H), 3.82 (m, *J* = 14.1, 7.1 Hz, 2H), 2.42 (s, 3H), 2.31 (t, *J* = 2.5 Hz, 1H), 1.39 (t, *J* = 7.0 Hz, 6H). 13C NMR (101 MHz, CDCl3): *δ* 161.05, 158.61, 154.84, 143.39, 137.19, 135.72, 134.95, 129.65, 129.23, 129.08, 128.83, 128.54, 128.33, 127.98, 127.80, 96.62, 79.51, 73.25, 43.69, 39.74, 21.62, 13.80. HRMS (ESI + TOF) calcd for C_30_H_31_N_4_O_3_S^+^ (MH^+^): 527.2111, found: 527.2115.

#### General procedure for the synthesis of hexahydro-pteridines (4a–k)

To a solution of pyrimidinones, 3a–h (500 mg, 0.870–1.000 mmoles) in dry dichloromethane (20 mL) was added bromine or iodine (3 eq.) in small amounts at room temperature. The advancement of the reaction was checked by tlc. At the end of the reaction, (20 minutes stirring) the mixture was first quenched with an aqueous solution of sodium thiosulphate, and then workup was carried out using dichloromethane and brine solution. The filtrate was dried over sodium sulfate and concentrated to get the crude product. The crude product was purified using a solution of 10% dichloromethane in diethyl ether to get a pure compound, 4a–k in good yields.

#### (*E*)-8,8-Diethyl-7-(iodomethylene)-4-oxo-2,3-diphenyl-5-tosyl-3,4,5,6,7,8-hexahydropteridin-8-ium, iodide (4a)

(500 mg, 0.95 mmol of 3a); (680 mg recovered, yield-89%); white solid; ^1^H NMR (400 MHz, CDCl_3_): *δ* 7.63 (d, *J* = 8.5 Hz, 2H), 7.39–7.32 (m, 6H), 7.25–7.20 (m, 6H), 5.43 (d, *J* = 1.8 Hz, 1H), 4.99 (d, *J* = 17.3 Hz, 1H), 4.79 (dd, *J* = 17.1, 2.1 Hz, 1H), 4.51 (m, *J* = 14.7, 7.4 Hz, 1H), 4.16 (m, *J* = 13.8, 7.2 Hz, 1H), 3.94 (m, *J* = 14.6, 7.4 Hz, 1H), 3.78 (m, *J* = 14.6, 7.1 Hz, 1H), 2.49 (s, 3H), 1.56 (t, *J* = 7.4 Hz, 3H), 1.42 (t, *J* = 7.4 Hz, 3H). 13C NMR (101 MHz, CDCl_3_): *δ* 157.56, 155.46, 153.81, 146.41, 145.19, 134.10, 132.06, 131.94, 131.29, 130.29, 129.89, 129.78, 128.77, 128.29, 98.53, 64.57, 46.35, 21.99, 13.09; HRMS (ESI + TOF) calcd for C_30_H_30_IN_4_O_3_S^+^ (M^+^): 653.1078, found: 653.1107.

## Conflicts of interest

Authors declare no conflict of interest.

## Supplementary Material

RA-013-D3RA05651A-s001

## References

[cit1] Burger A. , Medicinal Chemistry, New York (NY), 1970, vol. 72, p. 719

[cit2] Delgado J. and Remers W. W., Wilson and Gisvold's Textbook of Organic Medicinal and Pharmaceutical Chemistry, ed. Lippinicott Williams and Wilkins, Philadelphia, PA, USA, 1998, p. 185

[cit3] Milstien S. , Kapatos G., Levine R. A. and Shane B., Chemistry and Biology of Pteridines and Folates: Proceedings of the 12th International Symposium on Pteridines and Folates, National Institutes of Health, Springer Science & Business Media, Bethesda, Maryland, 2012

[cit4] Sasada T., Kobayashi F., Sakai N., Konakahara T. (2009). Org. Lett..

[cit5] Dörrstein J., Scholz R., Schwarz D., Schieder D., Sieber V., Walther F., Zollfrank C. (2018). Compos. Struct..

[cit6] Leurs U., Lajkó E., Mező G., Orbán E., Öhlschläger P., Marquardt A., Kőhidai L., Manea M. (2012). Eur. J. Med. Chem..

[cit7] Li M.-H., Choi S. K., Thomas T. P., Desai A., Lee K.-H., Kotlyar A., Holl M. M. B., Baker Jr. J. R. (2012). Eur. J. Med. Chem..

[cit8] Rosowsky A., Forsch R. A., Freisheim J. H., Moran R. G. (1989). J. Med. Chem..

[cit9] Rosowsky A., Galivan J., Beardsley G., Bader H., O'Connor B. (1992). Cancer Res..

[cit10] Zhang Z., Wu J., Ran F., Guo Y., Tian R., Zhou S., Wang X., Liu Z., Zhang L., Cui J. (2009). Eur. J. Med. Chem..

[cit11] Fredi G., Dorigato A. (2021). Adv. Ind. Eng. Polym. Res..

[cit12] Lee C.-H., Jiang M., Cowart M., Gfesser G., Perner R., Kim K. H., Gu Y. G., Williams M., Jarvis M. F., Kowaluk E. A. (2001). J. Med. Chem..

[cit13] Enzinger C., Wirleitner B., Spöttl N., Böck G., Fuchs D., Baier-Bitterlich G. (2002). Neurochem. Int..

[cit14] Voet D. and Voet J., Biochemistry, John Wiley & Sons, Hoboken, NJ, 3rd edn, 2004, pp. 909–984

[cit15] Ding Y., Girardet J.-L., Smith K. L., Larson G., Prigaro B., Lai V. C., Zhong W., Wu J. Z. (2005). Bioorg. Med. Chem. Lett..

[cit16] Raboisson P., Lenz O., Lin T.-I., Surleraux D., Chakravarty S., Scholliers A., Vermeiren K., Delouvroy F., Verbinnen T., Simmen K. (2007). Bioorg. Med. Chem. Lett..

[cit17] Iwamura H., Masuda N., Koshimizu K., Matsubara S. (1980). Plant Sci. Lett..

[cit18] Katilius E., Katiliene Z., Woodbury N. W. (2006). Anal. Chem..

[cit19] Palanki M. S., Dneprovskaia E., Doukas J., Fine R. M., Hood J., Kang X., Lohse D., Martin M., Noronha G., Soll R. M. (2007). J. Med. Chem..

[cit20] Stanley R. J., Hou Z., Yang A., Hawkins M. E. (2005). J. Phys. Chem. B.

[cit21] Wenska G., Skalski B., Tomska-Foralewska I., Paszyc S. (2001). Helv. Chim. Acta.

[cit22] Hahn F. E., Jahnke M. C. (2008). Angew. Chem., Int. Ed..

[cit23] Brown D. J. , Fused Pyrimidines, John Wiley & Sons, 2009, Part 3: Pteridines, vol. 24

[cit24] Koller M. , in Handbook of Microalgae-Based Processes and Products, ed. E. Jacob-Lopes, M. M. Maroneze, M. I. Queiroz and L. Q. Zepka, Academic Press, 2020, pp. 597–645, 10.1016/B978-0-12-818536-0.00022-1

[cit25] Albert A. (1952). Q. Rev., Chem. Soc..

[cit26] Ashry E. S. H. E., Youssif S., Ahwany M. E., Sanan M. E. (2005). J. Chem. Res..

[cit27] Goswami S., Maity A. C. (2007). Chem. Lett..

[cit28] Mamedov V. A., Zhukova N. A., Gubaidullin A. T., Syakaev V. V., Kadyrova M. S., Beschastnova T. Y. N., Bazanova O. B., Rizvanov I. D. K., Latypov S. K. (2018). J. Org. Chem..

[cit29] Marchal A., Melguizo M., Nogueras M., Sanchez A., Low J. N. (2002). Synlett.

[cit30] Carmona-Martínez V., Ruiz-Alcaraz A. J., Vera M., Guirado A., Martínez-Esparza M., García-Peñarrubia P. (2019). Med. Res. Rev..

[cit31] Naikoo R. A., Kumar R., Singh P., Bhargava G. (2021). Synth. Commun..

[cit32] Naikoo R. A., Kumar R., Kumar V., Bhargava G. (2021). Synth. Commun..

[cit33] Sharma R., Mohan C. (2017). J. Heterocycl. Chem..

[cit34] Kuila B., Kumar Y., Mahajan D., Kumar K., Singh P., Bhargava G. (2016). RSC Adv..

[cit35] Kumar Y., Kuila B., Mahajan D., Singh P., Mohapatra B., Bhargava G. (2014). Tetrahedron Lett..

[cit36] Shelke G. M., Rao V. K., Jha M., Cameron T. S., Kumar A. (2015). Synlett.

[cit37] Sharma R., Gawande D. Y., Mohan C., Goel R. K. (2016). Med. Chem. Res..

