# Supplementary material for: Highly regioselective 6-exo-dig iodo/bromo cyclizations of functionalized 5-amino propargyl pyrimidinones: an efficient synthesis of functionalized pteridines
Source: RSC Adv. 2023 Oct 31;13(45):31891–6. doi: 10.1039/d3ra05651a (PMC10616752; doi:10.1039/d3ra05651a)
Supplement: RA-013-D3RA05651A-s001 [file RA-013-D3RA05651A-s001.pdf]

Electronic Supplementary Material (ESI) for RSC Advances.
This journal is © The Royal Society of Chemistry 2023

Supporting information

Highly Regioselective 6-*exo-dig* Iodo/Bromo Cyclizations of functionalized 5-amino propargyl Pyrimidinones: An Efficient Synthesis of Functionalized Pteridines

Rayees Ahmad Naikoo,[a] Rupesh Kumar,[a] Rashmi Sharma,[a*] Dinesh Mahajan,[b] Gaurav Bhargava[a*]

[a]*Department of Chemical Sciences, I. K. Gujral Punjab Technical University, Kapurthala, Punjab 144603, India*
[b]*Translational Health Science and Technology Institute, Faridabad, Haryana*
E-mail: gaurav@ptu.ac.in, rsharma082@gmail.com

EXPERIMENTAL SECTION:

General Information

All reactions are carried out in round bottom flask and reaction mixture was monitored by thin-layer chromatography (TLC). TLC pre-coated silica gel 60 F254 (20 × 20 cm). TLC plates are visualized by exposing UV light. Organic solvents are evaporated on rotary evaporator and the compounds are purified on flash Column chromatography (230-400 mesh size). Mass spectra are obtained using an Agilent 6540 accurate mass Q-TOF LC/MS (135 eV) spectrometer, using electrospray ionization (ESI). ^{1}H NMR spectra are recorded on 400 and 300 MHz NMR instruments. Chemical data for protons are reported in parts per million (ppm, scale) downfield from tetramethylsilane as referenced to the residual proton in the NMR solvent. All the NMR spectras are processed with MestReNova software. The coupling constant *(J*) are in Hz. ESI-MS and HRMS spectra are recorded on LC-Q-TOF machines.

General procedure for the formation of *N*-(4-dialkylamino-6-oxo-1,2-diaryl-1,6-dihydro-pyrimidinin-5-yl)-4-methyl-benzenesulfonamide (2a-h):

To a solution of 5-amino pyrimidinones **1a-h** (2g, 1.950 – 2.550 mmoles) and triethylamine (3 *eq.*, 5.85 – 7.65 mmoles) in dry $CHCl_3$ (50 mL) at 0 °C, was added dropwise a solution of *p*-TsCl (2.0 *eq.*, 3.90 – 5.10 mmoles) mixed in dry chloroform. The advancement of the reaction was checked by tlc. At the end of the reaction (overnight stirring), a usual workup was carried out using water and chloroform. The organic layers were combined, dried over sodium sulfate, and concentrated

to get the crude product. The impure crude product was loaded into the column and purified by using ethyl acetate and hexane (2:8) as an eluent. The crude compounds were further purified using a mixture of 10% dichloromethane in diethyl ether to obtain N-(4-dialkylamino-6-oxo-1,2-diaryl-1,6-dihydro-pyrimidinin-5-yl)-4-methyl-benzenesulfonamide **(2a-h)** as pure compounds in good yields.

General procedure for the formation of *N*-(4-dialkylamino-6-oxo-1,2-diaryl-1,6-dihydro-pyrimidinin-5-yl)-N-prop-2-ynyl--benzenesulfonamide (3a-h):

To a well-stirred solution of *N*-(4-dialkylamino-6-oxo-1,2-diaryl-1,6-dihydro-pyrimidinin-5-yl)-4-methyl-benzenesulfonamide **(2a-h)** (1g, 1.870 – 2.170 mmoles) in dry $CHCl_3$ (30 mL) at 0 °C, was added, a solid sodium hydride (1.2 *eq.* 2.244 – 2.604 mmoles) in small increments. The reaction was initially stirred for fifteen minutes and then the propargyl bromide (1.2 *eq.* 2.244 – 2.604 mmoles) was added dropwise. The advancement of the reaction was checked by tlc. At the end of the reaction (5 hours stirring), a usual workup was carried out using ethyl acetate and water. The organic layers were combined, dried over sodium sulfate, and concentrated to obtain the crude product. The impure crude product was loaded into the column and purified by using a solution of ethyl acetate and hexane (1:9) as an eluent. The crude product was further purified using 10% dichloromethane in diethyl ether to obtain pure N-(4-dialkylamino-6-oxo-1,2-diaryl-1,6-dihydro-pyrimidinin-5-yl)-N-prop-2-ynyl--benzenesulfonamide **(3a-h)** in good yields.

N-(4-(diethylamino)-6-oxo-1,2-diphenyl-1,6-dihydropyrimidin-5-yl)-4-methyl-N-(prop-2-yn-1-yl)benzenesulfonamide **(3a):**

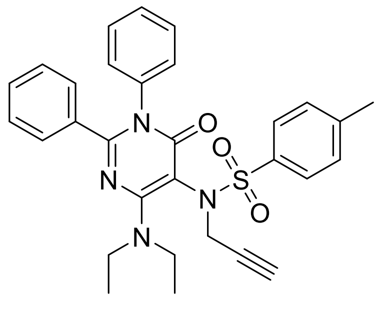

(1g, 2.05 mmoles of 2a); Yield-92%; White Solid; ^{1}H NMR (400 MHz, $CDCl_3$): δ 7.71 (d, J = 8.3 Hz, 2H), 7.18 – 7.26 (m, 10H), 6.84 (dd, J = 7.3, 2.2 Hz, 2H), 4.69 (dd, J = 17.2, 2.6 Hz, 1H), 4.58 (dd, J = 17.2, 2.6 Hz, 1H), 4.13 (m, J = 14.1, 7.1 Hz, 2H), 3.82 (m, J = 14.1, 7.1 Hz, 2H), 2.42 (s, 3H), 2.31 (t, J = 2.5 Hz, 1H), 1.39 (t, J = 7.0 Hz, 6H). ^{13}C NMR (101 MHz, $CDCl_3$): δ 161.05, 158.61, 154.84, 143.39, 137.19, 135.72, 134.95, 129.65, 129.23, 129.08, 128.83, 128.54, 128.33, 127.98, 127.80, 96.62, 79.51, 73.25, 43.69, 39.74, 21.62, 13.80. HRMS (ESI+TOF) calcd. for $C_{30}H_{31}N_4O_3S^+$ (MH^+): 527.2111, found: 527.2115

***N-(4-(diethylamino)-6-oxo-2-phenyl-1-(p-tolyl)-1,6-dihydropyrimidin-5-yl)-4-methyl-N-(prop-2-yn-1-yl)benzenesulfonamide* (3b)**

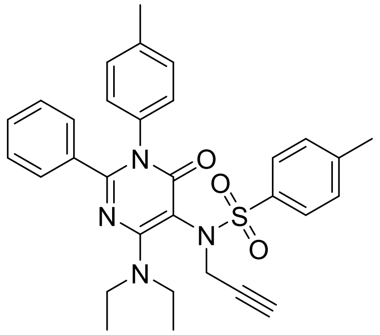

(1g, 1.99 mmoles of 2a); Yield-91%; Yellow Solid; ^{1}H NMR (400 MHz, $CDCl_3$): δ 7.60 (d, J = 8.3 Hz, 2H), 7.12 – 7.20 (m, 7H), 6.90 (d, J = 7.3 Hz, 2H), 6.59 (d, J = 8.4 Hz, 2H), 4.58 (dd, J = 17.3, 2.5 Hz, 1H), 4.43 (dd, J = 17.3, 2.6 Hz, 1H), 3.99 (m, J = 14.1, 7.0 Hz, 2H), 3.72 (m, J = 14.1, 7.0 Hz, 2H), 2.34 (s, 3H), 2.25 (t, J = 2.5 Hz, 1H), 2.16 (s, 3H), 1.28 (t, J = 7.0 Hz, 6H). ^{13}C NMR (101 MHz, $CDCl_3$): δ 161.18, 158.59, 154.91, 143.33, 137.89, 135.73, 135.07, 134.49, 129.57, 129.26, 129.21, 129.05, 128.47, 128.35, 127.78, 96.65, 79.58, 73.18, 43.65, 39.72, 21.62, 21.08, 13.79. HRMS (ESI+TOF) calcd. for $C_{31}H_{33}N_4O_3S^+$ (MH^+): 541.2268, found: 541.2281

***N-(4-(diethylamino)-6-oxo-2-phenyl-1-(o-tolyl)-1,6-dihydropyrimidin-5-yl)-4-methyl-N-(prop-2-yn-1-yl)benzenesulfonamide,* (3c)**

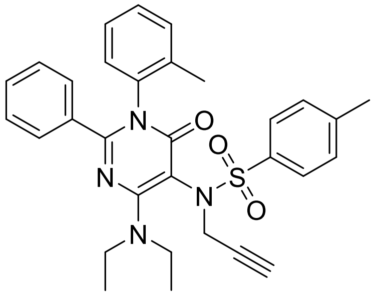

(1g, 1.99 mmoles of 2a); Yield-90%; ^{1}H NMR (400 MHz, $CDCl_3$): δ 7.60 (dd, J = 15.4, 8.3 Hz, 2H), 7.18 – 6.95 (m, 10H), 6.66 (dd, J = 31.2, 7.7 Hz, 1H), 4.55 (dd, J = 8.4, 2.6 Hz, 1H), 4.52 (dd, J = 3.3, 2.7 Hz, 1H), 4.11 – 3.95 (m, 2H), 3.81 – 3.61 (m, 2H), 2.28 (s, 3H), 2.21 (t, J = 2.6 Hz, 1H), 1.89 (s, 3H), 1.28 (t, J = 7.0 Hz, 6H). ^{13}C NMR (101 MHz, $CDCl_3$): δ 160.79, 158.95, 155.15, 146.04, 144.73, 143.43, 143.30, 131.14, 129.87, 129.49, 129.25, 129.07, 128.88, 128.71, 127.82, 97.02, 79.09, 73.57, 43.64, 39.46, 21.61, 17.62, 13.74. HRMS (ESI+TOF) calcd. for $C_{31}H_{33}N_4O_3S^+$ (MH^+): 541.2273, found: 541.2275

***N-(4-(dimethylamino)-6-oxo-1,2-diphenyl-1,6-dihydropyrimidin-5-yl)-4-methyl-N-(prop-2-yn-1-yl)benzenesulfonamide,* (3d)**

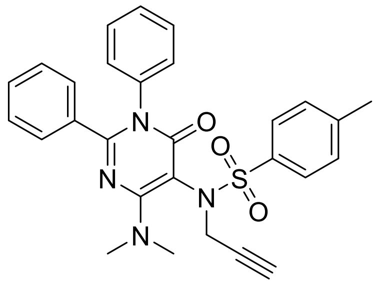

1g, 2.17 mmoles of 2a); Yield-92%; White Solid; ^{1}H NMR (400 MHz, $CDCl_3$): δ 7.67 (d, J = 8.3 Hz, 2H), 7.22 (m, 12H), 6.80 (dd, J = 7.2, 2.5 Hz, 2H), 4.63 (dd, J = 7.7, 2.6 Hz, 2H), 3.47 (s, 6H), 2.39 (s, 3H), 2.34 (t, J = 2.6 Hz, 1H). ^{13}C NMR (101 MHz, $CDCl_3$): δ 160.83, 159.10, 155.21, 143.39, 137.20, 135.93, 134.91, 129.68, 129.24, 129.11, 128.85, 128.57, 128.15, 128.03, 127.79, 96.29, 79.06, 74.01, 40.82, 39.19, 21.59. HRMS (ESI+TOF) calcd. for $C_{28}H_{27}N_4O_3S^+$ (MH^+): 499.1804, found: 499.1801

N-(4-(dipropylamino)-6-oxo-1,2-diphenyl-1,6-dihydropyrimidin-5-yl)-4-methyl-N-(prop-2-yn-1-yl)benzenesulfonamide, **(3e)**

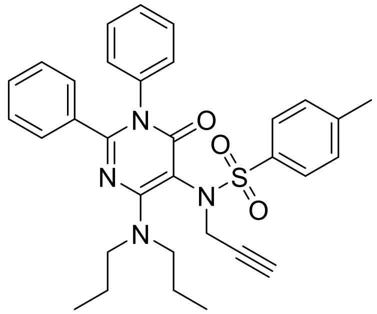

(1g, 1.93 mmoles of 2a); Yield-77%; ^{1}H NMR (400 MHz, $CDCl_3$): δ 7.59 (d, J = 8.3 Hz, 2H), 7.22 – 7.16 (m, 2H), 7.13 - 7.07 (m, 8H), 6.72 (dd, J = 7.3, 2.3 Hz, 2H), 4.58 (dd, J = 17.1, 2.6 Hz, 1H), 4.45 (dd, J = 17.1, 2.6 Hz, 1H), 4.04 - 3.97 (m, 2H), 3.54 – 3.46 (m, 2H), 2.30 (s, 3H), 2.22 (t, J = 2.6 Hz, 1H), 1.81 – 1.65 (m, 4H), 0.91 (t, J = 7.4 Hz, 6H). ^{13}C NMR (101 MHz, $CDCl_3$): δ 161.14, 158.72, 154.68, 143.36, 137.25, 135.69, 134.97, 129.62, 129.23, 129.04, 128.85, 128.54, 128.36, 127.97, 127.77, 96.85, 79.33, 73.45, 51.22, 39.73, 21.93, 21.63, 11.51. HRMS (ESI+TOF) calcd. for $C_{32}H_{35}N_4O_3S^+$ (MH^+): 555.2430, found: 555.2419

4-methyl-N-(4-(methyl(phenyl)amino)-6-oxo-1,2-diphenyl-1,6-dihydropyrimidin-5-yl)-N-(prop-2-yn-1-yl)benzenesulfonamide **(3f):**

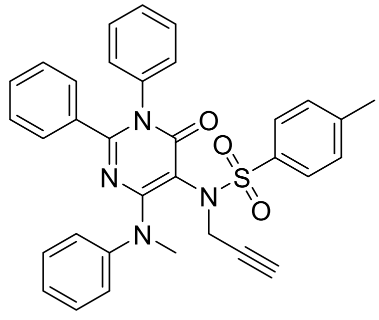

(1g, 1.92 mmoles of 2a); Yield-85%; White Solid; 1H NMR (400 MHz, CDCl3): δ 7.68 (d, J = 8.2 Hz, 2H), 7.45 – 7.42 (m, 4H), 7.28 (m, 1H), 7.21 – 7.23 (m, 5H), 7.18 – 7.08 (m, 5H), 6.90 – 6.87 (m, 2H), 4.40 (dd, J = 16.5, 2.6 Hz, 1H), 4.29 (dd, J = 16.5, 2.6 Hz, 1H), 3.82 (s, 3H), 2.38 (s, 3H), 2.32 (t, J = 2.5 Hz, 1H). 13C NMR (101 MHz, CDCl3): δ 161.31, 159.67, 155.25, 147.00, 143.44, 137.20, 135.80, 134.39, 129.78, 129.48, 129.13, 128.97, 128.78, 128.71, 128.18, 128.12, 127.68, 126.91, 126.07, 99.59, 78.65,

73.75, 42.02, 39.20, 21.59. HRMS (ESI+TOF) calcd. for C33H29N4O3S+ (MH+): 561.1955, found: 561.1962

N-(4-(ethyl(phenyl)amino)-6-oxo-1,2-diphenyl-1,6-dihydropyrimidin-5-yl)-4-methyl-N-(prop-2-yn-1-yl)benzenesulfonamide **(3g):**

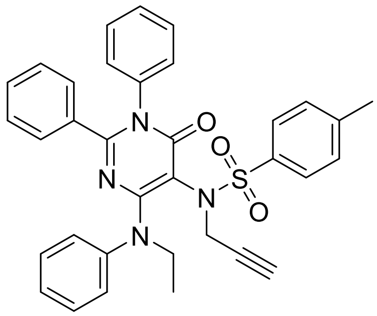

(1g, 1.87 mmoles of 2a); Yield-82%; Yellow Solid; ^{1}H NMR (400 MHz, $CDCl_3$): δ 7.63 (d, J = 8.3 Hz, 2H), 7.50 – 7.42 (m, 4H), 7.32 – 7.27 (m, 6H), 7.19 - 7.22 (m, J = 5.5 Hz, 5H), 6.89 (dd, J = 7.6, 1.9 Hz, 2H), 4.37 - 4.03 (dd, J = 8.2, 5.7 Hz, 1H), 4.00 – 3.97 (m, 1H), 3.76 (dd, J = 16.1, 2.6 Hz, 1H), 2.38 (s, 3H), 2.18 (t, J = 2.6 Hz, 1H), 1.31 (t, J = 7.0 Hz, 3H). ^{13}C NMR (101 MHz, $CDCl_3$): δ 159.60, 155.77, 144.12, 143.22, 137.26, 135.94, 134.76, 129.75, 129.58, 129.41, 128.99, 128.97, 128.81, 128.66, 128.16, 128.09, 127.98, 127.77, 126.48, 102.92, 78.54, 73.03, 48.37, 39.41, 21.56, 13.94. HRMS (ESI+TOF) calcd. for $C_{34}H_{31}N_4O_3S^+$ (MH^+): 575.2111, found: 575.2115

N-(4-(dimethylamino)-6-oxo-2-phenyl-1-(p-tolyl)-1,6-dihydropyrimidin-5-yl)-4-methyl-N-(prop-2-yn-1-yl)benzenesulfonamide **(3h):**

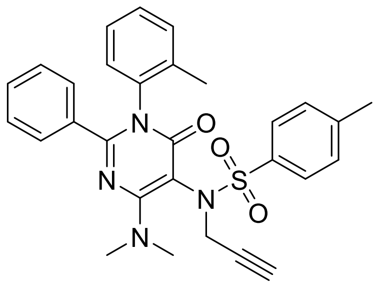

(1g, 2.11 mmoles of 2a); Yield-90%; Cream Solid; ^{1}H NMR (400 MHz, $CDCl_3$): δ 7.88 (d, J = 8.3 Hz, 2H), 7.69 (d, J = 8.3 Hz, 1H), 7.32 – 7.19 (m, 8H), 7.00 (d, J = 6.8 Hz, 1H), 6.69 (d, J = 8.5 Hz, 1H), 3.48 (s, 2H), 2.43 (s, 3H), 2.26 (s, 1H), 1.98 (s, 3H), 1.51 (s, 6H). ^{13}C NMR (101 MHz, $CDCl_3$): δ 155.10, 143.35, 143.28, 137.93, 137.56, 135.93, 135.01, 134.48, 129.62, 129.25, 129.23, 129.09, 128.16, 128.03, 127.77, 96.34, 79.15, 68.28, 53.37, 40.79, 27.00, 21.57. HRMS (ESI+TOF) calcd. for $C_{29}H_{29}N_4O_3S^+$ (MH^+): 513.1955, found: 513.1963

General procedure for the synthesis of hexahydro-pteridines (4a-k):

To a solution of pyrimidinones, **3a-h** (500 mg, 0.870 – 1.000 mmoles) in dry dichloromethane (20 mL) was added bromine or iodine (3 *eq.*, 2.61 – 3.00 mmoles) in small amounts at room temperature. The advancement of the reaction was checked by tlc. At the end of the reaction, (20 minutes stirring) the mixture was first quenched with an aqueous solution of sodium thiosulphate, and then work up was carried out using dichloromethane and brine solution. The filtrate was dried over sodium sulfate and concentrated to get the crude product. The crude product were purified using a solution of 10% dichloromethane in diethyl ether to get a pure compound, **4a-k** in good yields. The Impure compounds, **4a-k** were purified by using a solvent mixture of dichloromethane and diethyl ether (1:9) without performing any column chromatography.

(E)-8,8-diethyl-7-(iodomethylene)-4-oxo-2,3-diphenyl-5-tosyl-3,4,5,6,7,8-hexahydropteridin-8-ium, Iodide **(4a):**

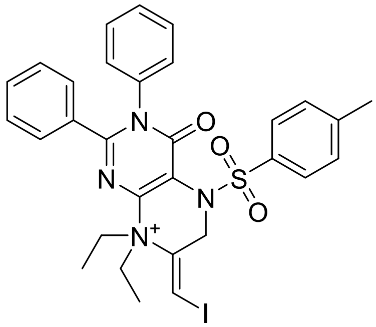

(500 mg, 0.95 mmol of 3a); (680 mg recovered, Yield-89%); White Solid; ^{1}H NMR (400 MHz, $CDCl_3$): δ 7.59 (d, J = 8.1 Hz, 2H), 7.36 – 7.16 (m, 12H), 5.39 (d, J = 1.7 Hz, 1H), 4.96 (dd, J = 17.0, 6.3 Hz, 1H), 4.77 (d, J = 16.9 Hz, 1H), 4.47 (m, J = 14.5, 7.2 Hz, 1H), 4.12 (m, J = 13.6, 7.1 Hz, 1H), 3.89 (m, J = 14.4, 7.2 Hz, 1H), 3.73 (m, J = 13.6, 7.1 Hz, 1H), 2.44 (s, 3H), 1.53 (t, J = 7.1 Hz, 3H), 1.38 (t, J = 7.1 Hz, 3H). ^{13}C NMR (101 MHz, $CDCl_3$): δ 157.56, 155.46, 153.81, 146.41, 145.19, 134.10, 132.06, 131.94, 131.29, 130.29, 129.89, 129.78, 128.77, 128.29, 98.53, 64.57, 46.35, 21.99, 13.09; HRMS (ESI+TOF) calcd. for $C_{30}H_{30}IN_4O_3S^+$ (M^+): 653.1078, found: 653.1107

(E)-8,8-diethyl-7-(iodomethylene)-4-oxo-2-phenyl-3-(p-tolyl)-5-tosyl-3,4,5,6,7,8-hexahydropteridin-8-ium, Iodide **(4b):**

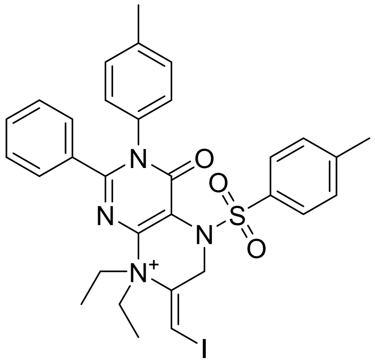

(500 mg, 0.93 mmol of 3a); (640 mg recovered, Yield-87%); Cream Solid; ^{1}H NMR (400 MHz, $CDCl_3$): δ 7.67 (d, J = 8.3 Hz, 2H), 7.47 – 7.44 (m, 2H), 7.39 – 7.37 (m, 2H), 7.29 – 7.27 (m, 5H),

7.06 (d, J = 8.3 Hz, 2H), 5.50 (d, J = 1.7 Hz, 1H), 5.07 (d, J = 17.1 Hz, 1H), 4.86 (dd, J = 17.0, 1.9 Hz, 1H), 4.60 – 4.51 (m, 1H), 4.23 (m, J = 13.6, 7.1 Hz, 1H), 3.96 (m, J = 14.5, 7.2 Hz, 1H), 3.81 (m, J = 13.5, 7.1 Hz, 1H), 2.54 (s, 3H), 2.29 (s, 3H), 1.62 (t, J = 7.2 Hz, 3H), 1.46 (t, J = 7.1 Hz, 3H). ^{13}C NMR (101 MHz, $CDCl_3$): δ 155.48, 153.98, 146.28, 145.27, 140.56, 132.04, 131.95, 131.35, 131.20, 129.92, 129.85, 129.69, 128.74, 128.67, 128.23, 98.47, 63.97, 46.30, 21.88, 21.24, 13.99. HRMS (ESI+TOF) calcd. for $C_{31}H_{32}IN_4O_3S^+$ (M^+): 667.1234, found: 667.1248

***(E)-8,8-diethyl-7-(iodomethylene)-4-oxo-2-phenyl-3-(o-tolyl)-5-tosyl-3,4,5,6,7,8-hexahydropteridin-8-ium, Iodide* (4c):**

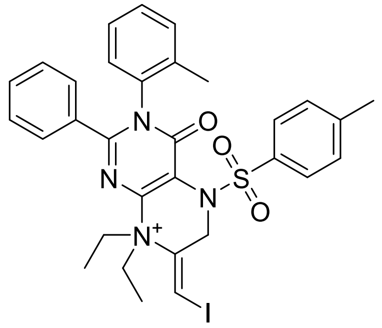

(500 mg, 0.93 mmol of 3a); (640 mg recovered, Yield-88%); Cream Solid; ^{1}H NMR (400 MHz, $CDCl_3$): δ 7.62 (d, J = 8.2 Hz, 2H), 7.33 – 7.15 (m, 9H), 7.07 (d, J = 6.9 Hz, 2H), 5.45 (d, J = 0.8 Hz, 1H), 4.93 (d, J = 16.9 Hz, 1H), 4.81 (dd, 17.2, 2.0 Hz, 1H), 4.48 (m, J = 14.4, 7.2 Hz, 1H), 4.16 (m, J = 13.6, 7.0 Hz, 1H), 4.00 (m, J = 14.4, 7.2 Hz, 1H), 3.73 (m, J = 13.6, 7.0 Hz, 1H), 2.41 (s, 3H), 1.96 (s, 3H), 1.55 (t, J = 7.2 Hz, 3H), 1.39 (t, J = 7.1 Hz, 3H). ^{13}C NMR (101 MHz): δ 158.08, 155.37, 153.50, 146.24, 145.39, 134.25, 133.71, 132.58, 131.42, 131.26, 130.67, 130.42, 129.33, 128.69, 128.24, 98.86, 65.23, 46.20, 21.95, 17.94, 13.02. HRMS (ESI+TOF) calcd. for $C_{31}H_{32}IN_4O_3S^+$ (M^+): 667.1234, found: 667.1240

***(E)-7-(iodomethylene)-8,8-dimethyl-4-oxo-2,3-diphenyl-5-tosyl-3,4,5,6,7,8-hexahydropteridin-8-ium, Iodide* (4d):**

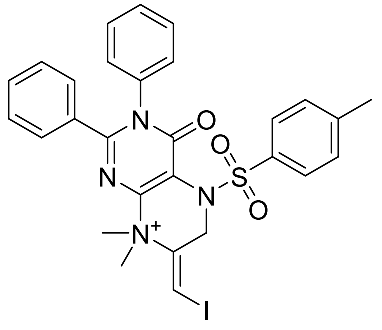

(500 mg, 1.00 mmol of 3a); (680 mg recovered, Yield-90%); Yellow Solid; ^{1}H NMR (400 MHz, $CDCl_3$): δ 7.78 (d, J = 8.4 Hz, 1H), 7.68 (d, J = 8.3 Hz, 2H), 7.46 – 7.43 (m, 2H), 7.39 – 7.37 (m, 2H), 7.25 - 7.27 (m, 5H), 7.20 (t, J = 4.0 Hz, 2H), 5.51 (d, J = 1.6 Hz, 1H), 5.01 (d, J = 16.9 Hz, 1H), 4.81 (dd, J = 16.9, 1.0 Hz, 1H), 3.80 (s, 3H), 3.56 (s, 3H), 2.53 (s, 3H).

^{13}C NMR (101 MHz, $CDCl_3$): δ 158.57, 155.44, 153.48, 146.23, 145.34, 134.09, 132.17, 131.72, 131.41, 130.29, 130.01, 129.70, 128.69, 128.60, 128.23, 128.06, 127.81, 98.75, 64.18, 42.09, 41.83, 21.90. HRMS (ESI+TOF) calcd. for $C_{28}H_{26}IN_4O_3S^+$ (M^+): 625.0765, found: 625.0778

***(E)-7-(iodomethylene)-4-oxo-2,3-diphenyl-8,8-dipropyl-5-tosyl-3,4,5,6,7,8-hexahydropteridin-8-ium, Iodide* (4e):**

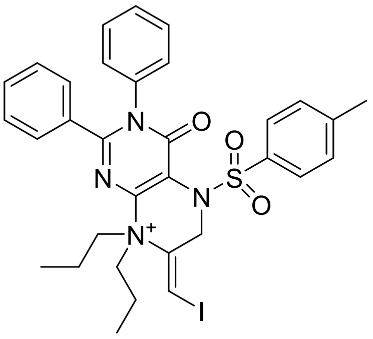

(500 mg, 0.90 mmol of 3a); (510 mg recovered, Yield-71%); White Solid; ^1H NMR (300MHz, $CDCl_3$): δ 7.65 (d, 8.2 Hz, 2H); 7.23 - 7.41 (m, 12H); 5.43 (d, 1H); 5.02 (d, 16.3 Hz, 1H); 4.77 (d, 16.5 Hz, 1H); 3.50 (m, 1H); 4.30 (m, 1H,); 3.55 (m, 2H); 2.49 (s, 3H); 1.94 (m, 4H); 1.06 (m, 6H); ^{13}C NMR (101 MHz, $CDCl_3$): δ 157.20, 155.35, 153.88, 146.40, 145.14, 134.04, 131.99, 131.88, 131.15, 130.22, 129.79, 129.73, 128.71, 128.18, 98.46, 64.47, 54.22, 53.75, 45.95, 22.47, 21.89, 21.16, 11.50, 11.39; HRMS (ESI+TOF) calcd. for $C_{32}H_{34}IN_4O_3S^+$ (M^+): 681.1391, found: 681.1395

***(E)-7-(iodomethylene)-8-methyl-4-oxo-2,3,8-triphenyl-5-tosyl-3,4,5,6,7,8-hexahydropteridin-8-ium, Iodide* (4f):**

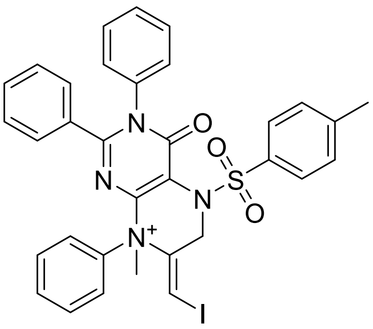

(500 mg, 0.89 mmol of 3a); (620 mg recovered, Yield-84%); Cream Solid; ^1H NMR (400 MHz, $CDCl_3$): δ 7.56 – 7.47 (m, 7H), 7.42 – 7.27 (m, 12H), 5.42 (d, J = 1.5 Hz, 1H), 4.36 (d, J = 17.2 Hz, 1H), 4.23 (dd, J = 17.2, 1.8 Hz, 1H), 3.90 (s, 3H), 2.46 (s, 3H); ^{13}C NMR (101 MHz, $CDCl_3$): δ 158.32, 156.81, 154.51, 145.98, 145.67, 143.00, 134.08, 132.92, 131.91, 131.39, 131.06, 130.50, 130.10, 129.76, 129.42, 128.88, 128.36, 128.29, 128.00, 127.34, 126.11, 99.71, 64.77, 44.45, 43.35, 21.87; HRMS (ESI+TOF) calcd. for $C_{33}H_{28}IN_4O_3S^+$ (M^+): 687.0921, found: 687.0938

***(E)-8-ethyl-7-(iodomethylene)-4-oxo-2,3,8-triphenyl-5-tosyl-3,4,5,6,7,8-hexahydropteridin-8-ium, Iodide* (4g):**

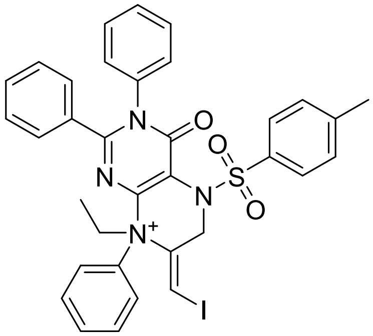

(500 mg, 0.87 mmol of 3a); (590 mg recovered, Yield-82%); Maroon Solid; ^1H NMR (400 MHz, $CDCl_3$): δ 7.56 – 7.49 (m, 7H),

7.41 – 7.28 (m, 12H), 5.41 (d, *J* = 1.6 Hz, 1H), 4.77 (m, *J* = 13.3, 7.1 Hz, 1H), 4.35 (d, *J* = 17.2 Hz, 1H), 4.17 (dd, *J* = 17.2, 1.9 Hz, 1H), 3.96 (m, *J* = 13.3, 7.1 Hz, 1H), 2.47 (s, 3H), 1.47 (t, *J* = 7.1 Hz, 3H). ^{13}C NMR (101 MHz, $CDCl_3$): δ 158.09, 156.81, 154.63, 153.63, 145.94, 145.65, 141.18, 134.08, 132.98, 132.00, 131.18, 130.46, 130.42, 129.97, 129.50, 129.35, 128.95, 128.38, 128.19, 127.34, 126.31, 99.55, 64.67, 51.08, 44.46, 21.88, 13.17; HRMS (ESI+TOF) calcd. for $C_{34}H_{30}IN_4O_3S^+$ (M^+): 701.1078, found: 701.1081

***(E)-7-(bromomethylene)-8,8-dimethyl-4-oxo-2-phenyl-3-(o-tolyl)-5-(p-tolyl)-3,4,5,6,7,8-hexahydropteridin-8-ium, bromide* (4h):**

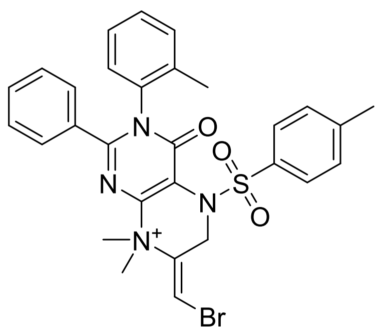

(500 mg, 0.98 mmol of 3a); (440 mg recovered, Yield-86%); Orange Solid; ^{1}H NMR (400 MHz, $CDCl_3$): δ 7.58 (d, *J* = 8.2 Hz, 2H), 7.47 (d, *J* = 7.9 Hz, 1H), 7.31 – 7.22 (m, 5H), 7.17 – 7.04 (m, 4H), 6.94 (d, *J* = 7.0 Hz, 1H), 5.41 (s, 1H), 4.95 (d, *J* = 17.0 Hz, 1H), 4.54 (d, *J* = 15.8 Hz, 1H), 3.70 (s, 3H), 3.47 (s, 3H), 2.39 (s, 3H), 1.81 (s, 3H); ^{13}C NMR (101 MHz, $CDCl_3$): δ 158.52, 155.60, 153.12, 146.21, 144.78, 134.32, 133.76, 132.59, 131.76, 131.42, 130.67, 130.45, 130.10, 129.46, 128.49, 128.24, 128.14, 98.13, 94.14, 53.68, 41.82, 41.75, 21.87, 17.89; HRMS (ESI+TOF) calcd. for $C_{29}H_{28}BrN_4O_3S^+$ (M^+): 591.1060, found: 591.1063

***(E)-7-(bromomethylene)-8,8-dimethyl-4-oxo-2,3-diphenyl-5-tosyl-3,4,5,6,7,8-hexahydropteridin-8-ium, bromide* (4i):**

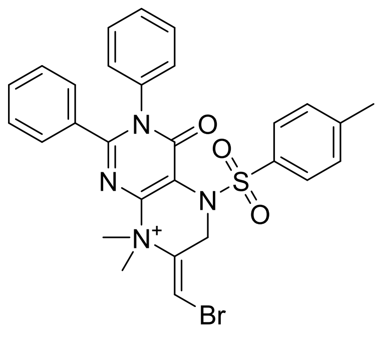

(500 mg, 1.00 mmol of 3a); (550 mg recovered, Yield-85%); White Solid; ^{1}H NMR (400 MHz, $CDCl_3$): δ 7.61 (d, *J* = 8.1 Hz, 2H), 7.35 (dd, *J* = 15.9, 7.7 Hz, 4H), 7.29 – 7.14 (m, 8H), 5.44 (s, 1H), 4.96 (d, *J* = 17.1 Hz, 1H), 4.55 (d, *J* = 17.1 Hz, 1H), 3.72 (s, 3H), 3.51 (s, 3H), 2.48 (s, 3H); ^{13}C NMR (101 MHz, $CDCl_3$): δ 158.09, 155.69, 153.48, 146.32, 144.64, 134.15, 132.19, 131.88, 131.31, 130.33, 130.25, 130.11, 129.80, 128.77, 128.58, 128.29, 97.90, 94.24, 42.80, 41.78, 41.72, 21.95; HRMS (ESI+TOF) calcd. for $C_{28}H_{26}BrN_4O_3S^+$ (M^+): 577.0904, found: 577.0907

(E)-7-(bromomethylene)-8,8-diethyl-4-oxo-2,3-diphenyl-5-tosyl-3,4,5,6,7,8-hexahydropteridin-8-ium, bromide **(4j):**

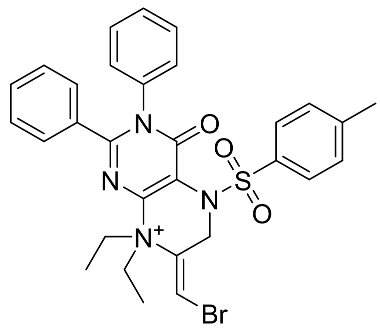

(500 mg, 0.95 mmol of 3a); (540 mg recovered, Yield-84%); Orange Solid; ^{1}H NMR (400 MHz, $CDCl_3$): δ 7.67 (d, J = 7.9 Hz, 2H), 7.44 – 7.28 (m, 12H), 5.46 (s, 1H), 5.06 (d, J = 16.2 Hz, 1H), 4.88 (d, J = 17.3 Hz, 1H), 4.59 (m, J = 13.5, 5.4 Hz, 1H), 4.26 (m, J = 13.7, 6.1 Hz, 1H), 3.95 (m, J = 13.9, 6.0 Hz, 1H), 3.79 (m, J = 12.8, 7.2 Hz, 1H), 2.53 (s, 3H), 1.59 (t, J = 6.7 Hz, 3H), 1.45 (t, J = 6.5 Hz, 3H). ^{13}C NMR (101 MHz, $CDCl_3$): δ 157.62, 155.44, 153.89, 146.37, 144.60, 134.19, 132.14, 131.23, 130.26, 129.97, 129.78, 128.71, 128.27, 116.53, 98.44, 94.33, 46.53, 46.37, 43.61, 21.96, 14.05, 13.11; HRMS (ESI+TOF) calcd. for $C_{30}H_{30}BrN_4O_3S^+$ (M^+): 605.1217, found: 605.1227

(E)-7-(bromomethylene)-8,8-diethyl-4-oxo-2-phenyl-3-(p-tolyl)-5-tosyl-3,4,5,6,7,8-hexahydropteridin-8-ium, bromide **(4k)**

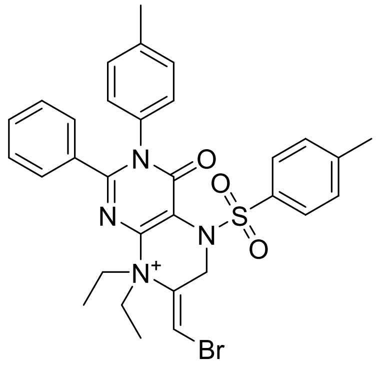

(500 mg, 0.93 mmol of 3a); (530 mg recovered, Yield-82%); Yellow Solid; ^{1}H NMR (400 MHz, $CDCl_3$): δ 7.67 (d, J = 8.3 Hz, 2H), 7.45 – 7.42 (m, 2H), 7.38 – 7.36 (m, 2H), 7.30 – 7.28 (m, 5H), 7.06 (d, J = 8.0 Hz, 2H), 5.46 (d, J = 1.4 Hz, 1H), 5.06 (d, J = 16.9 Hz, 1H), 4.92 (dd, J = 16.9, 1.9 Hz, 1H), 4.61 (m, J = 14.4, 7.2 Hz, 1H), 4.29 – 4.20 (m, 1H), 3.90 (m, J = 10.7, 5.4 Hz, 1H), 3.77 (m, J = 14.2, 7.1 Hz, 1H), 2.52 (s, 3H), 2.28 (s, 3H), 1.58 (t, J = 7.2 Hz, 3H), 1.45 (t, J = 7.1 Hz, 3H). ^{13}C NMR (101 MHz, $CDCl_3$): δ 155.42, 154.03, 146.16, 144.66, 140.41, 132.20, 132.11, 131.54, 131.05, 131.03, 129.81, 129.63, 128.66, 128.15, 94.04, 46.18, 21.84, 21.23, 13.95, 12.95. HRMS (ESI+TOF) calcd. for $C_{31}H_{32}BrN_4O_3S^+$ (M^+): 619.1373, found: 619.1376

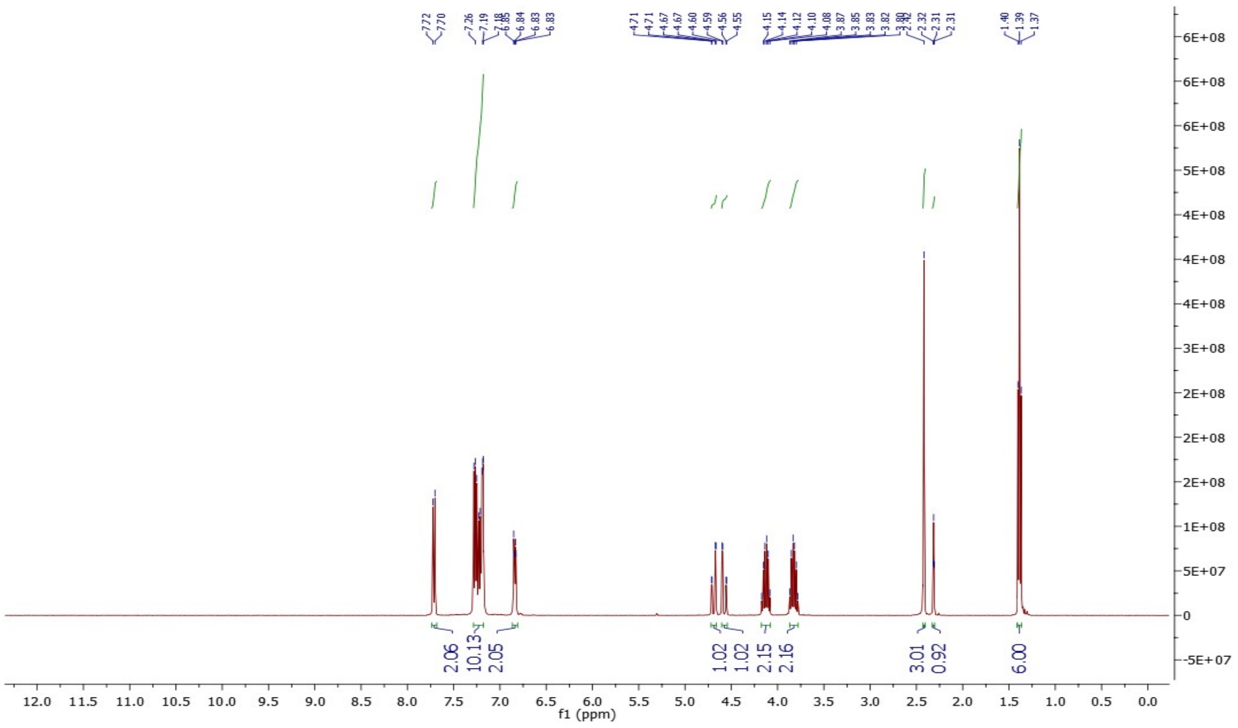

Figure 1. Proton NMR spectrum of 3a ($CDCl_3$).

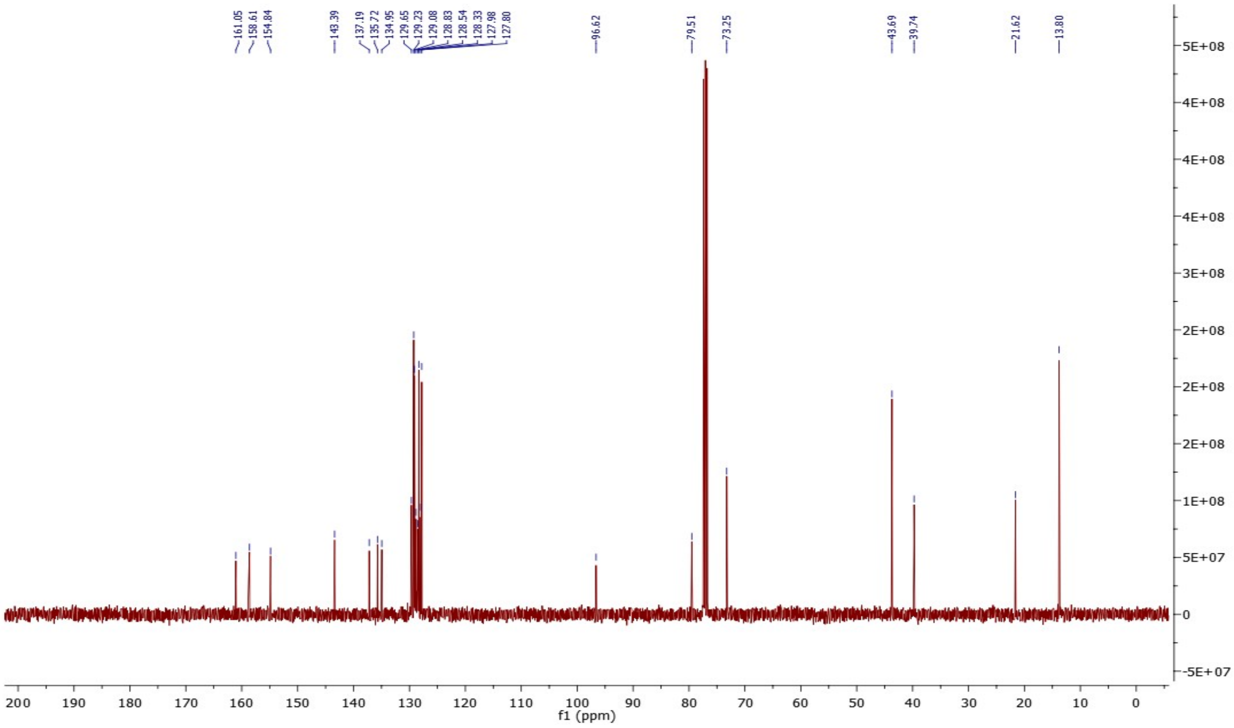

Figure 2. Carbon-13 NMR spectrum of 3a ($CDCl_3$).

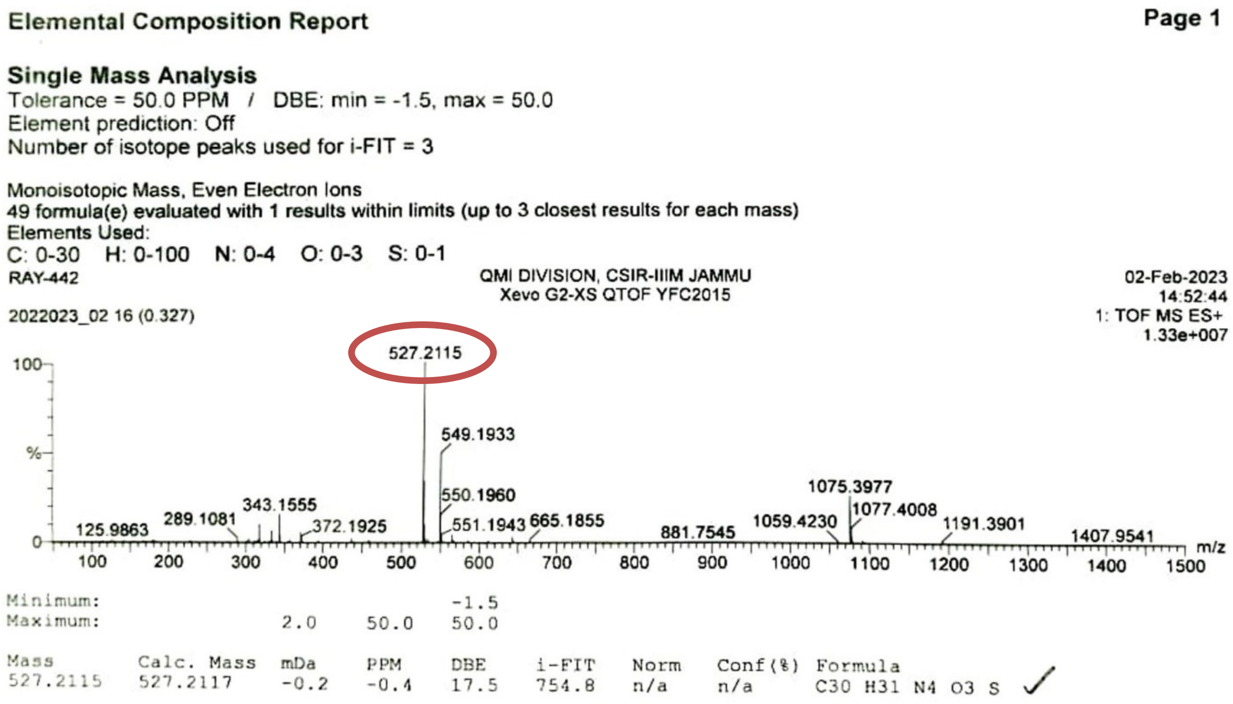

Figure 3. HRMS Spectrum of 3a

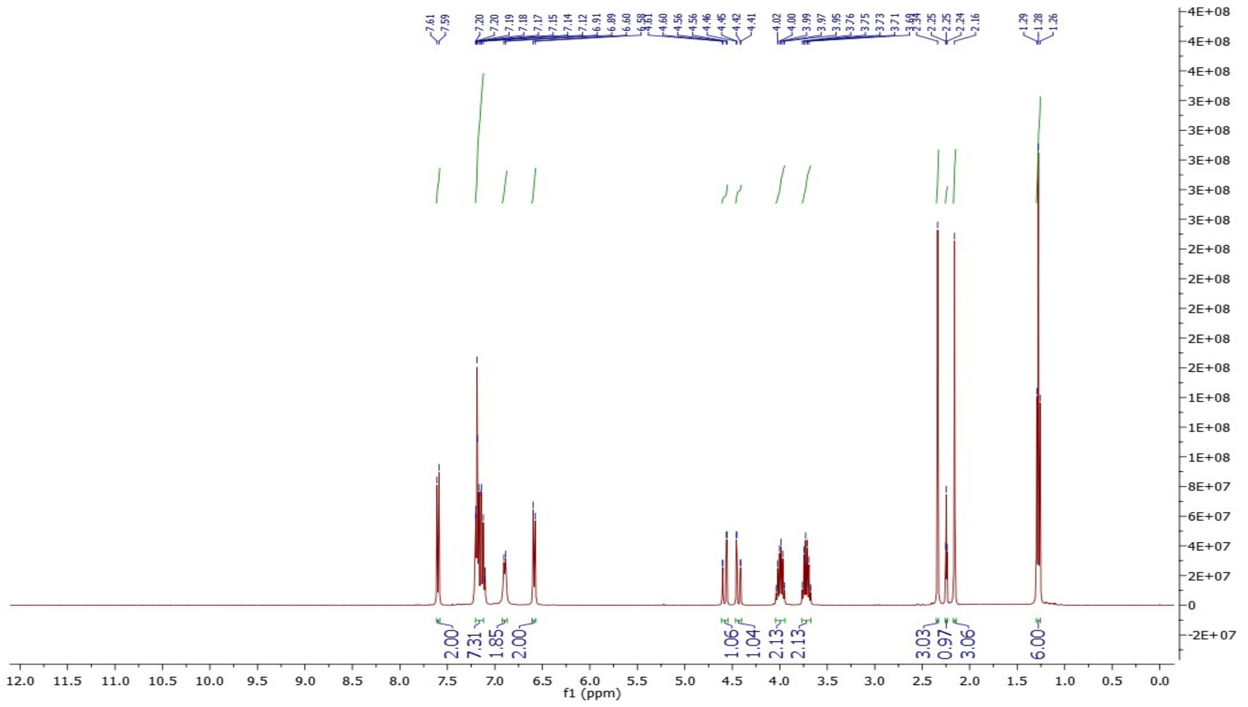

Figure 4. Proton NMR spectrum of 3b ($CDCl_3$).

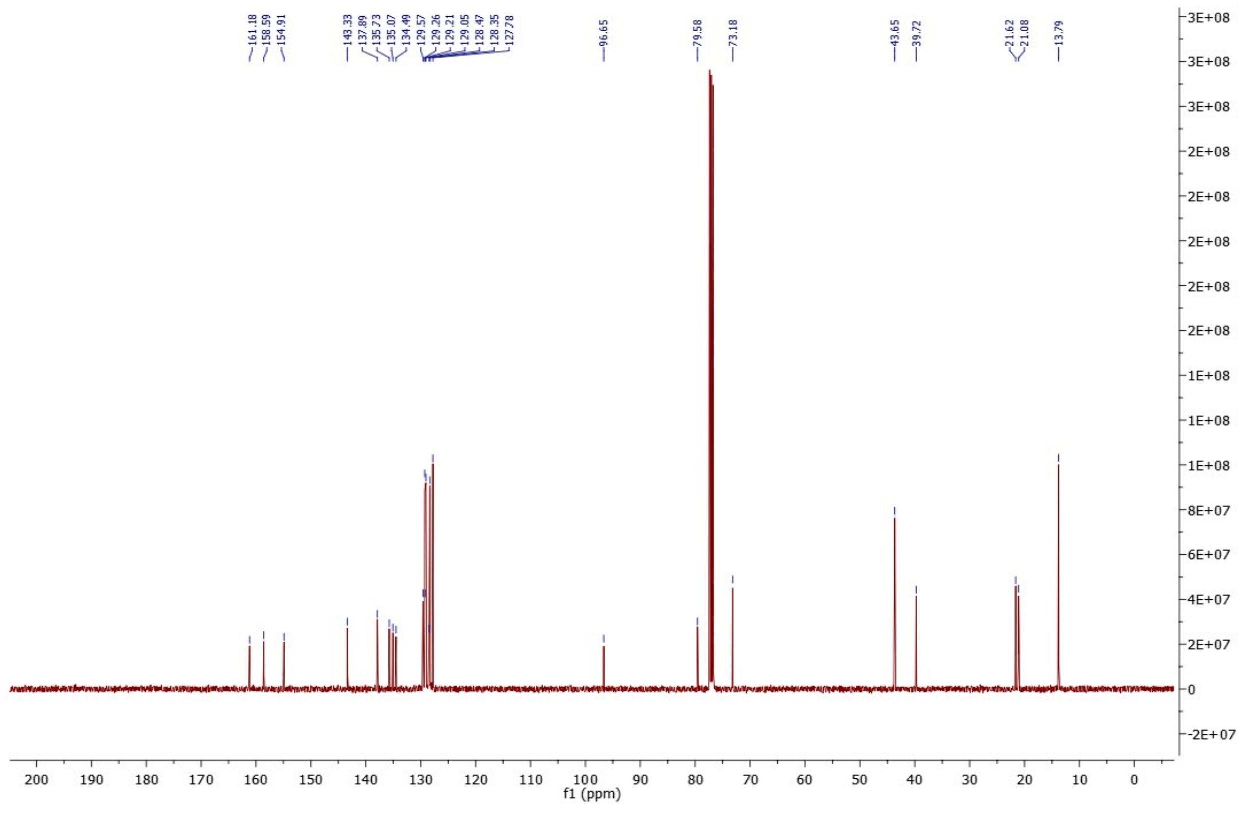

Figure 5. Carbon-13 NMR spectrum of 3b ($CDCl_3$).

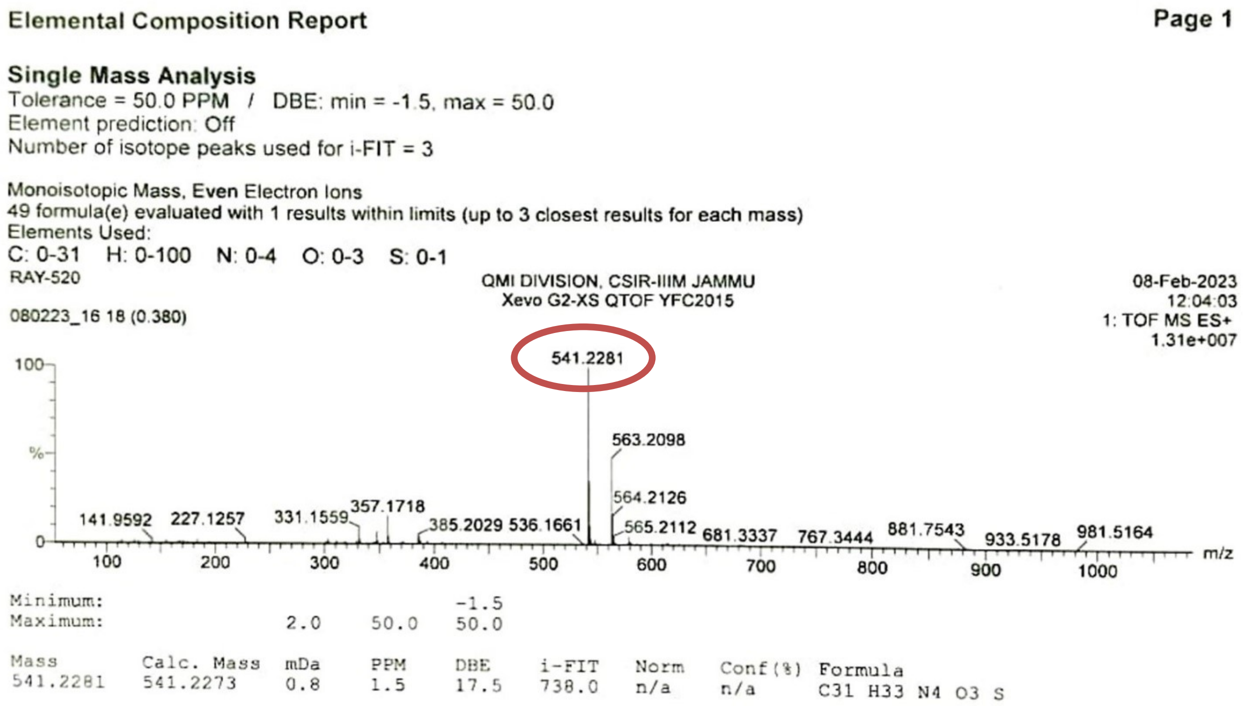

Figure 6. HRMS Spectrum of 3b

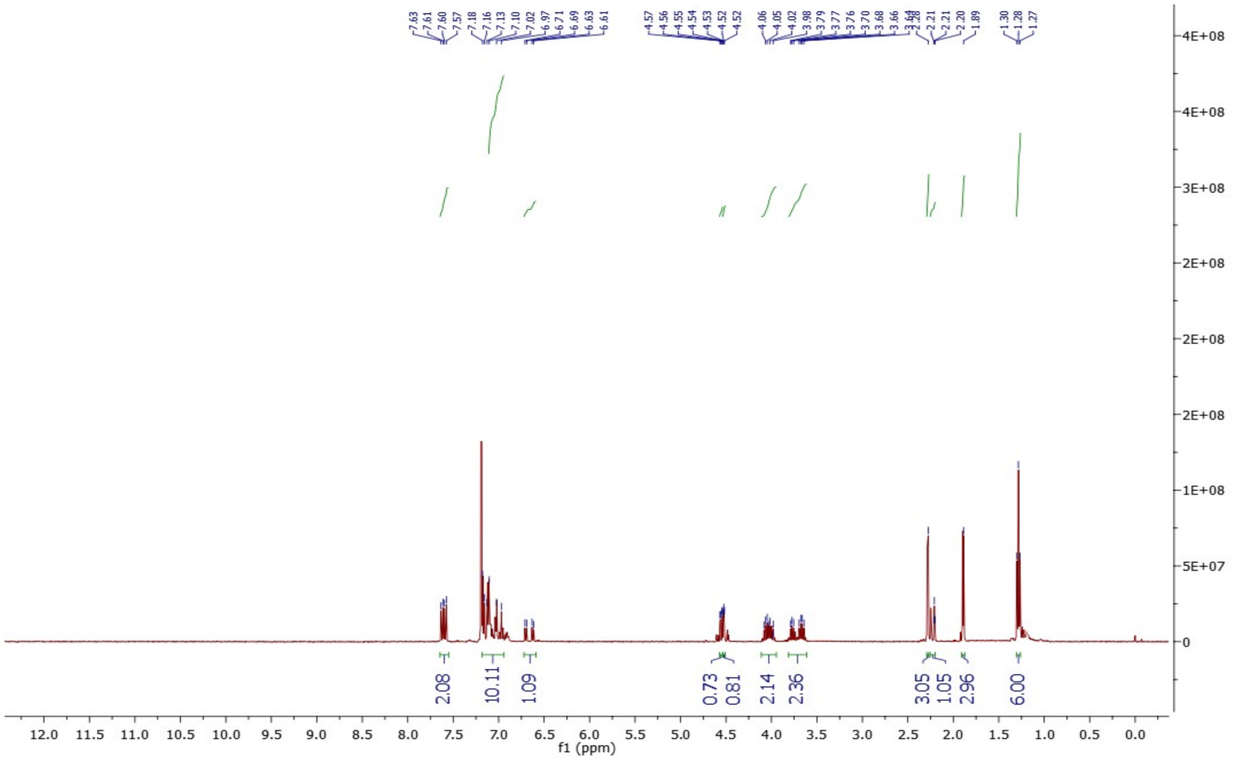

Figure 7. Proton NMR spectrum of 3c ($CDCl_3$).

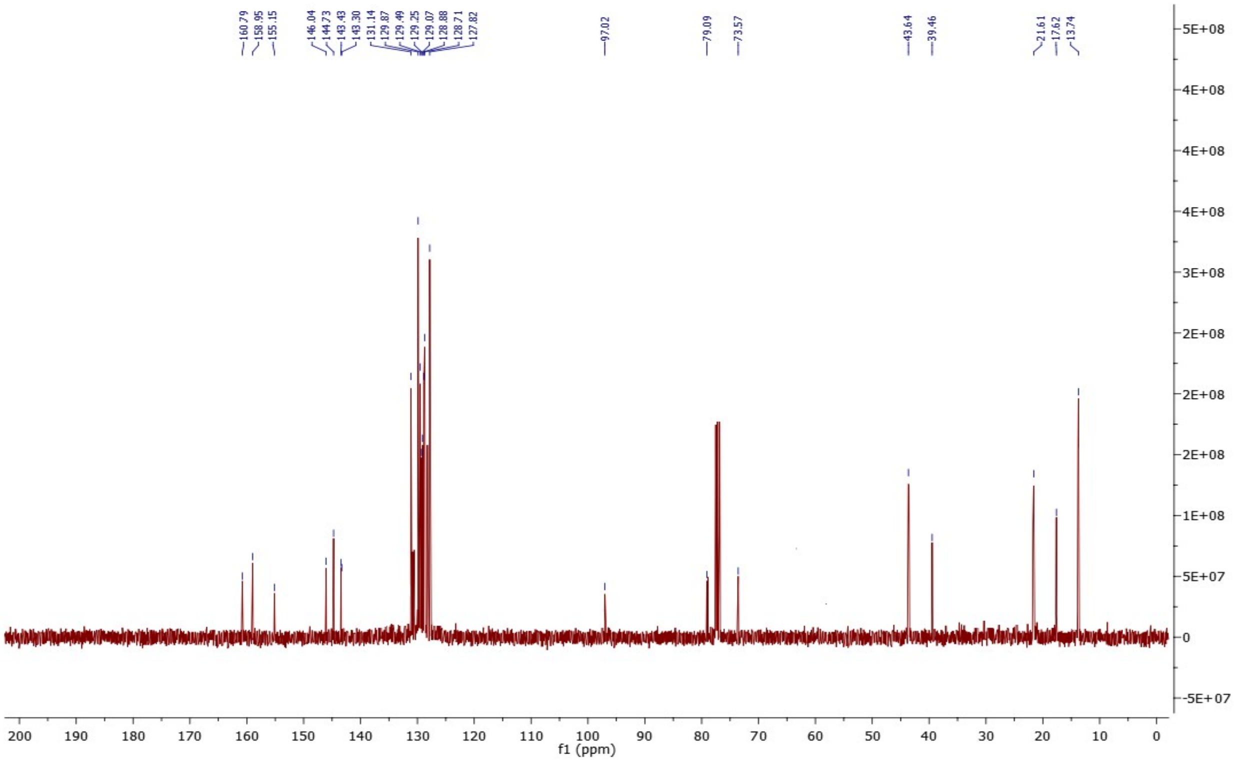

Figure 8. Carbon-13 NMR spectrum of 3c ($CDCl_3$).

Elemental Composition Report **Page 1**

Single Mass Analysis

Tolerance = 100.0 PPM / DBE: min = -1.5, max = 50.0
Element prediction: Off
Number of isotope peaks used for i-FIT = 3

Monoisotopic Mass, Even Electron Ions
49 formula(e) evaluated with 1 results within limits (up to 3 closest results for each mass)
Elements Used:
C: 0-31 H: 0-100 N: 0-4 O: 0-3 S: 0-1

RAY499 QMI DIVISION, CSIR-IIIM JAMMU 05-Oct-2023
Xevo G2-XS QTOF YFC2015 13:08:33
051023_24 4 (0.104) 1: TOF MS ES+ 5.59e+006

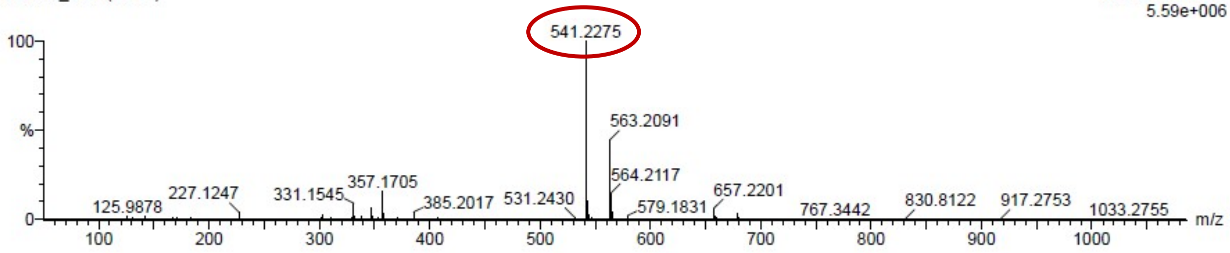

Minimum:				-1.5				
Maximum:		2.0	100.0	50.0				
Mass	Calc. Mass	mDa	PPM	DBE	i-FIT	Norm	Conf(%)	Formula
541.2275	541.2273	0.2	0.4	17.5	567.7	n/a	n/a	C31 H33 N4 O3 S

Figure 9. HRMS Spectrum of 3c

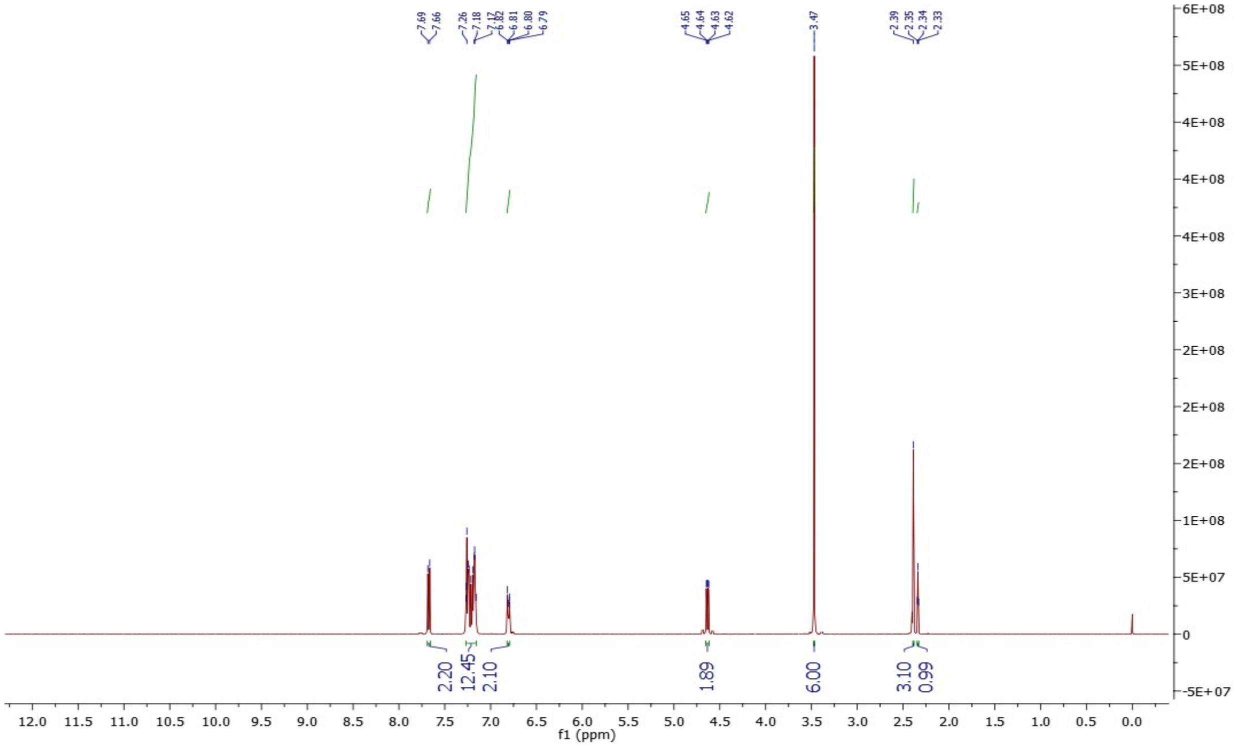

Figure 10. Proton NMR spectrum of 3d ($CDCl_3$).

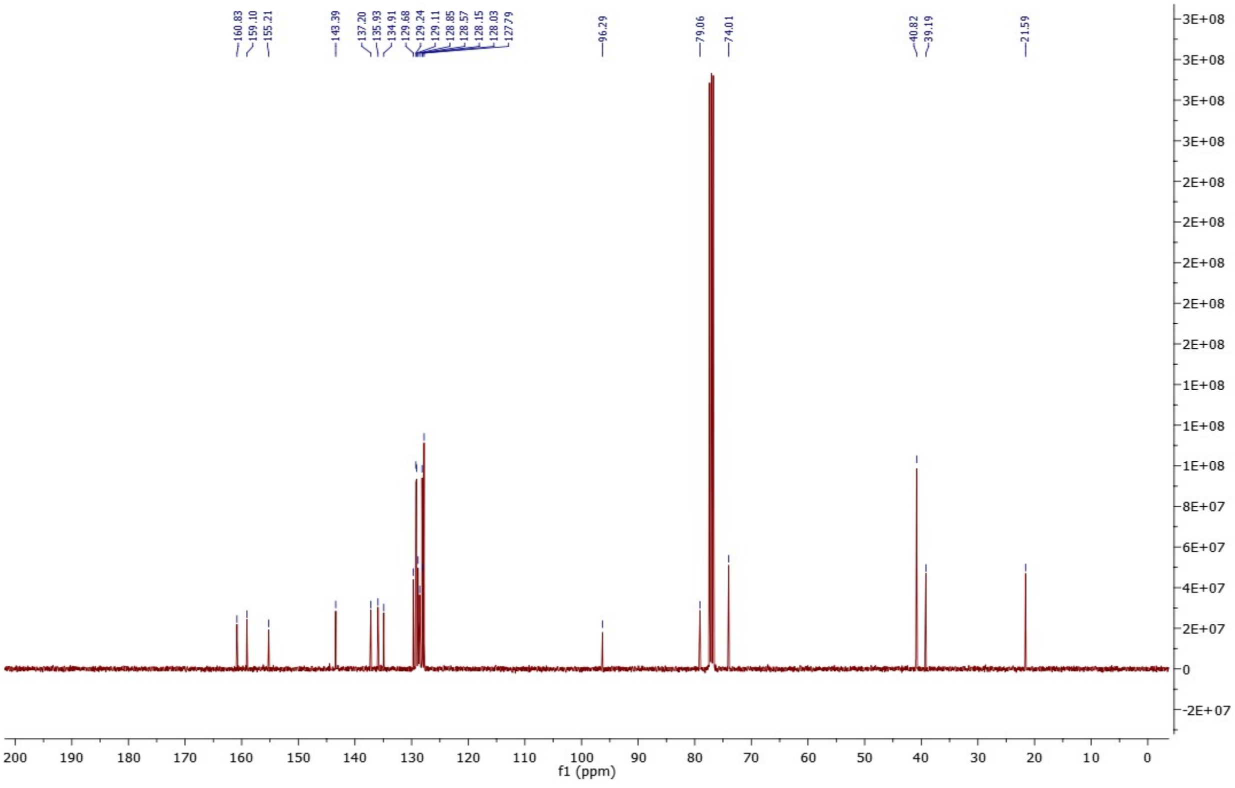

Figure 11. Carbon-13 spectrum of 3d ($CDCl_3$).

Elemental Composition Report **Page 1**

Single Mass Analysis
Tolerance = 100.0 PPM / DBE: min = -1.5, max = 50.0
Element prediction: Off
Number of isotope peaks used for i-FIT = 3

Monoisotopic Mass, Even Electron Ions
49 formula(e) evaluated with 1 results within limits (up to 3 closest results for each mass)
Elements Used:
C: 0-28 H: 0-100 N: 0-4 O: 0-3 S: 0-1

RAY238 QMI DIVISION, CSIR-IIIM JAMMU 05-Oct-2023
Xevo G2-XS QTOF YFC2015 13:11:07
051023_25 8 (0.172) 1: TOF MS ES+
2.20e+007

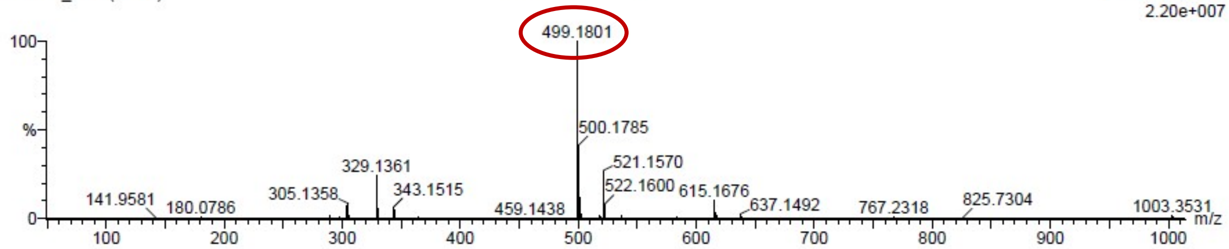

Minimum:				-1.5				
Maximum:		2.0	100.0	50.0				
Mass	Calc. Mass	mDa	PPM	DBE	i-FIT	Norm	Conf(%)	Formula
499.1801	499.1804	-0.3	-0.6	17.5	759.7	n/a	n/a	C28 H27 N4 O3 S

Figure 12. HRMS Spectrum of 3d

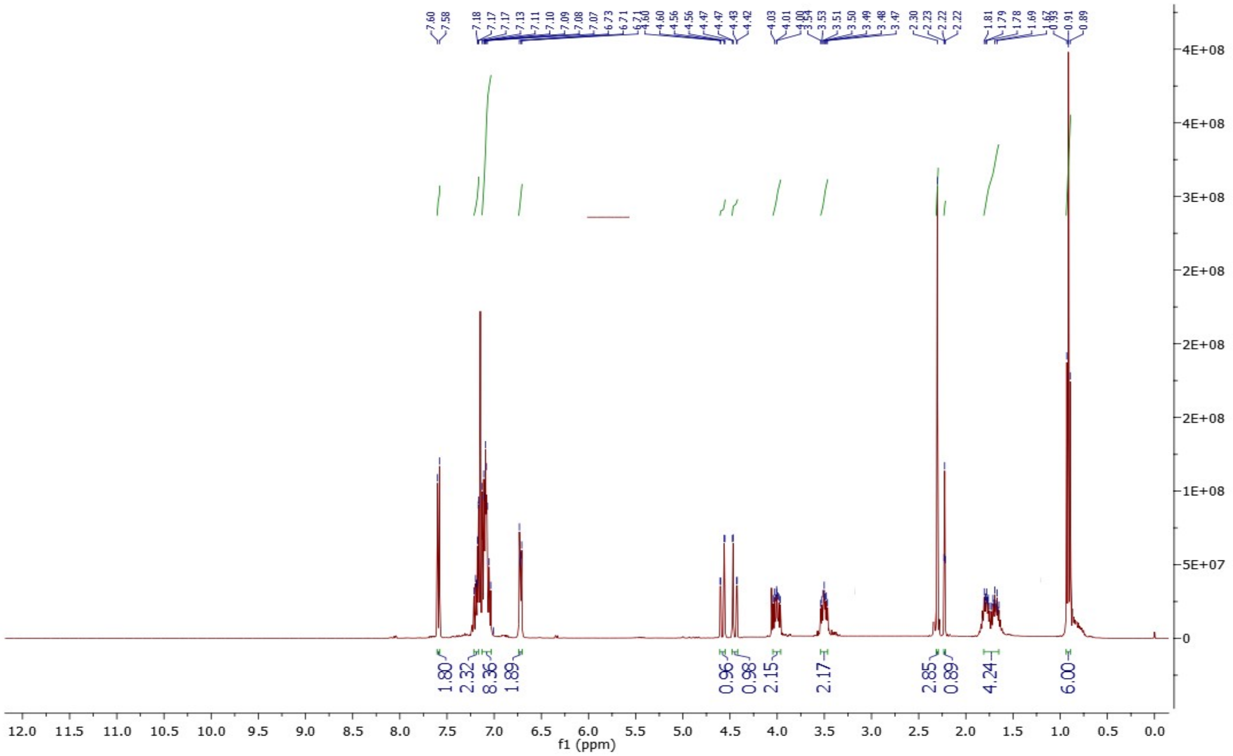

Figure 13. Proton NMR spectrum of 3e ($CDCl_3$).

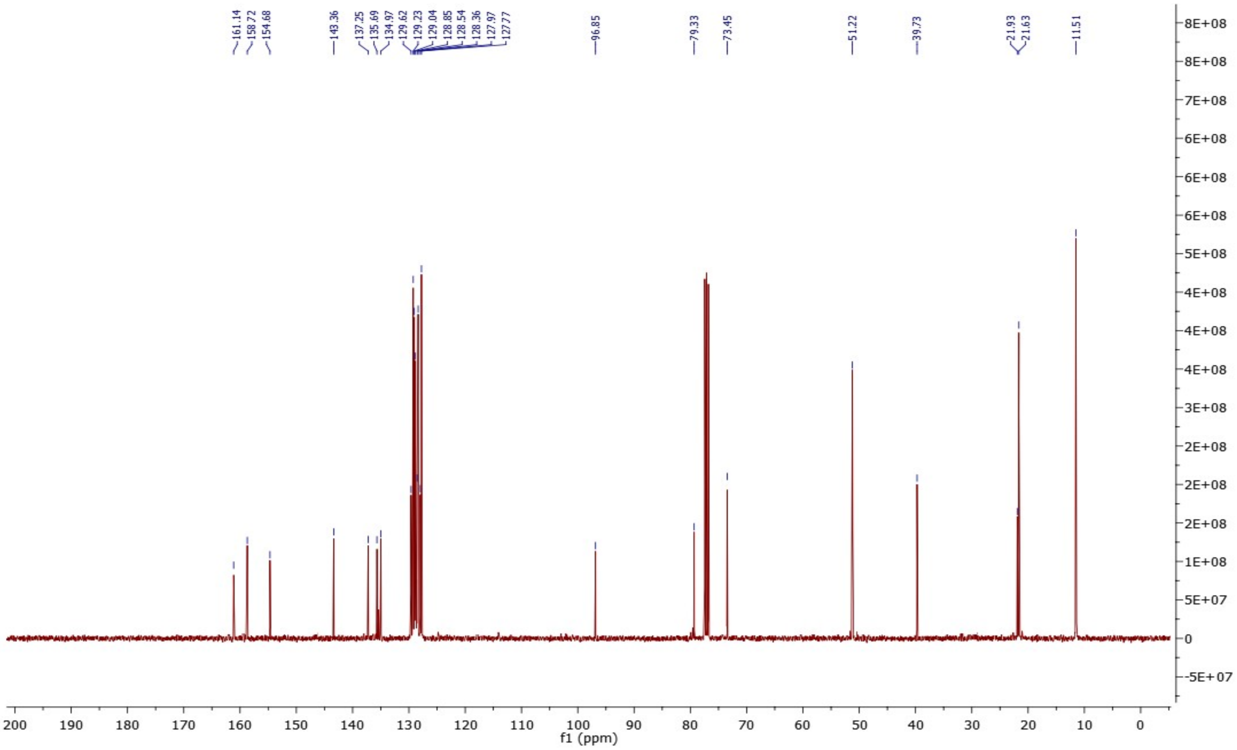

Figure 14. Carbon-13 spectrum of 3e ($CDCl_3$).

Elemental Composition Report **Page 1**

Single Mass Analysis
Tolerance = 100.0 PPM / DBE: min = -1.5, max = 50.0
Element prediction: Off
Number of isotope peaks used for i-FIT = 3

Monoisotopic Mass, Even Electron Ions
49 formula(e) evaluated with 1 results within limits (up to 3 closest results for each mass)
Elements Used:
C: 0-32 H: 0-100 N: 0-4 O: 0-3 S: 0-1

RAY549 QMI DIVISION, CSIR-IIIM JAMMU 05-Oct-2023
Xevo G2-XS QTOF YFC2015 13:00:51
051023_21 15 (0.310) 1: TOF MS ES+
5.77e+005

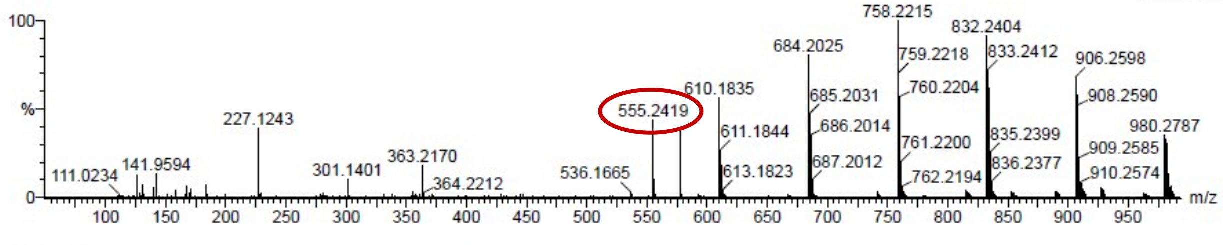

Minimum:				-1.5				
Maximum:		2.0	100.0	50.0				
Mass	Calc. Mass	mDa	PPM	DBE	i-FIT	Norm	Conf(%)	Formula
555.2419	555.2430	-1.1	-2.0	17.5	506.8	n/a	n/a	C32 H35 N4 O3 S

Figure 15. HRMS Spectrum of 3e

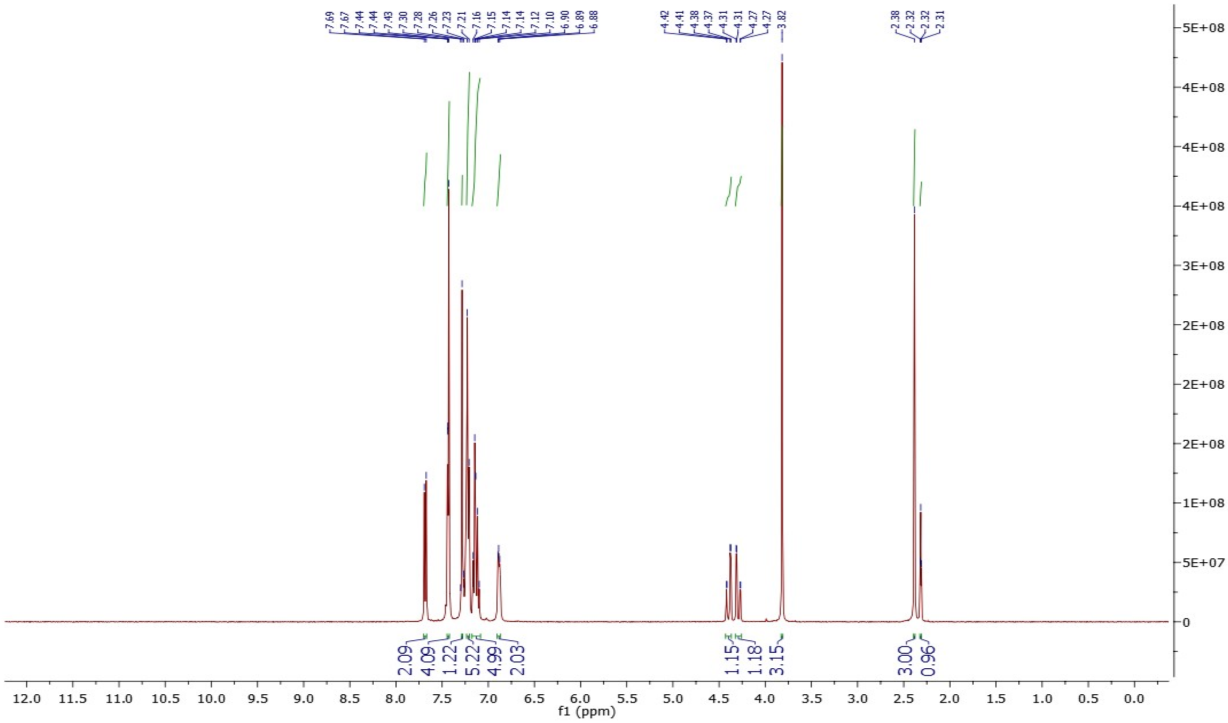

Figure 16. Proton NMR spectrum of 3f ($CDCl_3$).

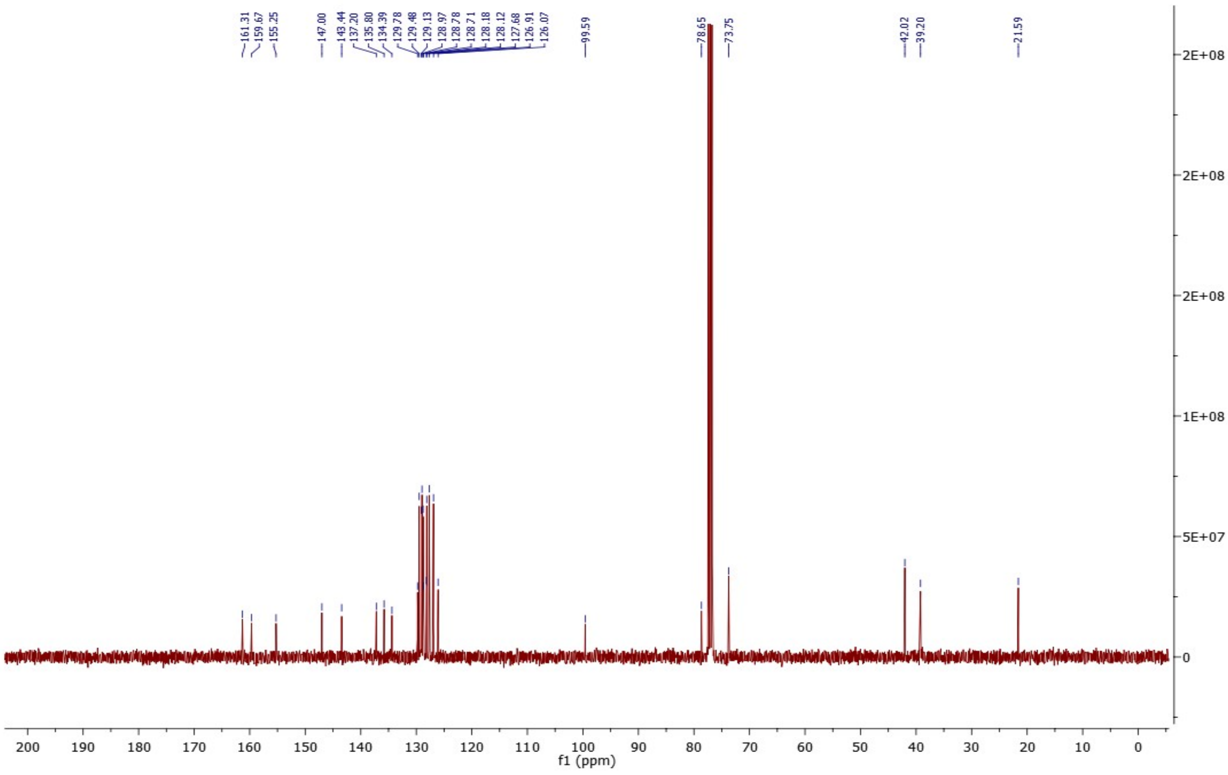

Figure 17. Carbon-NMR spectrum of 3f ($CDCl_3$).

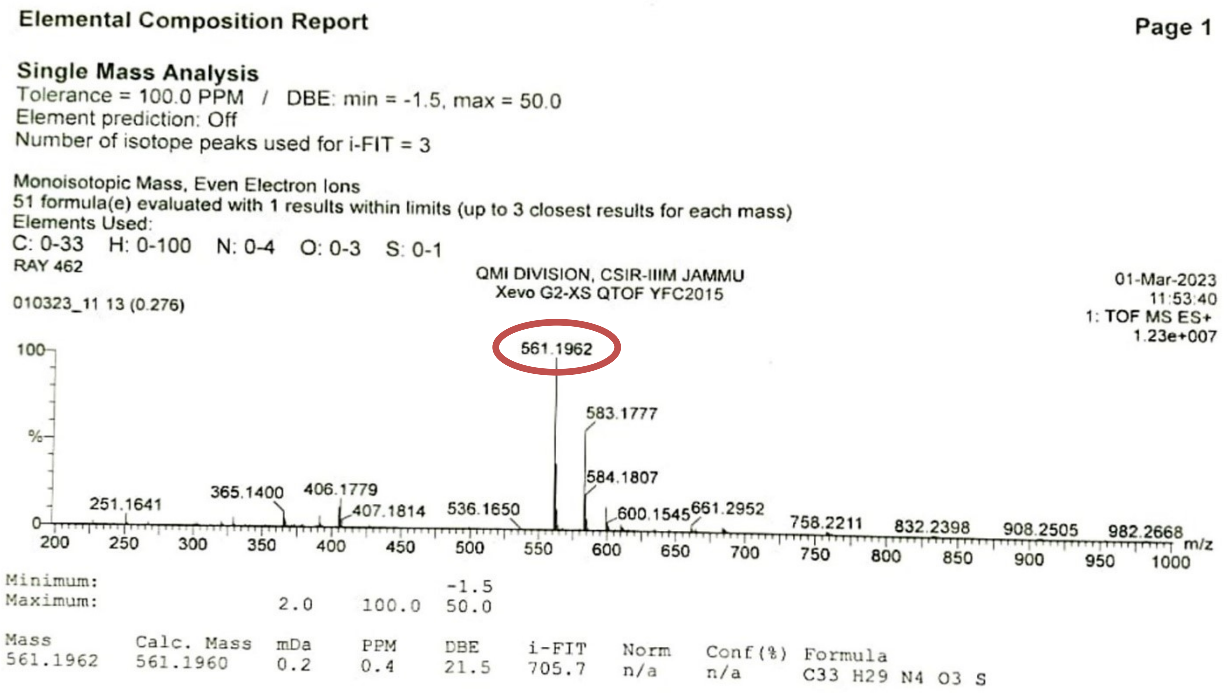

Figure 18. HRMS Spectrum of 3f.

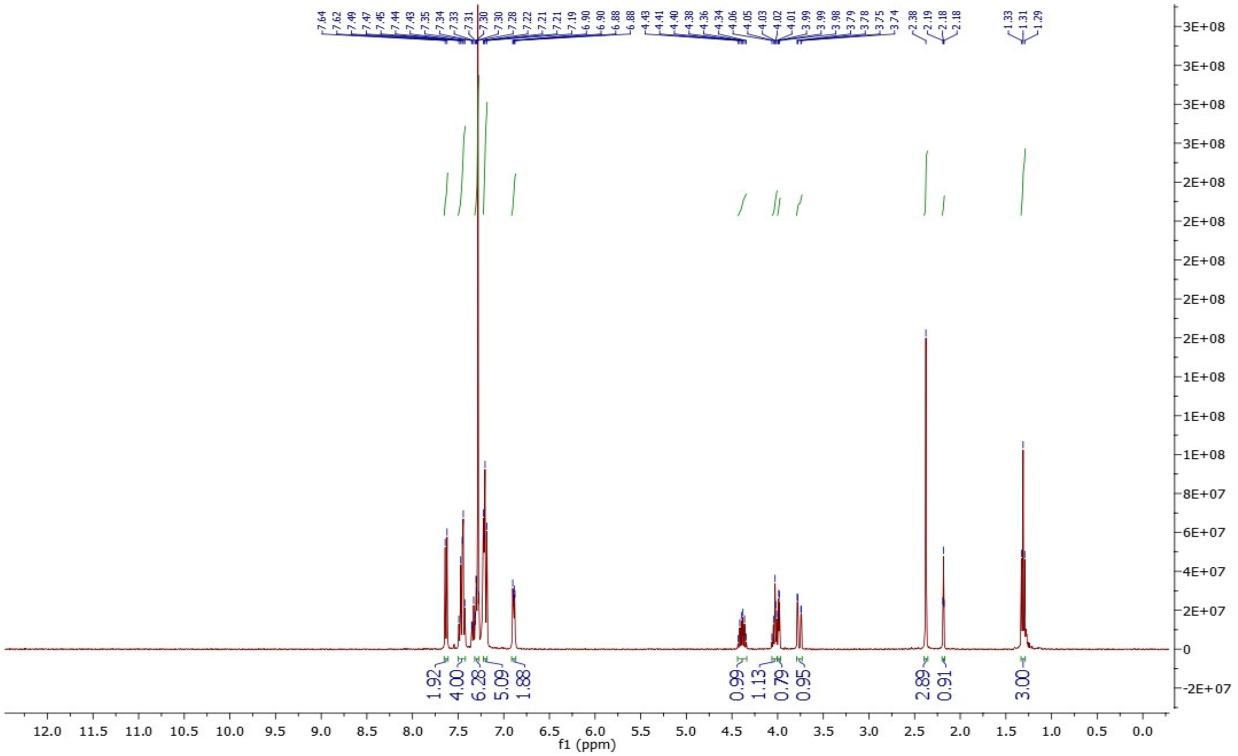

Figure 19. Proton NMR spectrum of 3g ($CDCl_3$).

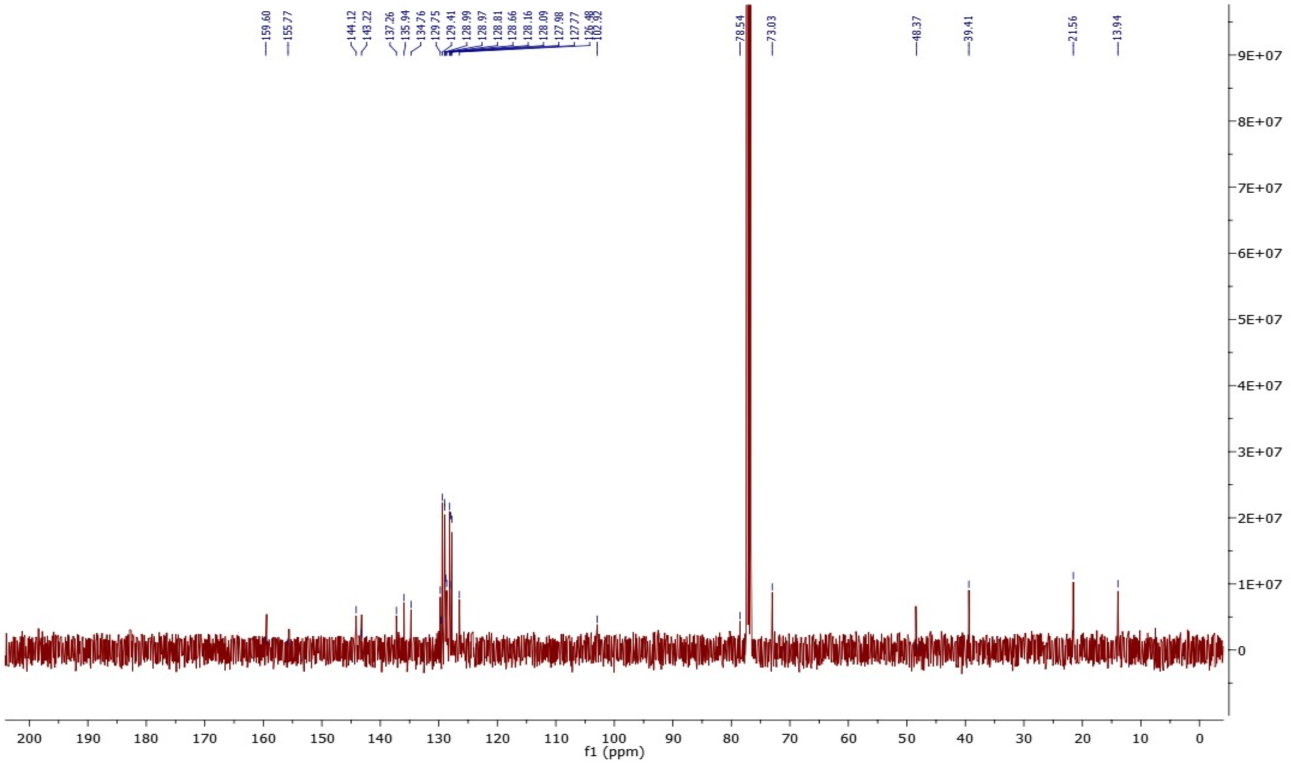

Figure 20. Carbon-13 NMR spectrum of 3g ($CDCl_3$).

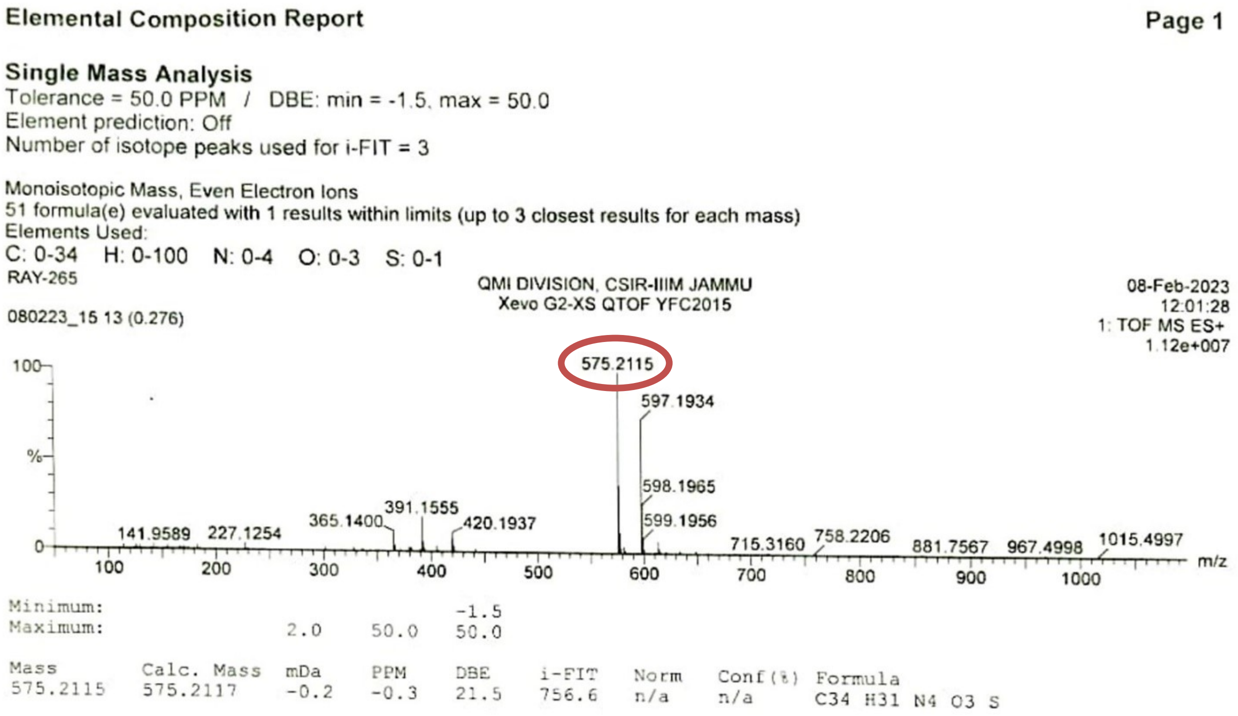

Figure 21. HRMS Spectrum of 3g

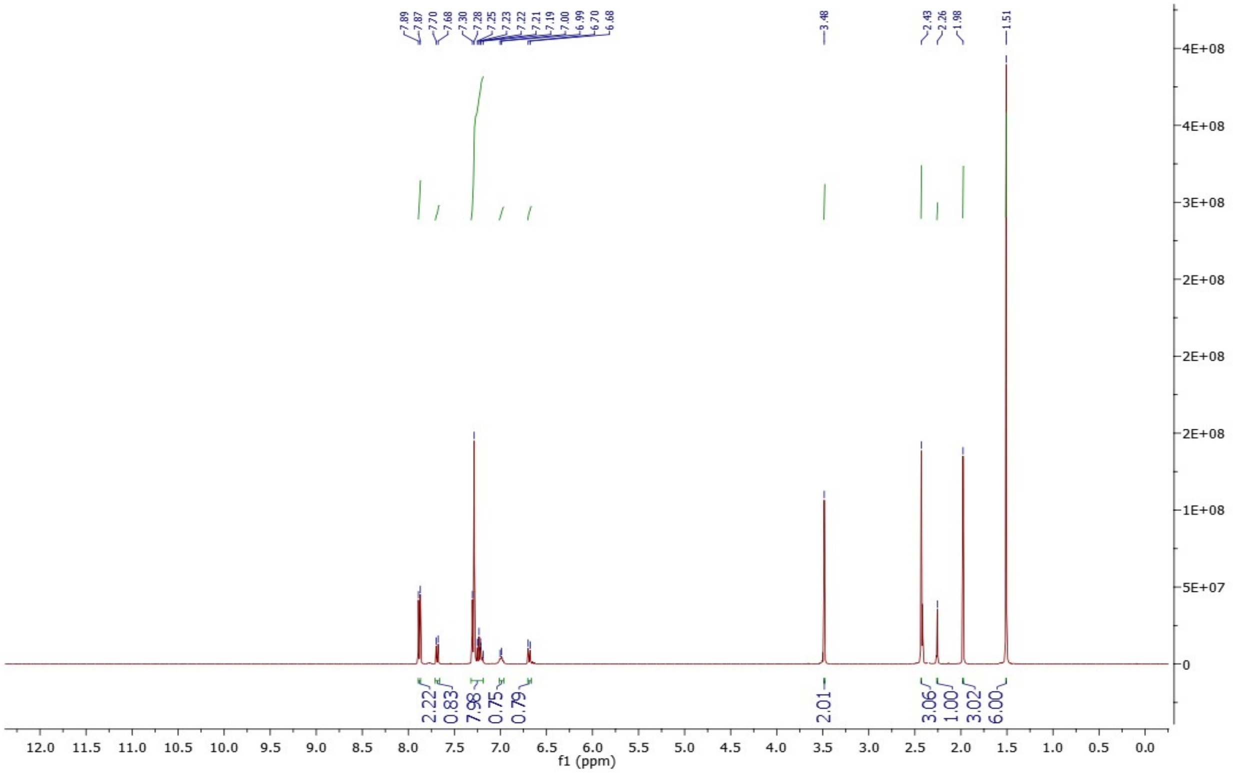

Figure 22. Proton NMR spectrum of 3h ($CDCl_3$).

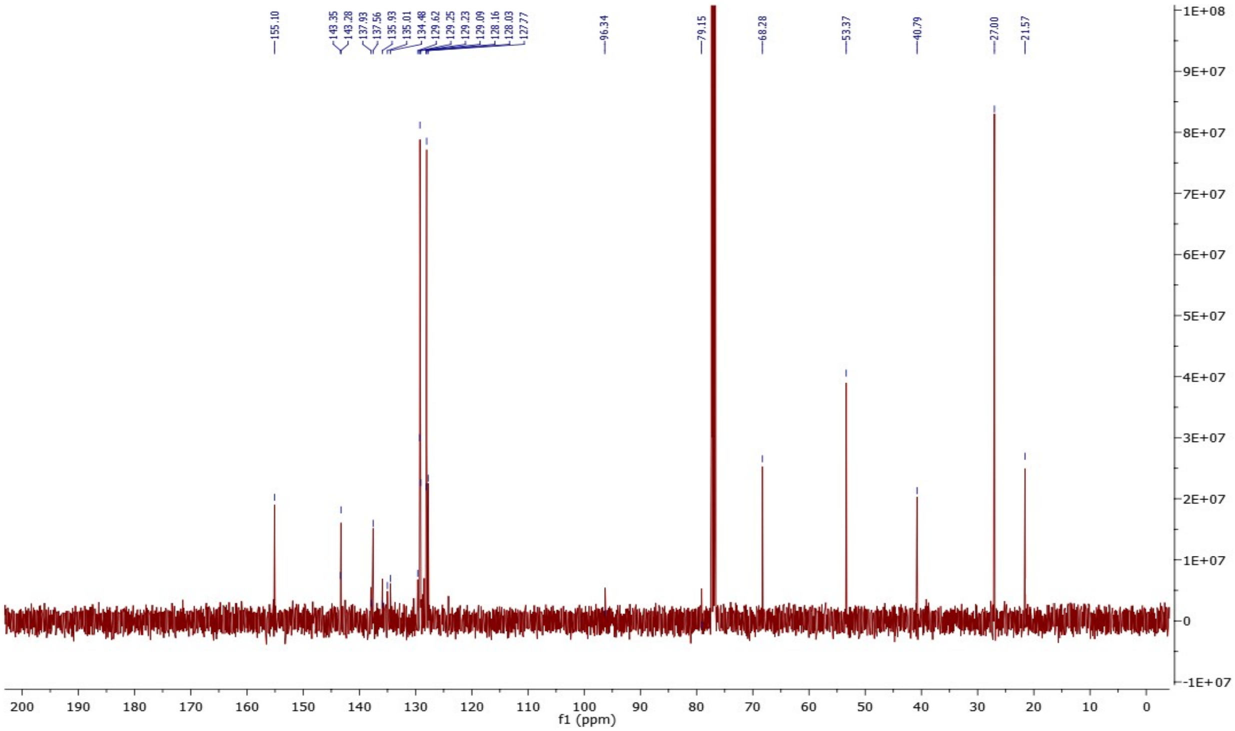

Figure 23. Carbon-13 NMR spectrum of 3h ($CDCl_3$).

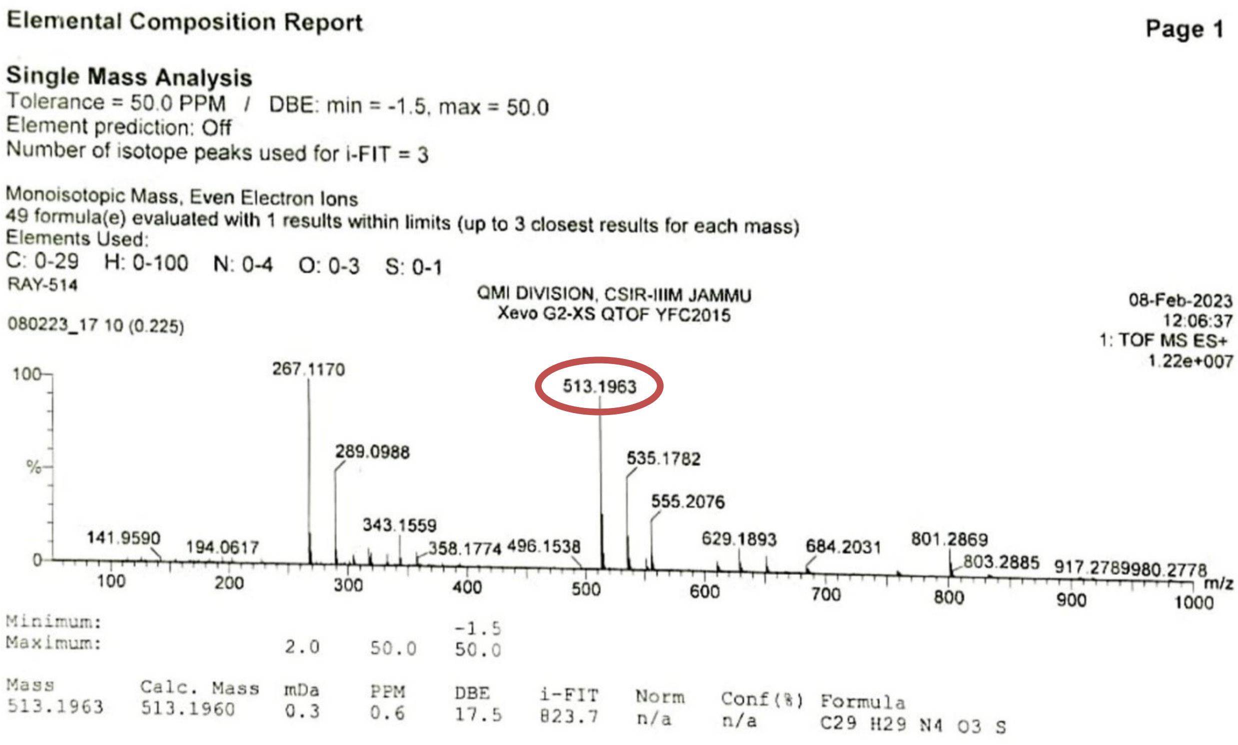
Elemental Composition Report

Page 1

Single Mass Analysis
Tolerance = 50.0 PPM / DBE: min = -1.5, max = 50.0
Element prediction: Off
Number of isotope peaks used for i-FIT = 3

Monoisotopic Mass, Even Electron Ions
49 formula(e) evaluated with 1 results within limits (up to 3 closest results for each mass)
Elements Used:
C: 0-29 H: 0-100 N: 0-4 O: 0-3 S: 0-1
RAY-514

QMI DIVISION, CSIR-IIIM JAMMU
Xevo G2-XS QTOF YFC2015

080223_17 10 (0.225)

08-Feb-2023
12:06:37
1: TOF MS ES+
1.22e+007

Minimum:				-1.5				
Maximum:		2.0	50.0	50.0				
Mass	Calc. Mass	mDa	PPM	DBE	i-FIT	Norm	Conf(%)	Formula
513.1963	513.1960	0.3	0.6	17.5	823.7	n/a	n/a	C29 H29 N4 O3 S

Figure 24. HRMS Spectrum of 3h

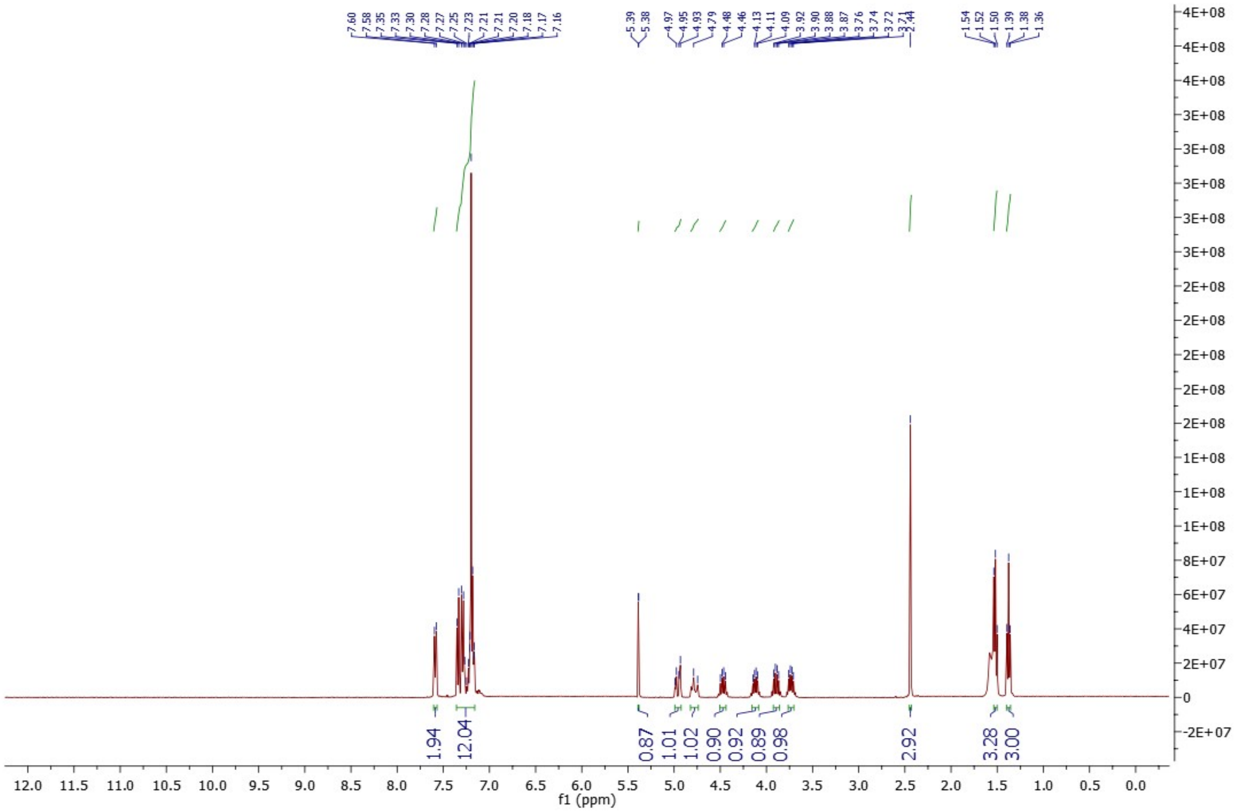

Figure 25: Proton NMR spectrum of 4a ($CDCl_3$).

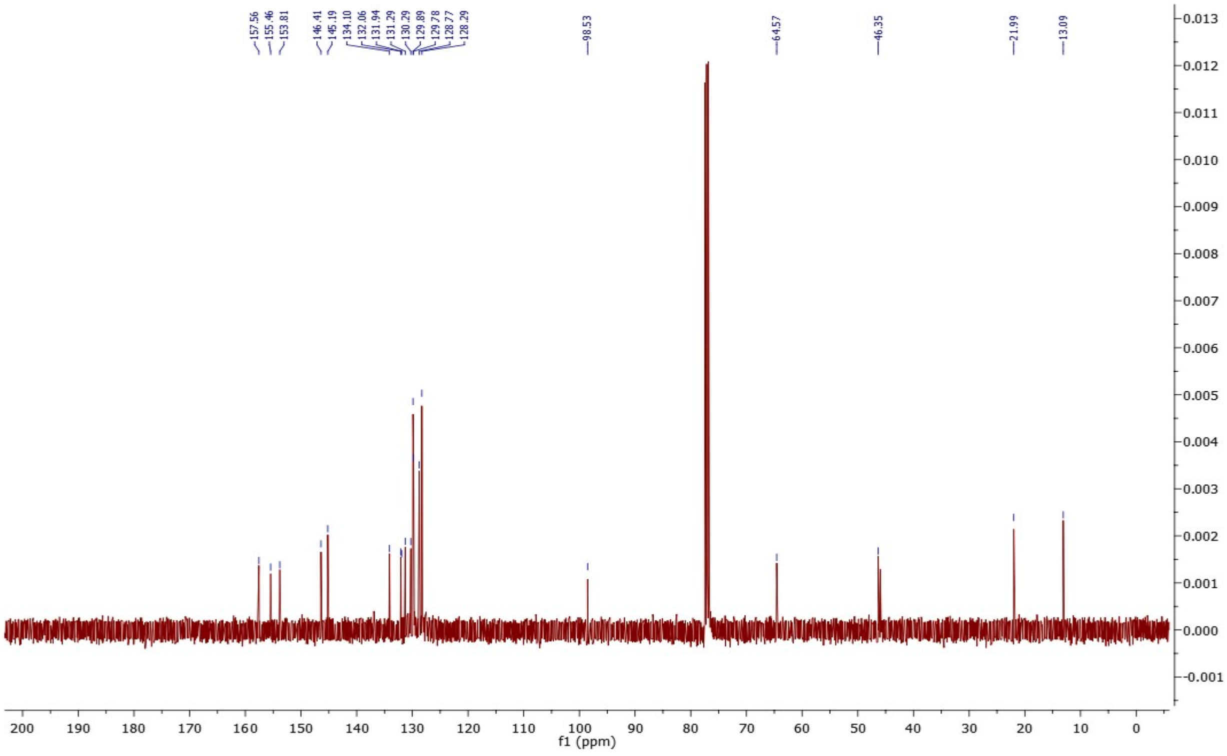

Figure 26. Carbon-13 NMR spectrum of 4a ($CDCl_3$).

Elemental Composition Report **Page 1**

Single Mass Analysis
Tolerance = 100.0 PPM / DBE: min = -1.5, max = 50.0
Element prediction: Off
Number of isotope peaks used for i-FIT = 3

Monoisotopic Mass, Even Electron Ions
89 formula(e) evaluated with 1 results within limits (up to 3 closest results for each mass)
Elements Used:
C: 0-30 H: 0-100 N: 0-4 O: 0-3 S: 0-1 I: 0-1

RAY 455 QMI DIVISION, CSIR-IIIM JAMMU Xevo G2-XS QTOF YFC2015 23-Feb-2023 11:34:00
230223_07 38 (0.759) 1: TOF MS ES+ 1.92e+007

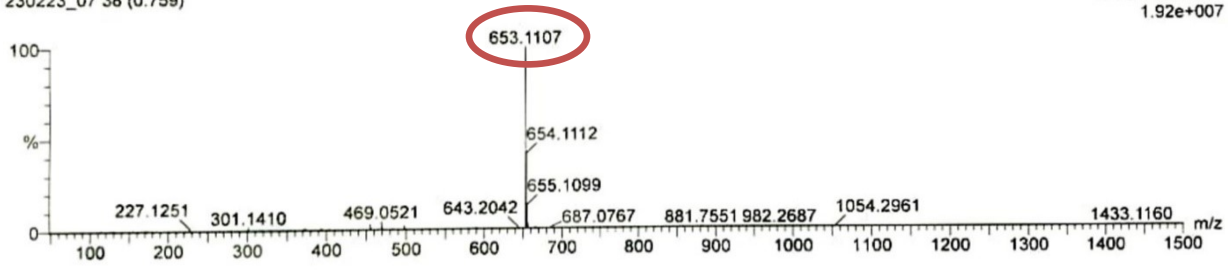

Minimum:				-1.5				
Maximum:		2.0	100.0	50.0				
Mass	Calc. Mass	mDa	PPM	DBE	i-FIT	Norm	Conf(%)	Formula
653.1107	653.1083	2.4	3.7	17.5	600.5	n/a	n/a	C30 H30 N4 O3 S I

Figure 27. HRMS Spectrum of 4a

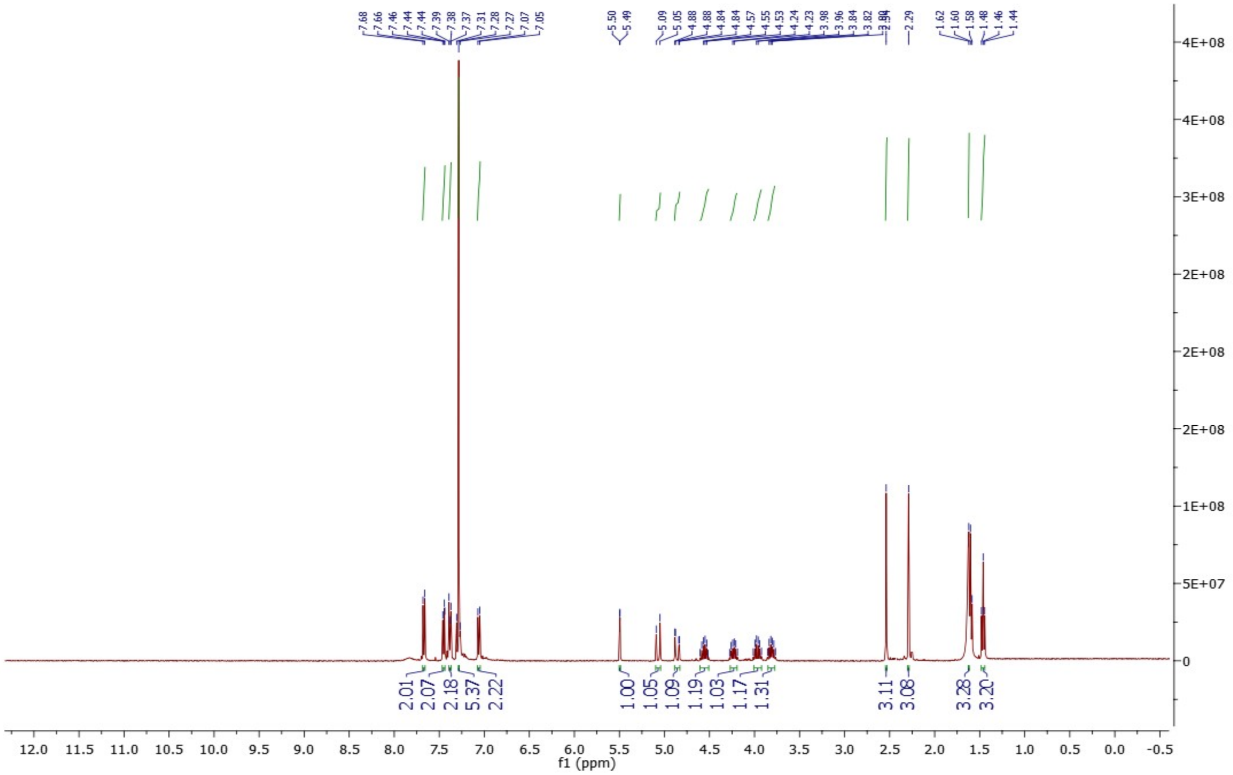

Figure 28. Proton NMR spectrum of 4b ($CDCl_3$).

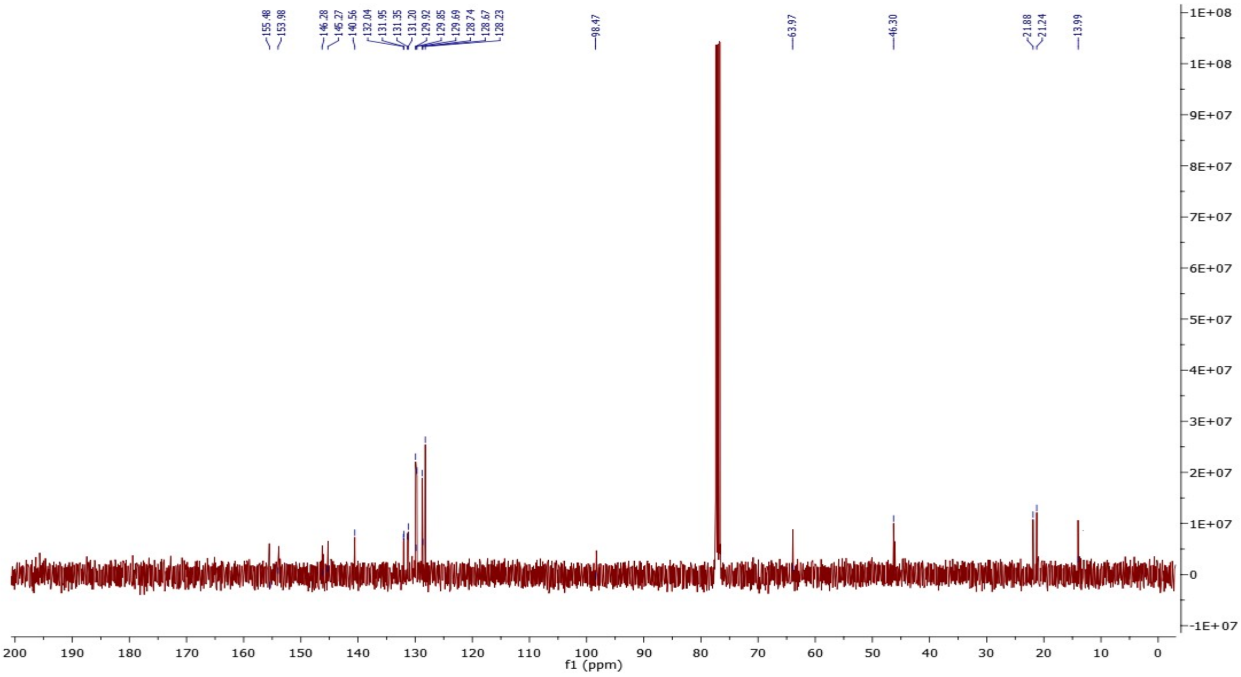

Figure 29. Carbon-13 NMR spectrum of 4b ($CDCl_3$).

Elemental Composition Report **Page 1**

Single Mass Analysis
Tolerance = 50.0 PPM / DBE: min = -1.5, max = 50.0
Element prediction: Off
Number of isotope peaks used for i-FIT = 3

Monoisotopic Mass, Even Electron Ions
89 formula(e) evaluated with 1 results within limits (up to 3 closest results for each mass)
Elements Used:
C: 0-31 H: 0-100 N: 0-4 O: 0-3 S: 0-1 I: 0-1

RAY-518 QMI DIVISION, CSIR-IIIM JAMMU 02-Feb-2023
Xevo G2-XS QTOF YFC2015 14:55:27
2022023_03 11 (0.242) 1: TOF MS ES+ 1.72e+007

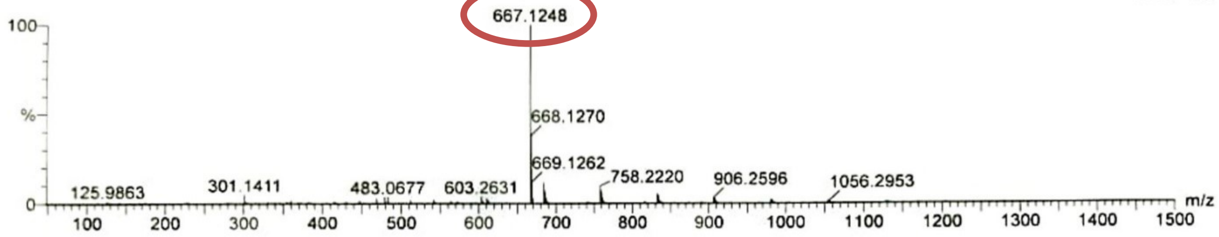

Minimum:				-1.5				
Maximum:		2.0	50.0	50.0				
Mass	Calc. Mass	mDa	PPM	DBE	i-FIT	Norm	Conf(%)	Formula
667.1248	667.1240	0.8	1.2	17.5	641.7	n/a	n/a	C31 H32 N4 O3 S I

Figure 30. HRMS Spectrum of 4b

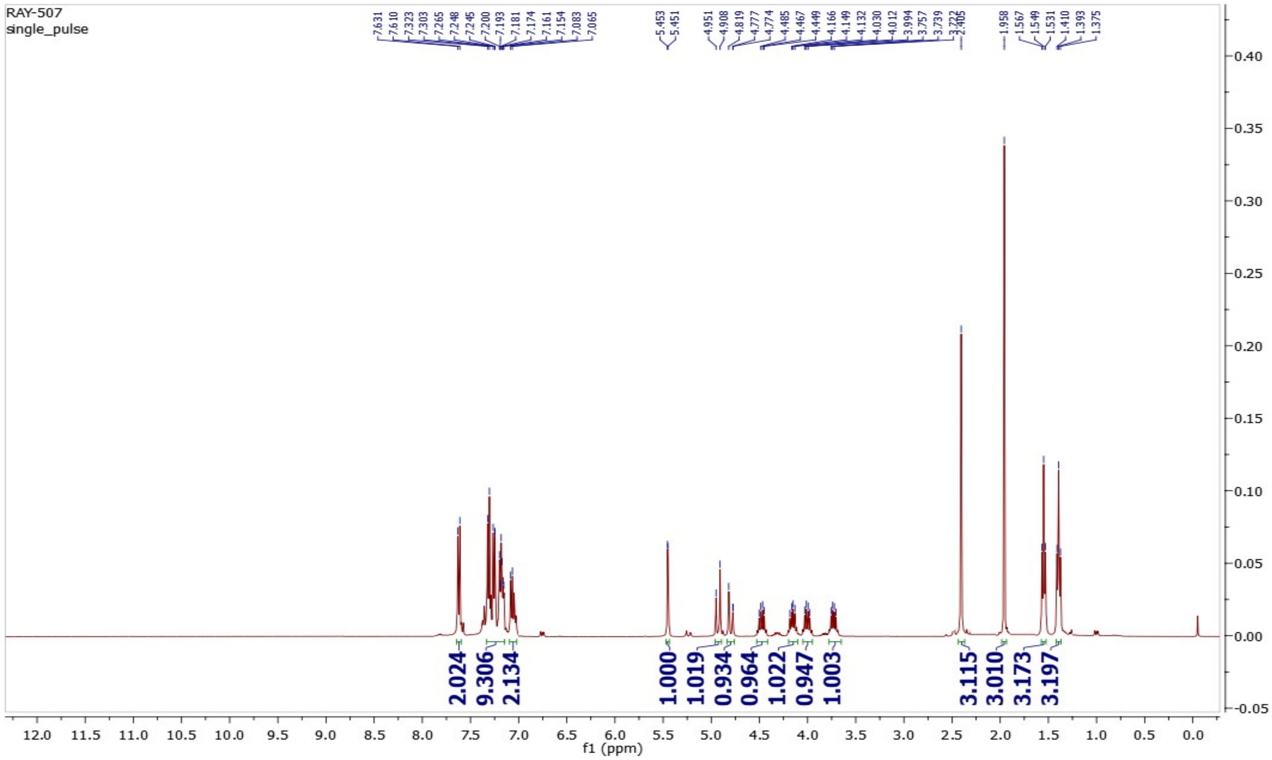

Figure 31. Proton NMR spectrum of 4c ($CDCl_3$).

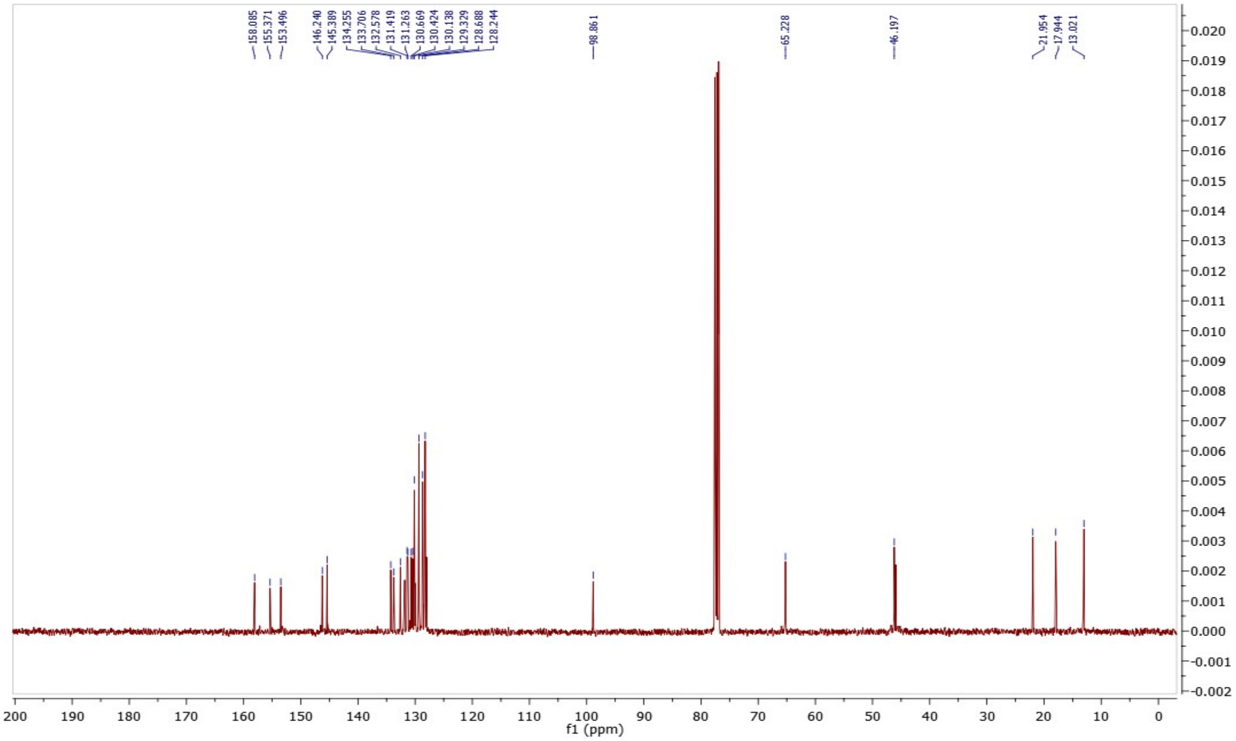

Figure 32. Carbon-13 NMR spectrum of 4c ($CDCl_3$).

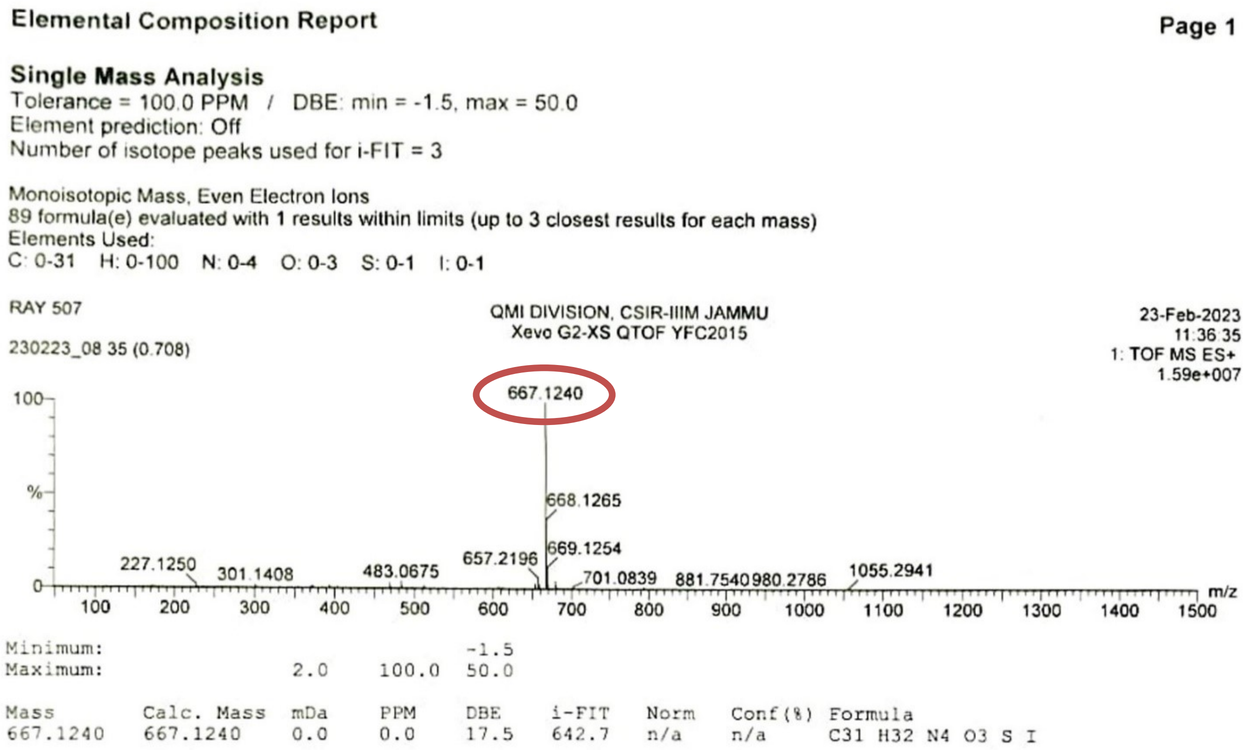
Elemental Composition Report

Page 1

Single Mass Analysis
Tolerance = 100.0 PPM / DBE: min = -1.5, max = 50.0
Element prediction: Off
Number of isotope peaks used for i-FIT = 3

Monoisotopic Mass, Even Electron Ions
89 formula(e) evaluated with 1 results within limits (up to 3 closest results for each mass)
Elements Used:
C: 0-31 H: 0-100 N: 0-4 O: 0-3 S: 0-1 I: 0-1

RAY 507
230223_08 35 (0.708)

QMI DIVISION, CSIR-IIIM JAMMU
Xevo G2-XS QTOF YFC2015

23-Feb-2023
11:36:35
1: TOF MS ES+
1.59e+007

		mDa	PPM	DBE				
Minimum:				-1.5				
Maximum:		2.0	100.0	50.0				

Mass	Calc. Mass	mDa	PPM	DBE	i-FIT	Norm	Conf(%)	Formula
667.1240	667.1240	0.0	0.0	17.5	642.7	n/a	n/a	C31 H32 N4 O3 S I

Figure 33. HRMS Spectrum of 4c

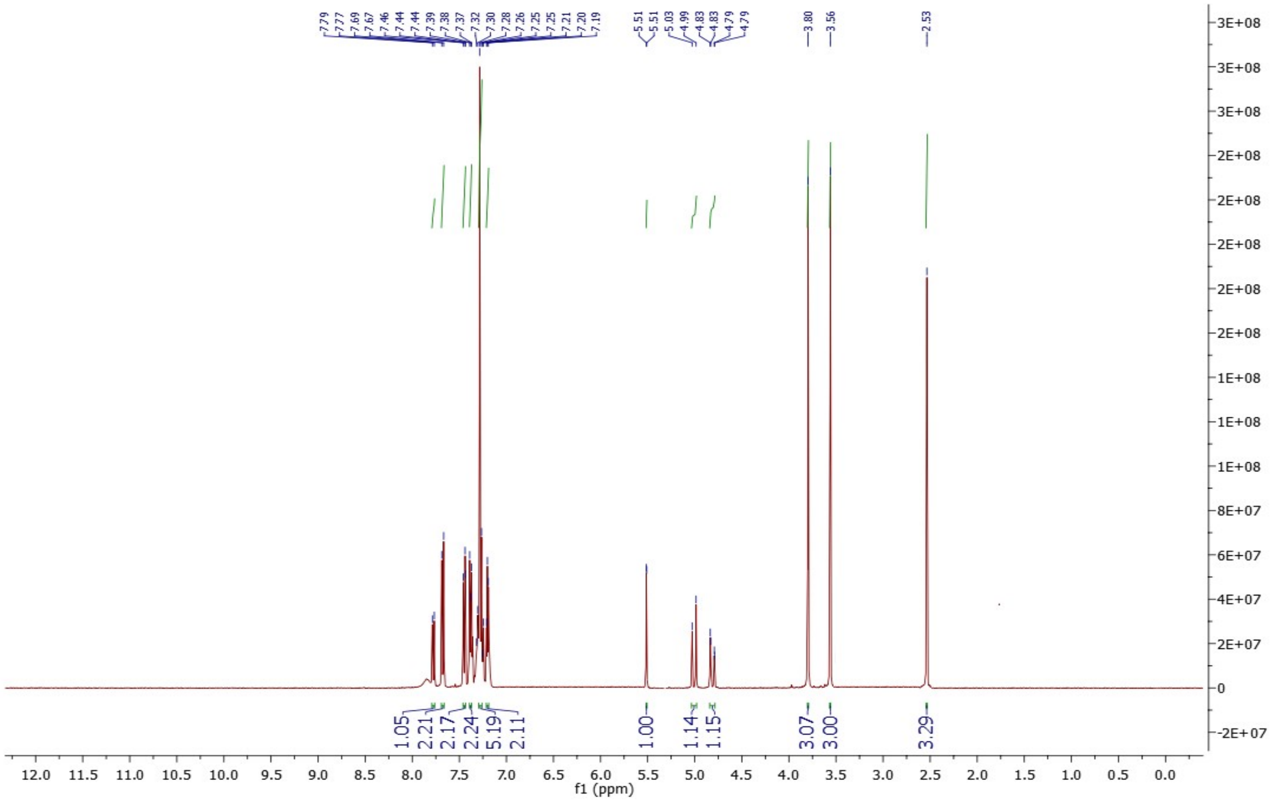

Figure 34. Proton NMR spectrum of 4d ($CDCl_3$).

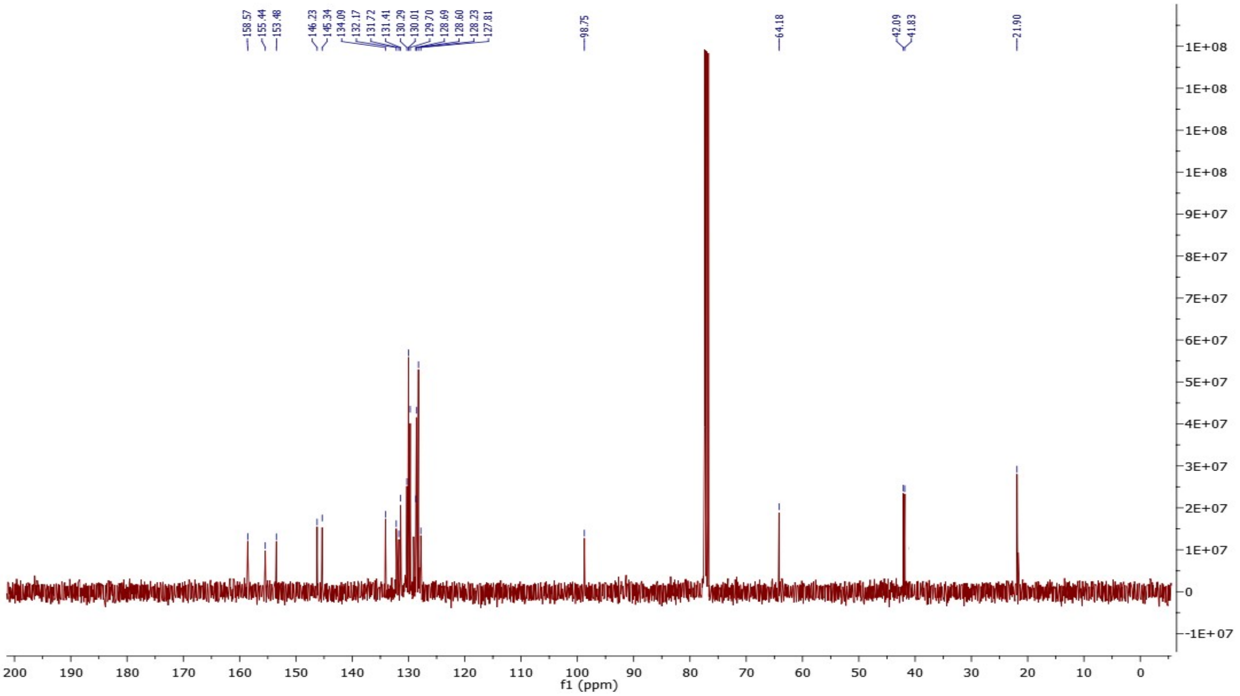

Figure 35. Carbon-13 NMR spectrum of 4d ($CDCl_3$).

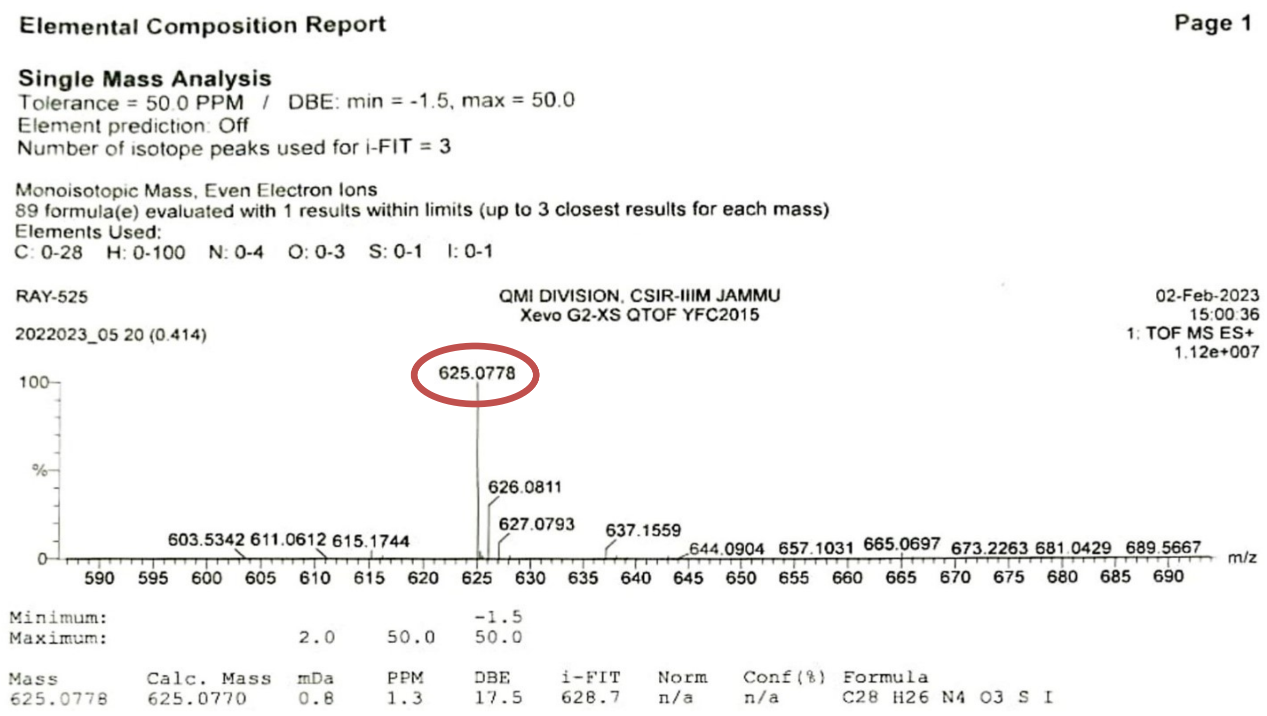

Figure 36. HRMS Spectrum of 4d

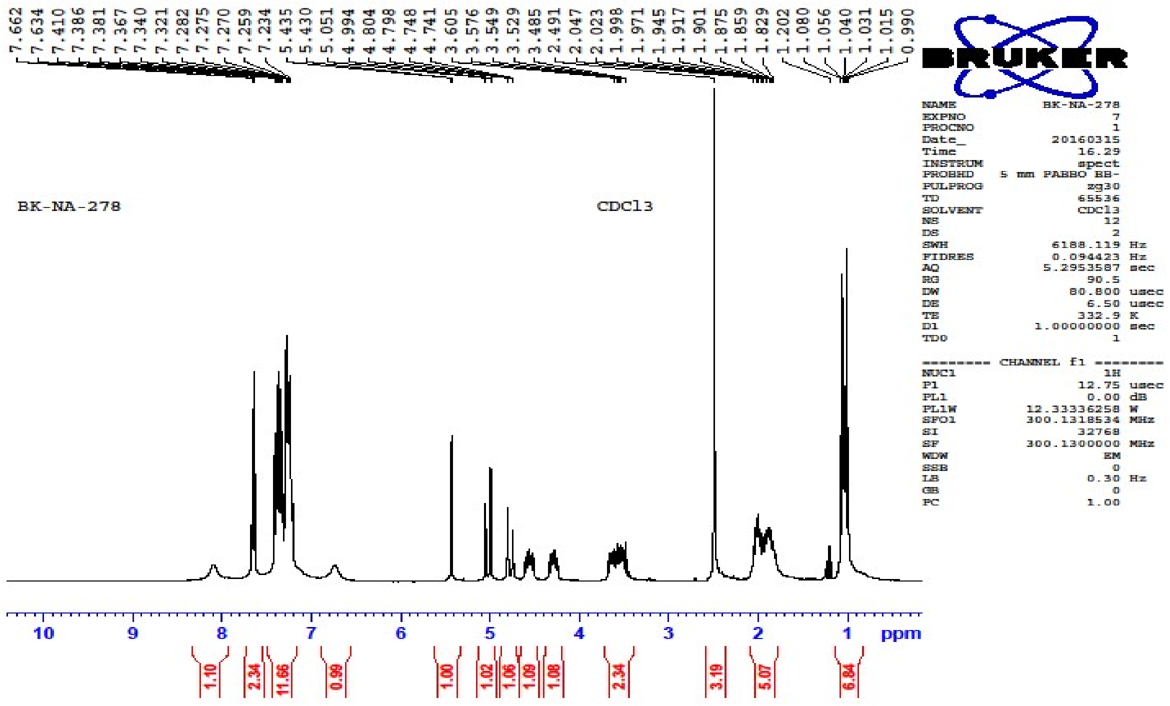

Figure 37. Proton NMR spectrum of 4e ($CDCl_3$).

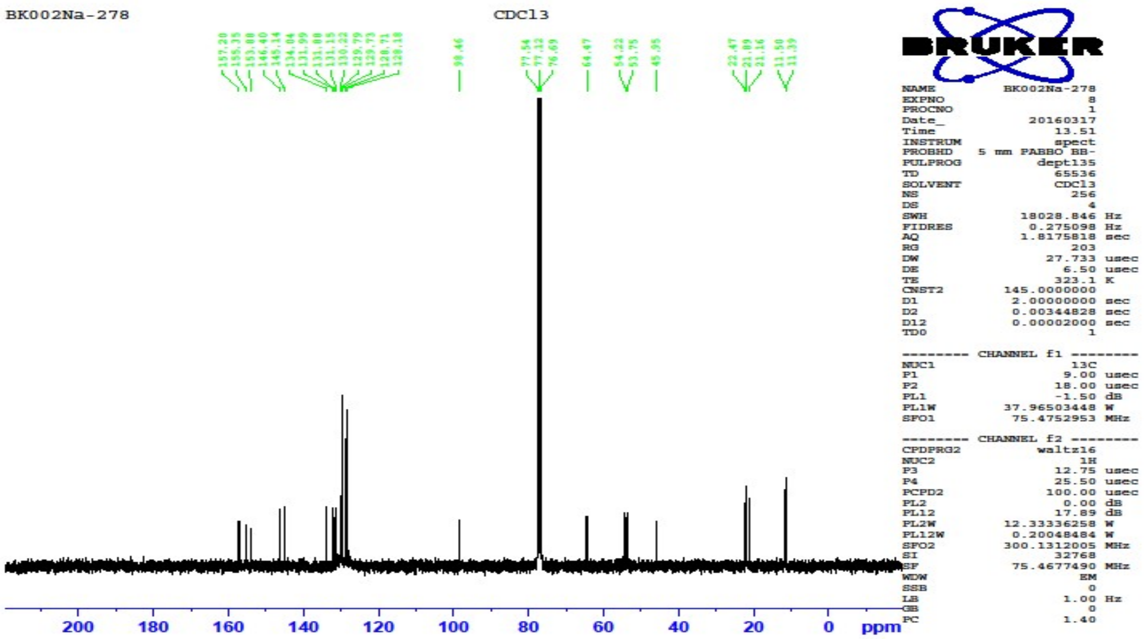

Figure 38. Carbon-13 NMR spectrum of 4e ($CDCl_3$).

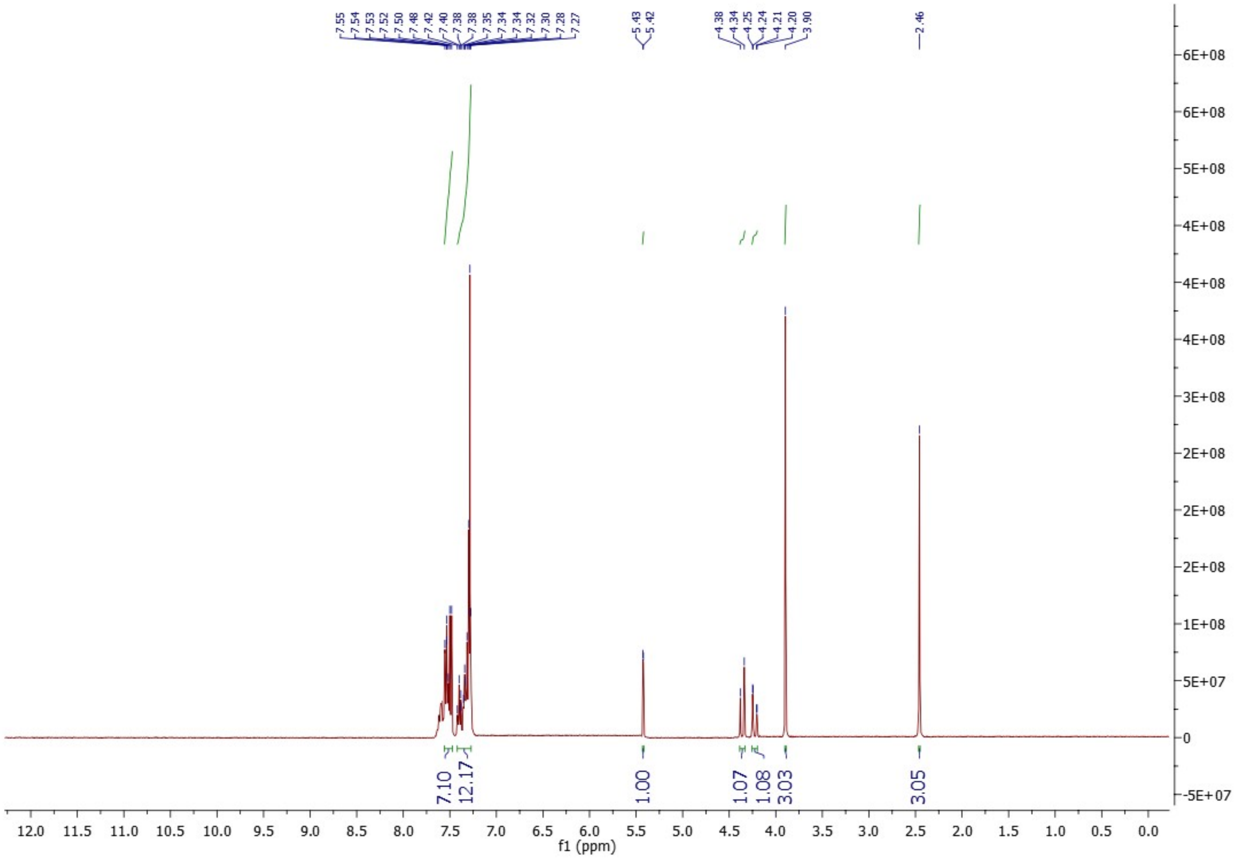

Figure 39. Proton NMR spectrum of 4f ($CDCl_3$).

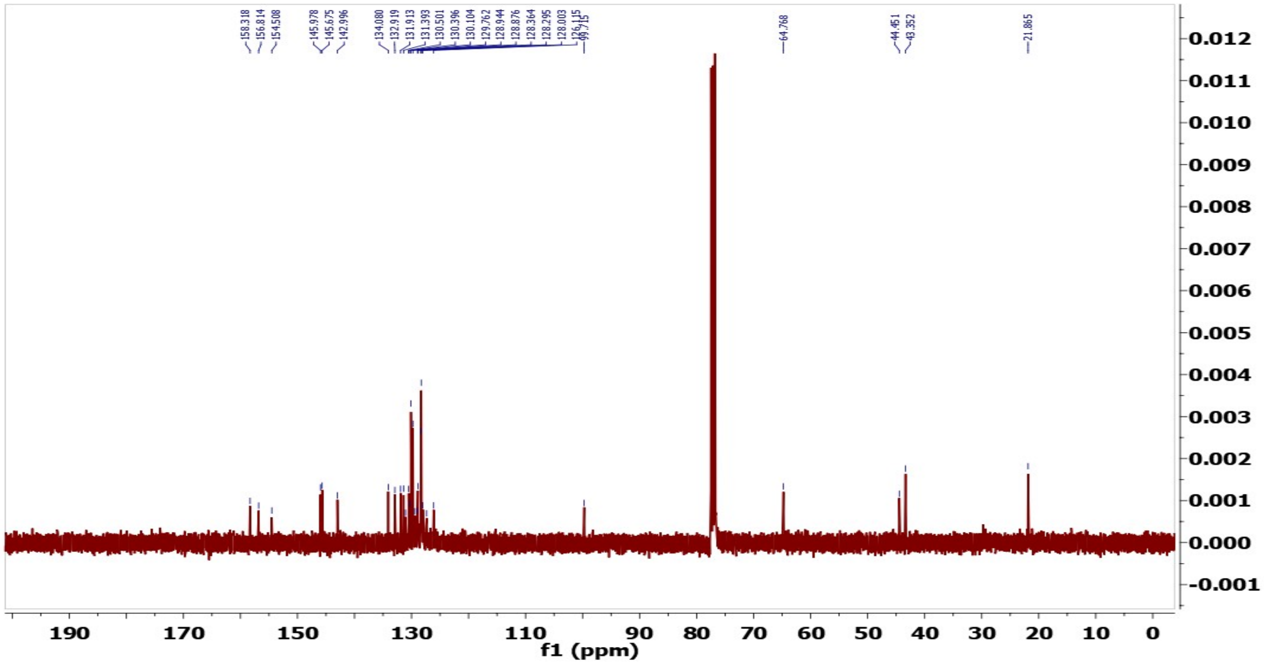

Figure 40. Carbon-13 NMR spectrum of 4f ($CDCl_3$).

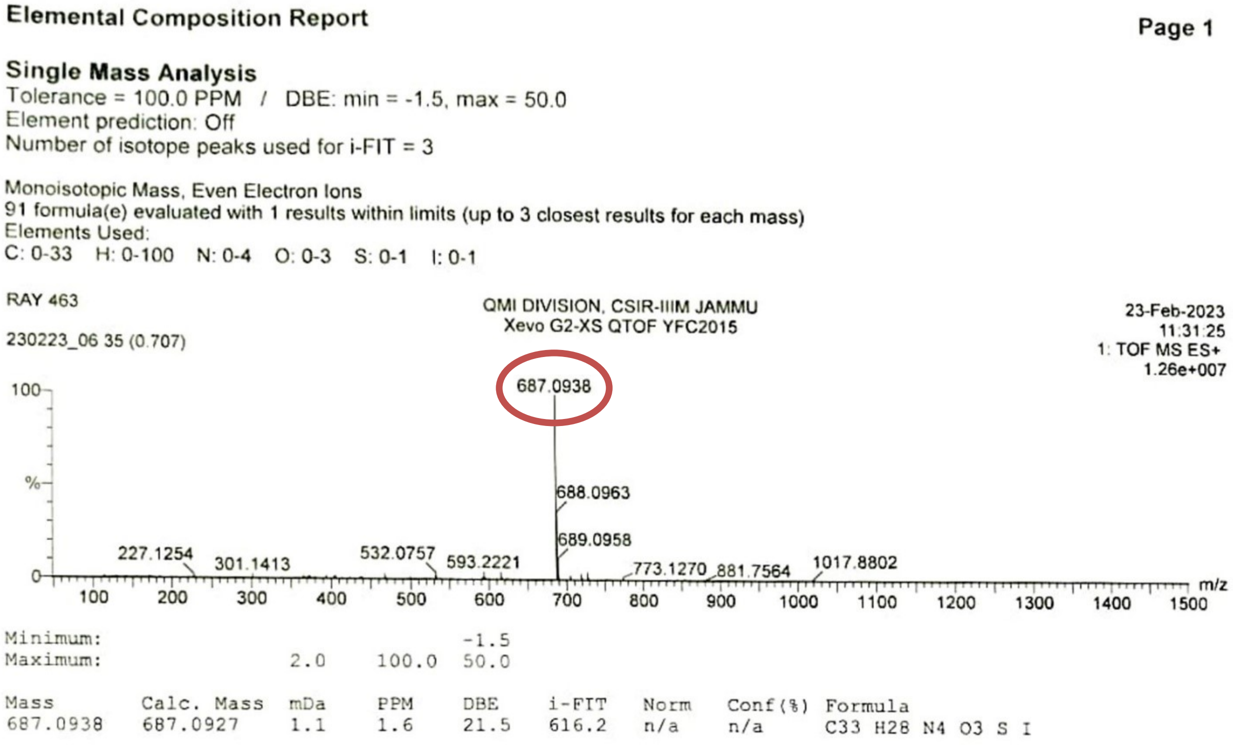

Figure 41. HRMS Spectrum of 4f

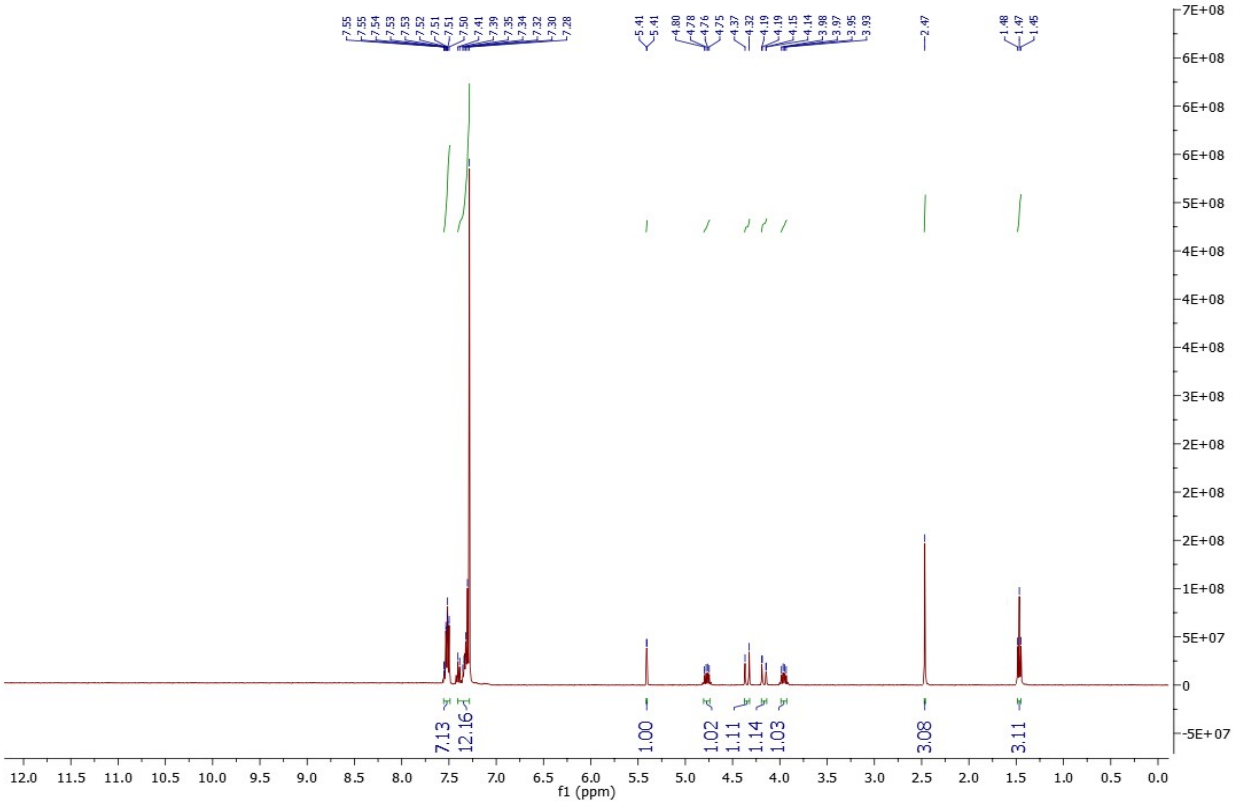

Figure 42. Proton NMR spectrum of 4g ($CDCl_3$).

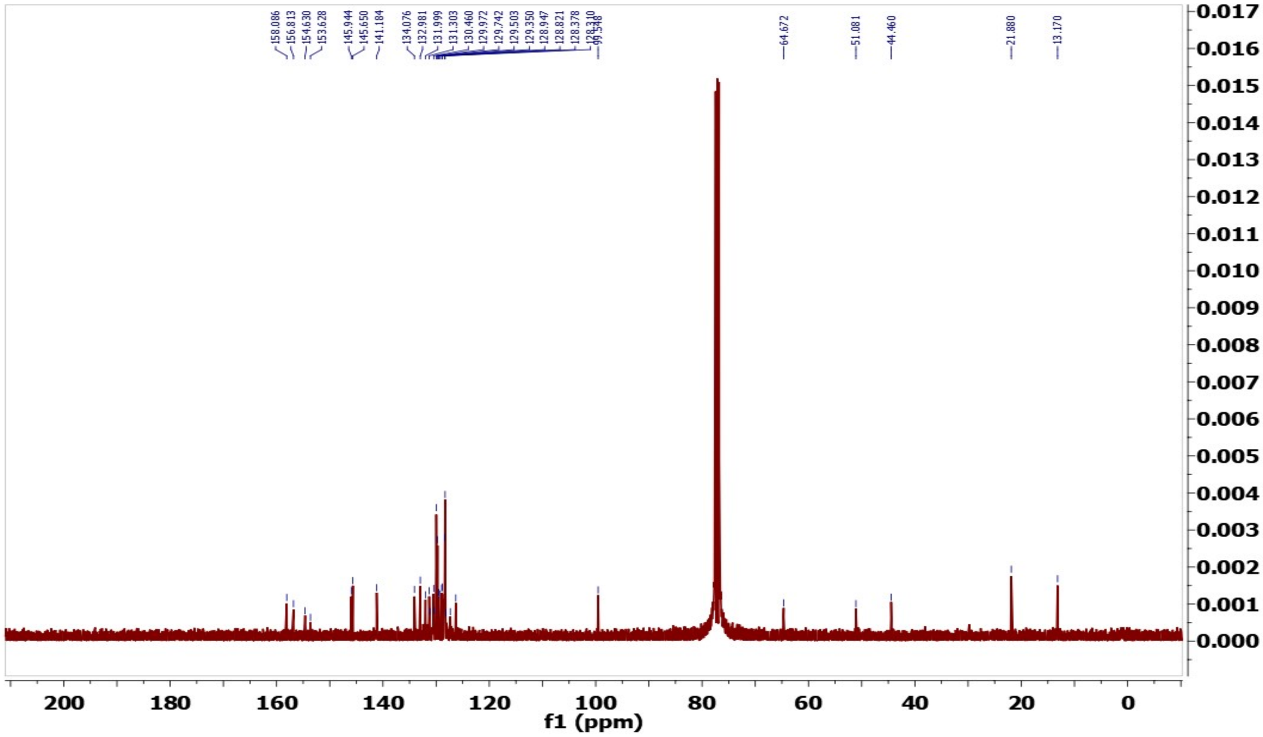

Figure 43. Carbon-13 NMR spectrum of 4g ($CDCl_3$).

Elemental Composition Report

Page 1

Single Mass Analysis
Tolerance = 100.0 PPM / DBE: min = -1.5, max = 50.0
Element prediction: Off
Number of isotope peaks used for i-FIT = 3

Monoisotopic Mass, Even Electron Ions
91 formula(e) evaluated with 1 results within limits (up to 3 closest results for each mass)
Elements Used:
C: 0-34 H: 0-100 N: 0-4 O: 0-3 S: 0-1 I: 0-1

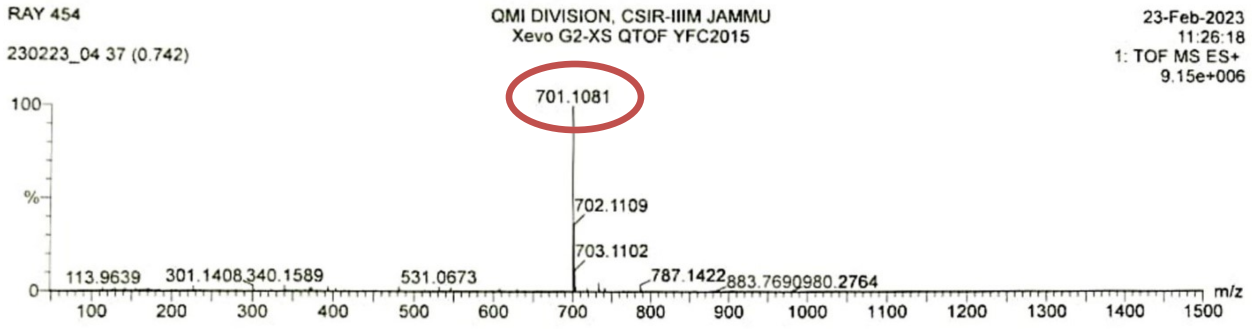

Minimum:				-1.5				
Maximum:		2.0	100.0	50.0				
Mass	Calc. Mass	mDa	PPM	DBE	i-FIT	Norm	Conf(%)	Formula
701.1081	701.1083	-0.2	-0.3	21.5	688.3	n/a	n/a	C34 H30 N4 O3 S I

Figure 44. HRMS Spectrum of 4g

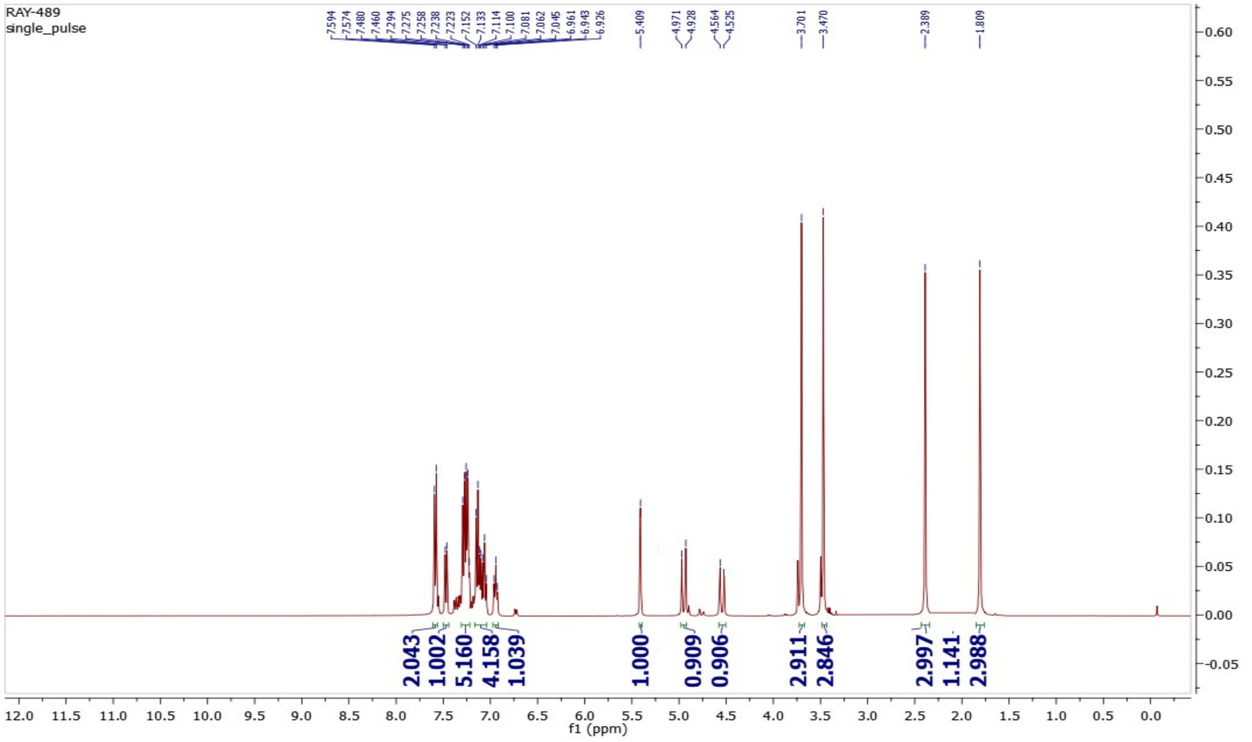

Figure 45. Proton NMR spectrum of 4h ($CDCl_3$).

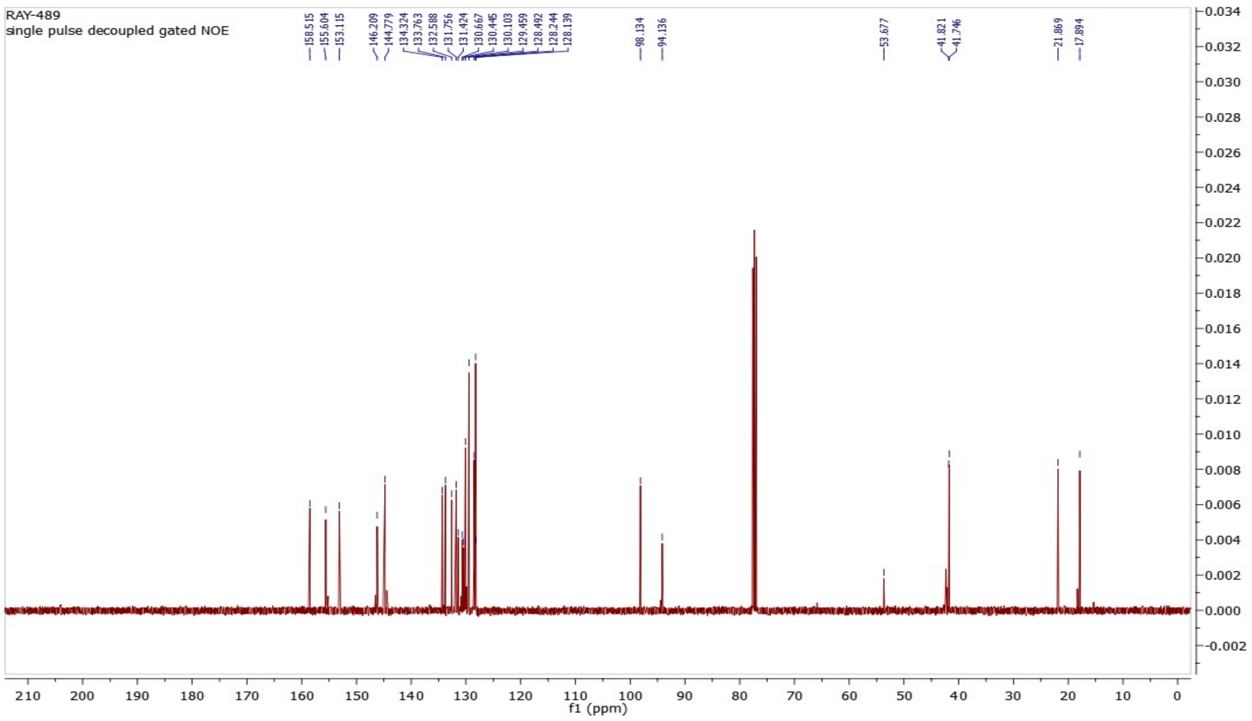

Figure 46. Carbon-13 NMR spectrum of 4h ($CDCl_3$).

Elemental Composition Report Page 1

Single Mass Analysis
Tolerance = 100.0 PPM / DBE: min = -1.5, max = 50.0
Element prediction: Off
Number of isotope peaks used for i-FIT = 3

Monoisotopic Mass, Even Electron Ions
89 formula(e) evaluated with 1 results within limits (up to 3 closest results for each mass)
Elements Used:
C: 0-29 H: 0-100 N: 0-4 O: 0-3 S: 0-1 Br: 0-1
RAY 489
QMI DIVISION, CSIR-IIIM JAMMU
Xevo G2-XS QTOF YFC2015
23-Feb-2023
11:28:51
1: TOF MS ES+
1.11e+007
230223_05 37 (0.741)

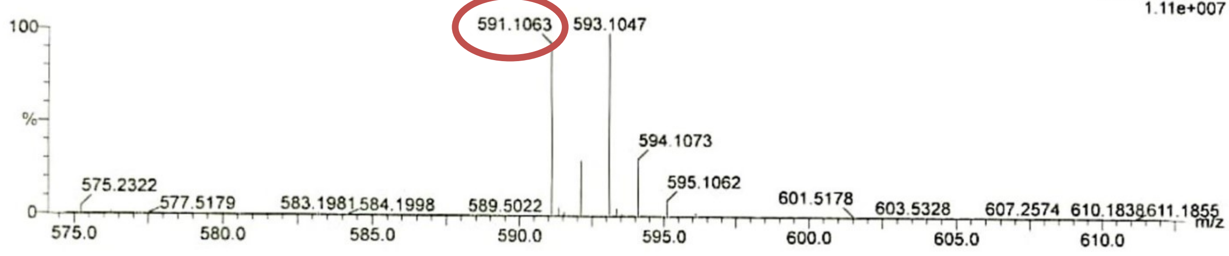

Minimum:				-1.5				
Maximum:		2.0	100.0	50.0				
Mass	Calc. Mass	mDa	PPM	DBE	i-FIT	Norm	Conf(%)	Formula
591.1063	591.1065	-0.2	-0.3	17.5	710.0	n/a	n/a	C29 H28 N4 O3 S Br

Figure 47. HRMS Spectrum of 4h

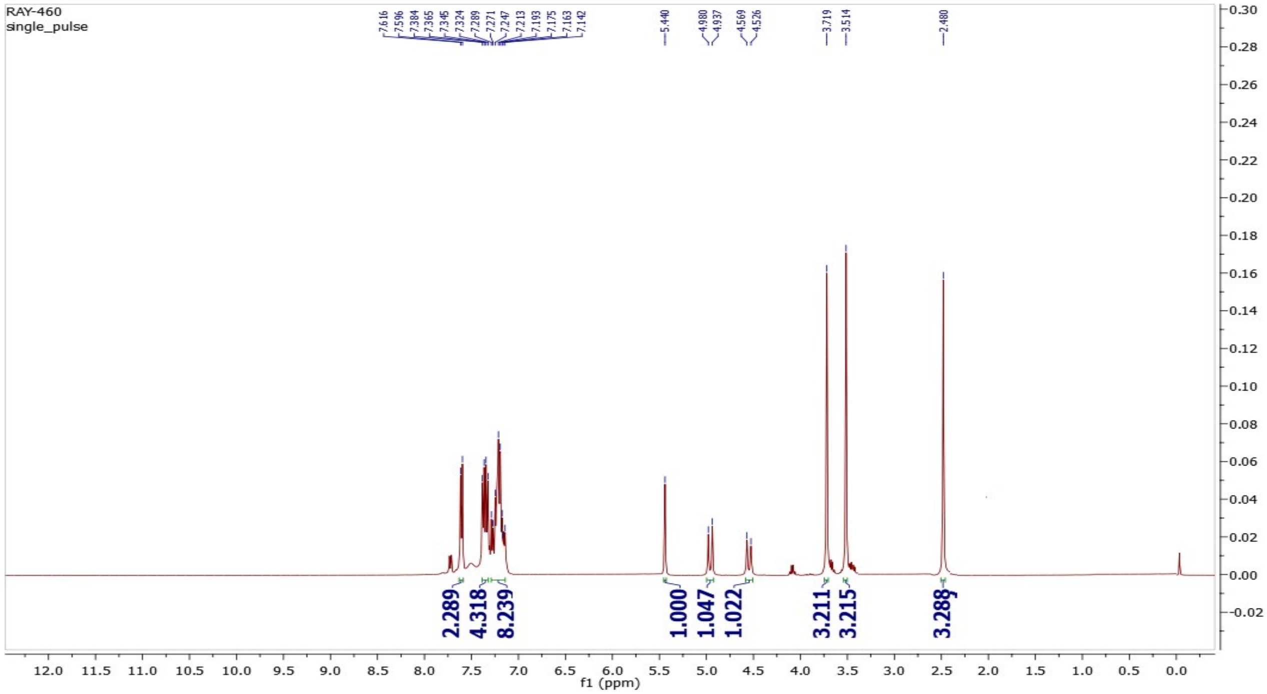

Figure 48. Proton NMR spectrum of 4i ($CDCl_3$).

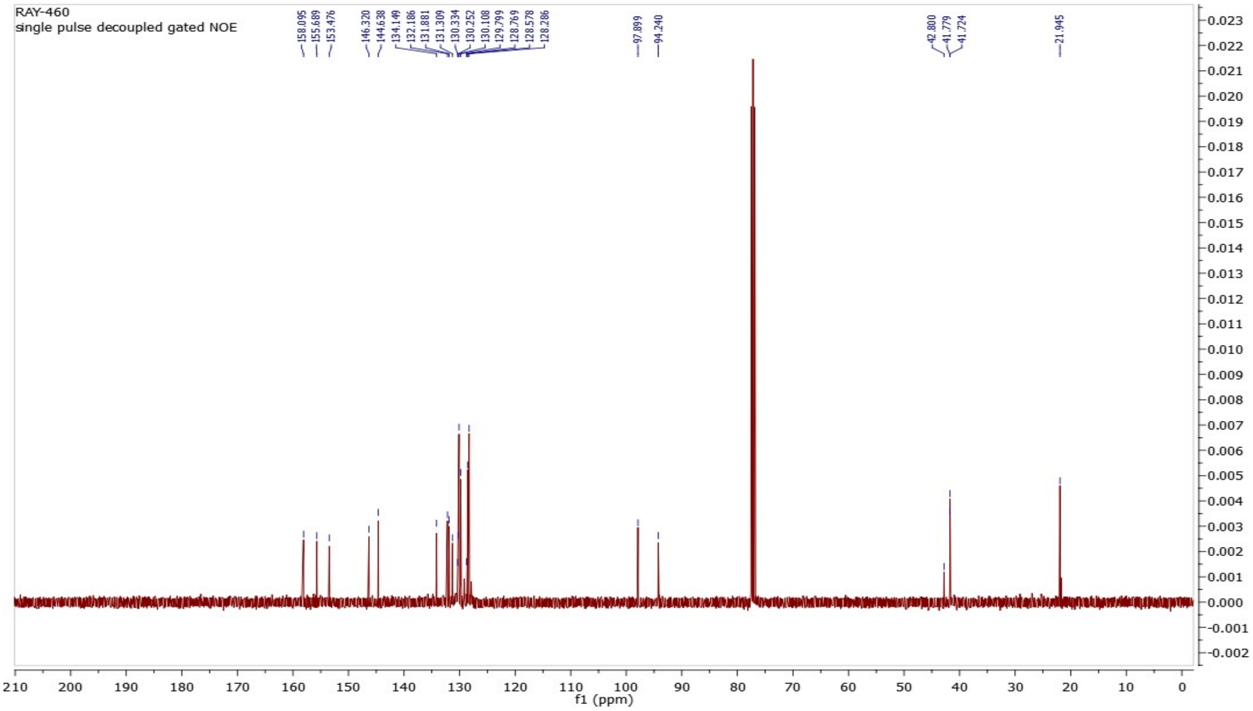

Figure 49. Carbon-13 NMR spectrum of 4i ($CDCl_3$).

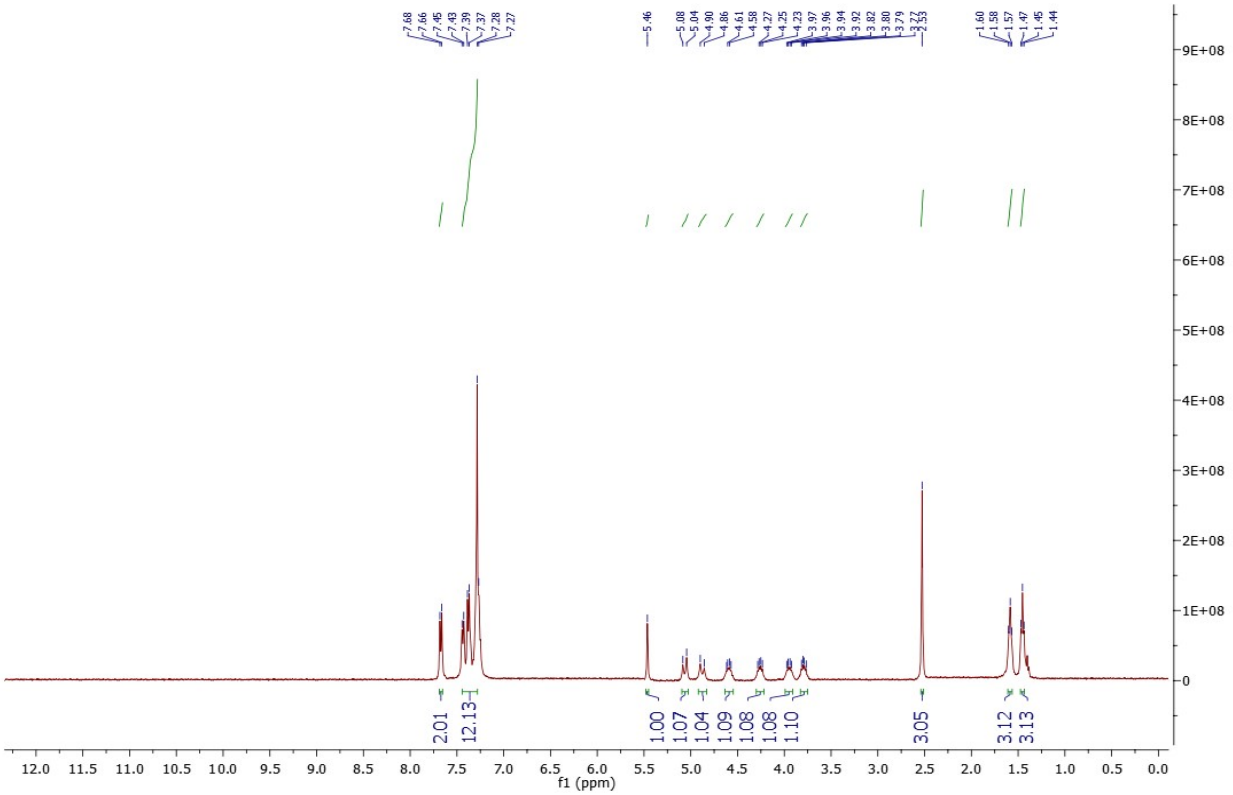

Figure 50. Proton NMR spectrum of 4j ($CDCl_3$).

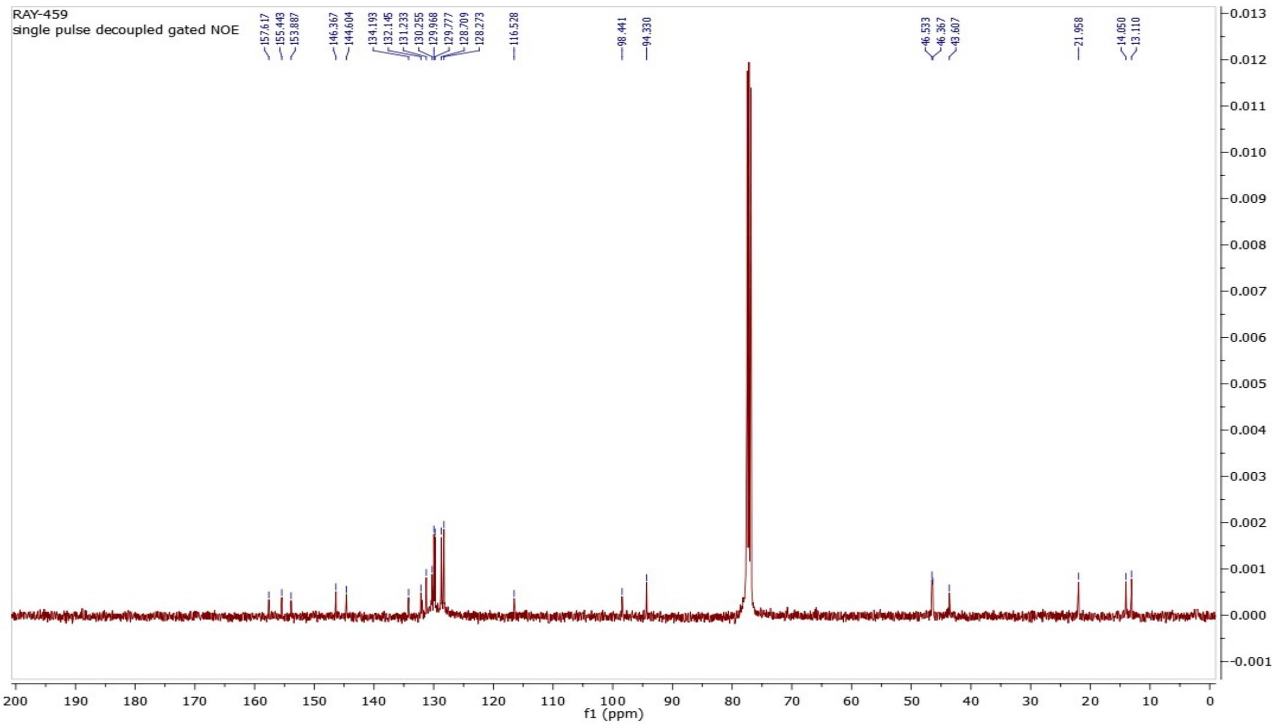

Figure 51. Carbon-13 NMR spectrum of 4j ($CDCl_3$).

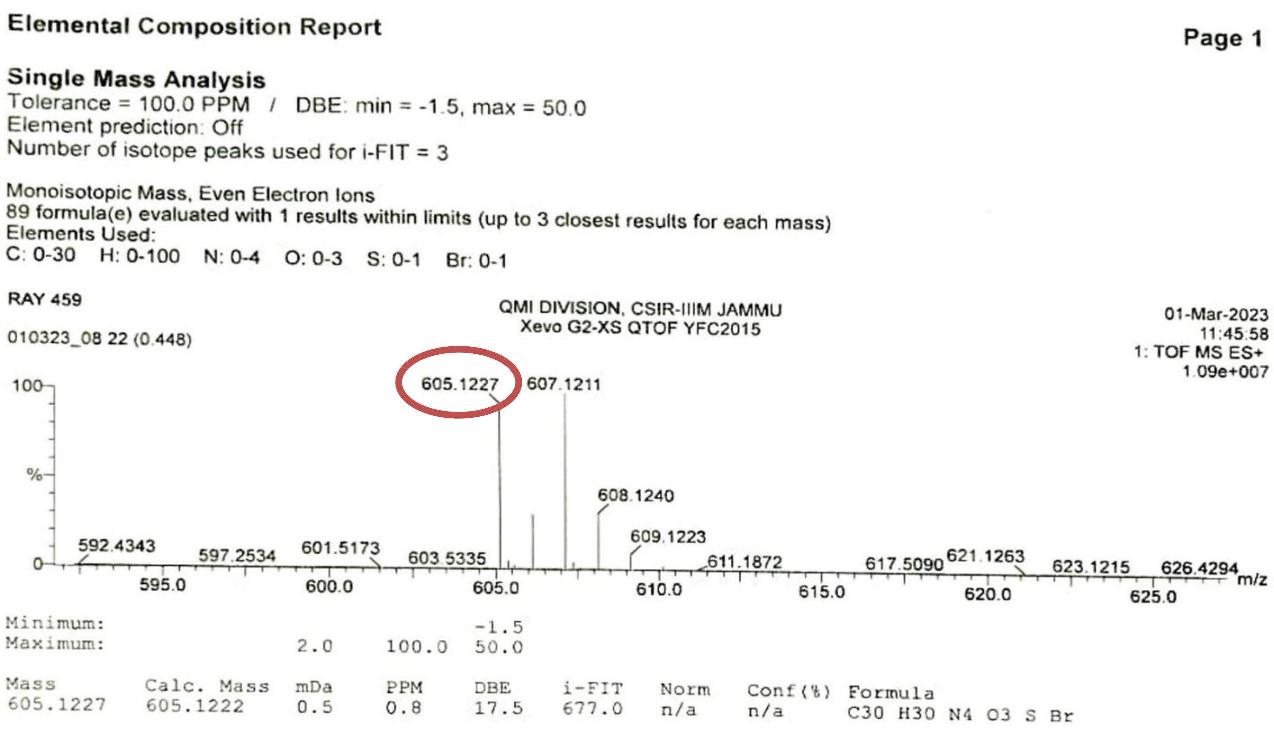

Figure 52. HRMS Spectrum of 4j

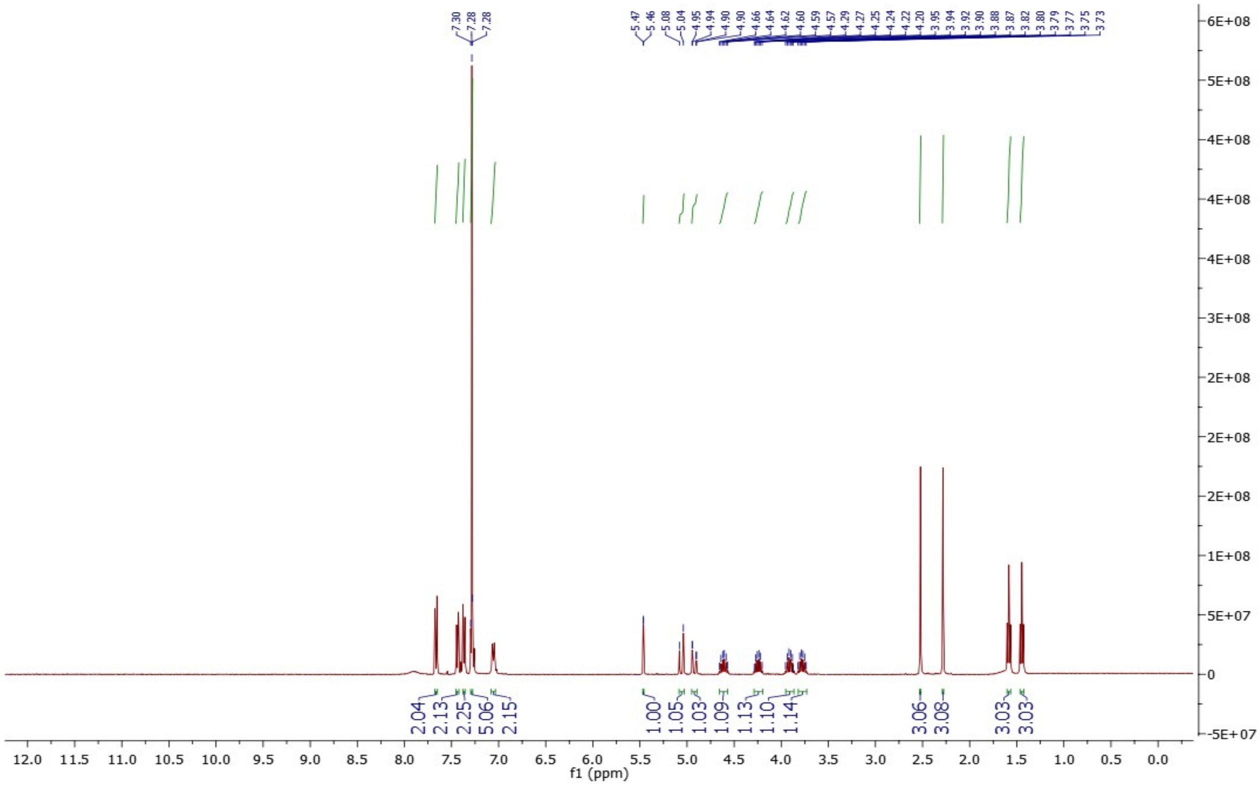

Figure 53. Proton NMR spectrum of 4k ($CDCl_3$).

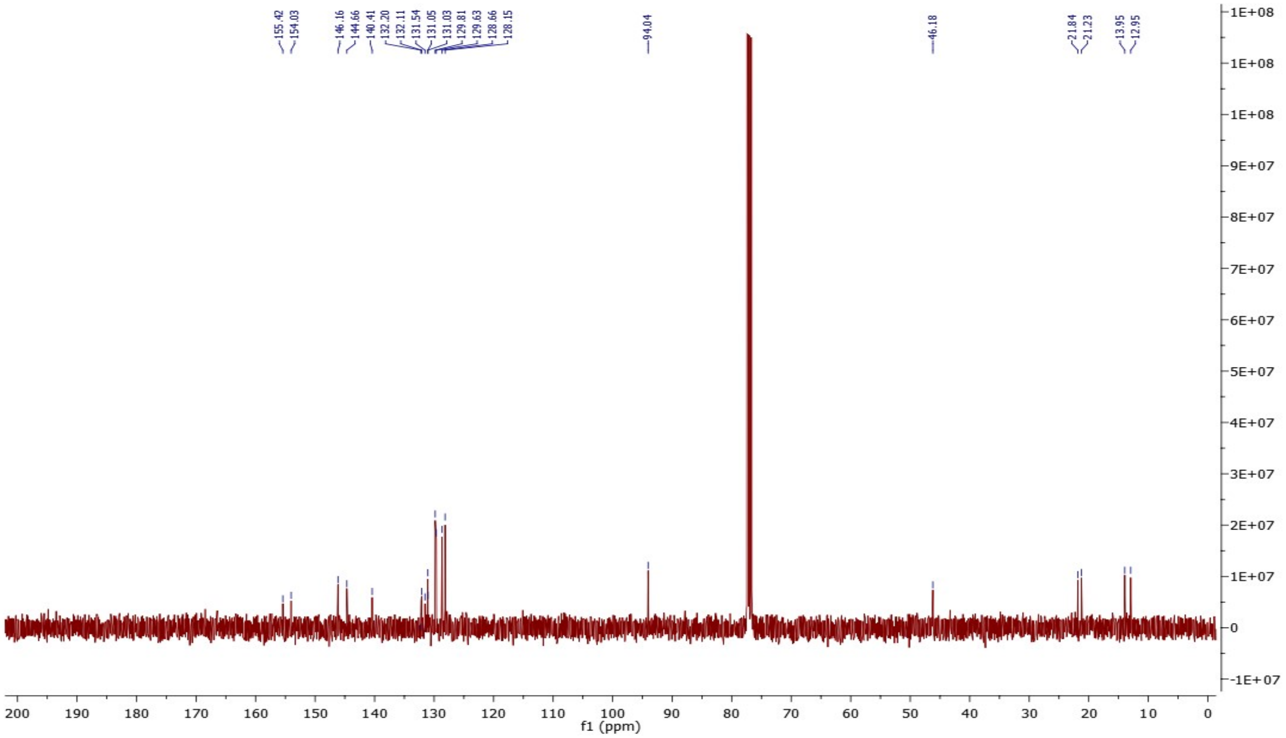

Figure 54. Carbon-13 NMR spectrum of 4k ($CDCl_3$).

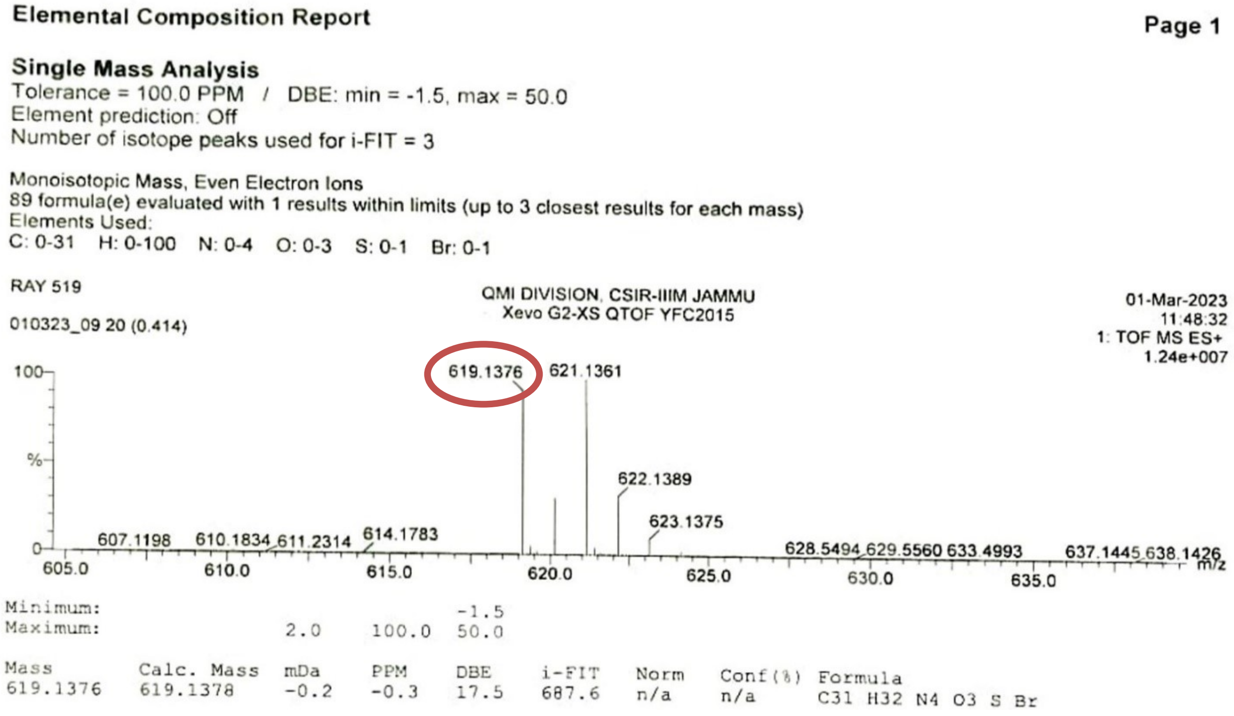

Figure 55. HRMS Spectrum of 4k ($CDCl_3$).
